# Unexpected Reaction of Dialkyl α-Hydroxy-benzylphosphonates with Dialkyl Phosphites and a Few Related Reactions

**DOI:** 10.1021/acs.joc.4c02355

**Published:** 2024-12-17

**Authors:** Zsuzsanna Szalai, Péter Ábrányi-Balogh, György Keglevich

**Affiliations:** a Department of Organic Chemistry and Technology, Faculty of Chemical Technology and Biotechnology, Budapest University of Technology and Economics, Műegyetem rkp. 3., Budapest 1111, Hungary; b Medicinal Chemistry Research Group, HUN-REN Research Centre for Natural Sciences, Budapest 1117, Hungary; c National Drug Research and Development Laboratory, HUN-REN Research Centre for Natural Sciences, Budapest 1117, Hungary

## Abstract

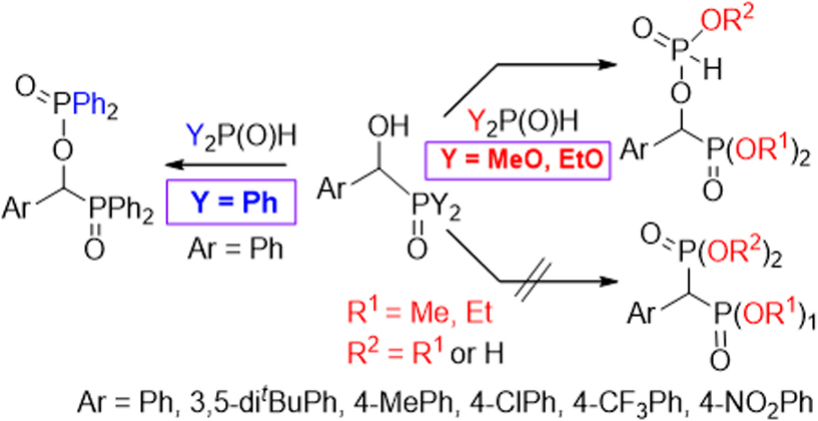

The condensation of dialkyl α-hydroxy-benzylphosphonates with dialkyl phosphites and that of α-hydroxybenzyl-diphenylphosphine oxide with diphenylphosphine oxide unexpectedly gave the corresponding phosphorylated α-hydroxy derivatives. This new reaction proved to be general. The formation of the two products may be similar and involves the attack of the hydroxy group of the α-hydroxyphosphonate or α-hydroxyphosphine oxide on the phosphorus atom of the trivalent tautomer form (Y_2_POH) of the Y_2_P(O)H reagent (Y= MeO, EtO, or Ph) going with the elimination of an alcohol and water molecule, respectively. The mechanism was supported by DFT computations at the M062*X*/6-31G (d,p) level of theory, including suitable proton transfer networks. The condensations discussed are typical autocatalytic reactions promoted by the alcohol or water molecules formed. The initial promoters are the traces of water inevitably present in the mixture. In the reaction of α-hydroxyphosphonates with dialkyl phosphites, the −P(O)(OR)H derivative is the primary product that is partially hydrolyzed to the −P(O)(OH)H species by the traces of water under the conditions of the reaction. Arbuzov reaction of diethyl α-bromobenzylphosphonate with ethyl diphenyphosphinite afforded the target-like phenylmethylene-phosphine oxide—phosphonate derivative.

## Introduction

α-Hydroxyphosphonates make up a representative class of organophosphorus compounds. There are many biologically active derivatives among them.^1−13^ A number of α-hydroxyphosphonates were described with enzyme inhibitory properties.^1^ Inhibitors of CD45 tyrosine phosphatase,^2^ undecaprenyl diphosphate phosphatase,^3^ and P5C reductase^4^ were disclosed. α-Hydroxyphosphonic acids may also be ligands binding in the SH2 domain of Src on coprotein.^5^ Antibacterial,^6,7^ antifungal,^6^ antimicrobial,^8,9^ and antioxidant^10,11^ effects were also reported. Last but not least, the compounds under discussion displayed a cytotoxic effect^12^ and could be used as pesticides.^13^

The major method for the synthesis of α-hydroxyphosphonates that is the addition of dialkyl phosphites to the carbonyl group of aldehydes and ketones, i.e., the Pudovik reaction,^14−23^ may be realized in a lot of ways including green chemical approaches.^24^ Microwave (MW)-assisted^25^ and solvent-free syntheses were also elaborated. However, the workup of the solid phase preparations included extraction, chromatography, and recrystallizations that require considerable amounts of solvents.^26−32^ One of the “greenest” methods was developed by the group of the senior author of this article. According to this, by applying 5% triethylamine as the catalyst and a minimum amount of acetone as the solvent, the Pudovik reaction of benzaldehyde derivatives and dialkyl phosphites was complete after a few hours stirring at the boiling point. On cooling, the hydroxyphosphonate crystallized out from the mixture.^33^

**Scheme 1:**
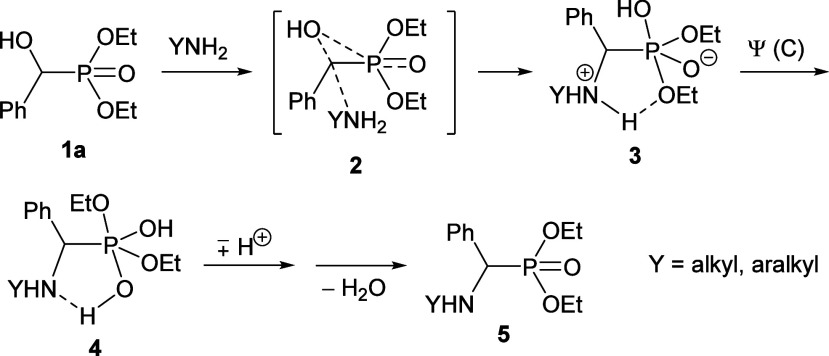
Neighboring Group Effect in the Substitution Reaction of Diethyl α-Hydroxy-benzylphosphonate with Amines^40^

The many-sided reactivity of α-hydroxyphosphonates is noteworthy.^14^ The compounds under discussion were modified by *O*-alkylation,^14^ acylation,^34−36^ and phosphorylation.^37,38^ Rearrangement to phosphates is also a possible option.^39^ Substitution at the carbon atom of the C–OH moiety of α-hydroxyphosphonates by amines could be performed due to the beneficial neighboring group effect of the P=O function, as shown in TS **2**. As a result, an intermediate with a pentavalent pentacoordinated intermediate is formed (**3**) that undergoes a pseudorotation around the C-substituent as the pivot.^40,41^ The phenomenon is shown in Scheme 1.^40^

Encouraged by this substitution reaction, we aimed at the synthesis of phosphonoylphosphonates by the reaction of suitable α-hydroxyphosphonates by dialkyl phosphites. The target products were phenyl-methylenebisphosphonate derivatives, whose analogues are of great importance due to their biological activities.^42^

## Results and Discussion

**Scheme 2:**
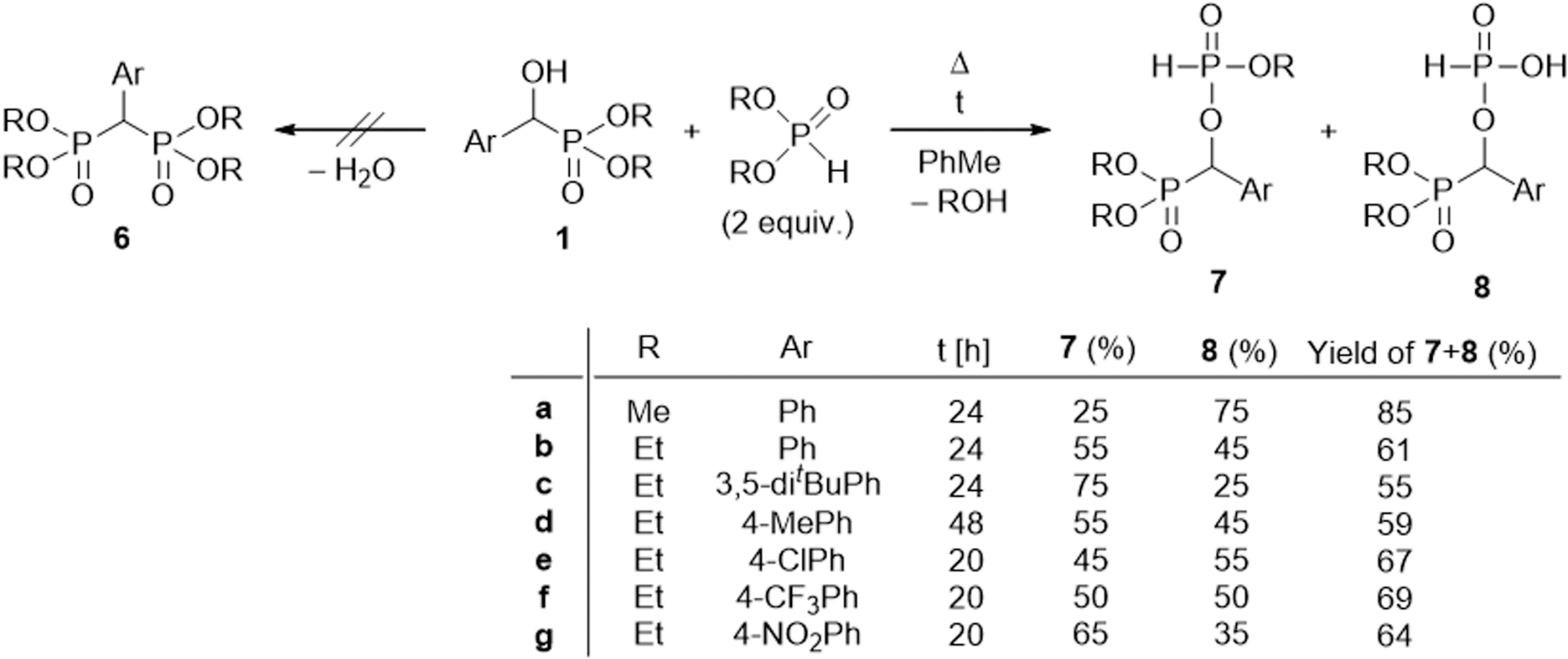
Unexpected Outcome for the Condensation of Dialkyl α-Hydroxy-arylphosphonates (**1a**–**g**) with Dialkyl Phosphites

To prepare tetraalkoxy phenylmethylene-bisphosphonates (**6**), dialkyl α-hydroxy-benzylphosphonates (**1a** and **1b**) were reacted with the corresponding dialkyl phosphite (dimethyl phosphite and diethyl phosphite, respectively). However, measuring in 2 equiv of the P-reagent, and refluxing the toluene solution for 24 h, not the desired bisphosphonate **6** was formed, but the unexpected “phosphorylated” α-hydroxy-benzylphosphonates **7a** and **8a** or **7b** and **8b**, respectively, were present in comparable quantities formed in complete conversions. The compositions and yields are listed in Scheme 2. As an extension, the condensation reaction of diethyl α-hydroxy-3,5-di-*tert*-butylbenzylphosphonate (**1c**), diethyl α-hydroxy-4-methylbenzylphosphonate (**1d**), diethyl α-hydroxy-4-chlorobenzylphosphonate (**1e**), diethyl α-hydroxy-4-trifluoromethylbenzylphosphonate (**1f**), and diethyl α-hydroxy-4-nitrobenzylphosphonate (**1g**) was also attempted with diethyl phosphite, and sure enough, the condensation was found to be general, as the mixtures comprising the comparable quantities of **7c**–**g** and **8c**–**g** were formed also in these instances (Scheme 2). One may see that the electron-releasing methyl substituent in position 4 of the phenyl ring decreased, while the electron-withdrawing 4-Cl, 4-CF_3_, and 4-NO_2_ substituents somewhat increased the reactivity of the α-hydroxyphosphonates (**1**). The 4-Cl, 4-CF_3_, and 4-NO_2_ substituents in the phenyl ring enhance the deprotonation ability of the hydroxy group.

Compound **8** is a dealkylated derivative of ester **7**. Species **8** may have been formed by hydrolysis from **7** under the conditions of the reaction (110 °C) due to the effect of the inevitably present traces of water in the mixture. This was proved by an experiment, where the condensation of benzylphosphonate **1b** with diethyl phosphite was performed in the presence of 1 equiv of water in boiling toluene for 24 h, as the original 55:45% ratio of products **7b** and **8b** was shifted to 40:60%.

Purification by preparative HPLC afforded **7a**–**g**/**8a**–**g** mixtures. The individual species were fully characterized by ^31^P, ^13^C, and ^1^H NMR, as well as HRMS. Product **7b** could be separated by HPLC from the mixture in a pure form. At the same time, species **8b** (together with **8a**/**8c**–**g**) proved to be unstable due to the −P(O)(OH)H function, as it may have been oxidized to −P(O)(OH)_2_, and, as a consequence, **8b** (together with **8a**/**8c**–**g**) could not be isolated in a pure form. In the LC-MS spectra of the crude mixtures, the corresponding phosphonic derivatives (e.g., PhCH(OP(O)(OH)_2_)P(O)(OEt)_2_) could be detected ([M + H]^+^ = 325 and [M − H]^−^ = 323). As such, derivatives **7** and **8** represent a brand new class of compounds. The starting hydroxyphosphonate **1c** is also new.

Products **7a**–**g** and **8a**–**g** exhibited two doublets of 22–28 Hz in the ^31^P NMR spectra. The proton nondecoupled ^31^P NMR spectra revealed a ^1^*J*_PH_ of 714–728 Hz justifying the presence of the P(O)H function in compounds **7** and **8**. The doublet of doublet at δ_C_ ∼73.0 in the ^13^C NMR spectra of compounds **7** and **8** with couplings of ^1^*J*_PC_ = 168–173 Hz and ^2^*J*_PC_ = 6–7 Hz confirmed the P–C–O–P motif of products **7** and **8**. The ^1^H NMR spectra were also useful in affirming the large ^1^*J*_PH_ couplings of 714–728 Hz for the >P(O)H moiety. The new products **7** and **8** show some resemblance to the phosphinoyl-oxyphosphonates prepared by the reaction of α-hydroxy-benzylphosphonates with phosphinoyl chlorides.^37,38^ Another approach, the DMAP-catalyzed bisphosphorylation of anhydrides with secondary phosphine oxides also led to derivatives with the P–O–C–P motif.^43^

**Figure 1:**
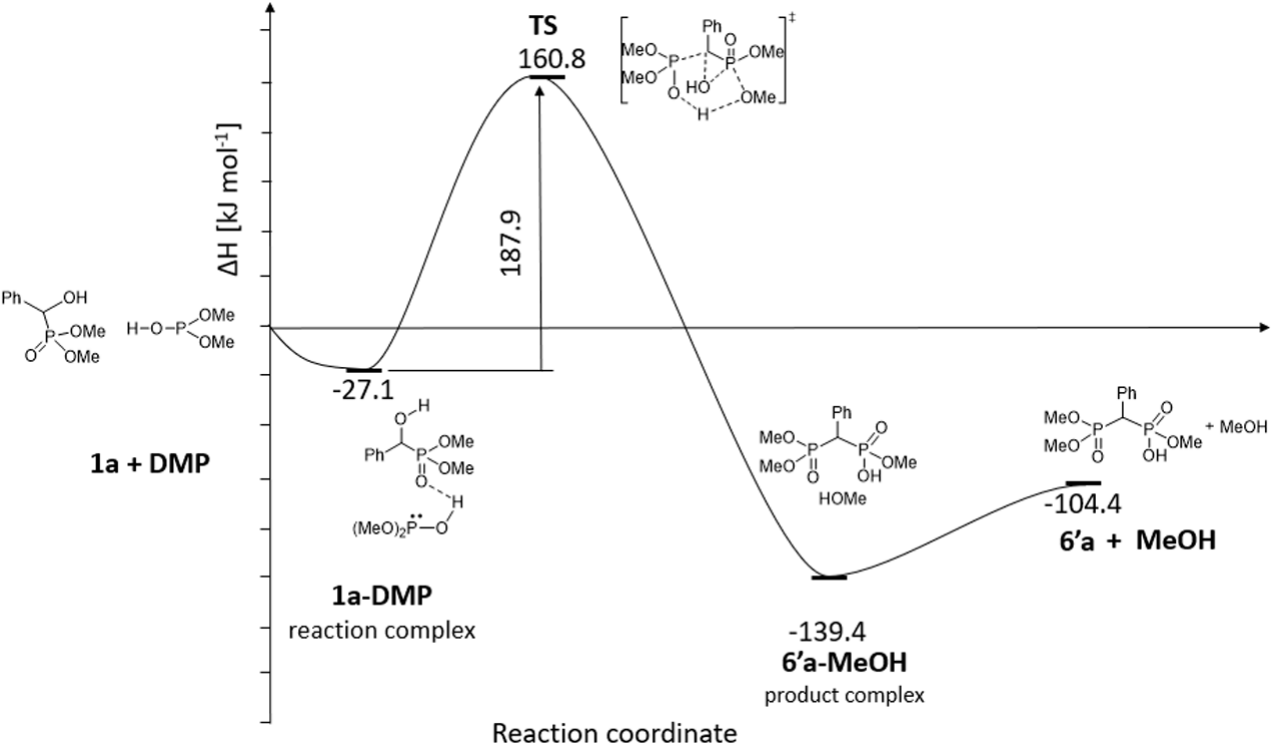
Enthalpy diagram for the unrealistic condensation of hydroxyphosphonate **1a** with dimethyl phosphite.

Calculations suggested that the originally expected hydroxyphosphonate **1a** → bisphosphonate **6a** transformation is unfavorable due to the 187.9 kJ mol^–1^ high enthalpy of activation, although the thermodynamics for the addition step is favorable (Δ*H* = −104.4 kJ mol^–1^) (Figure 1). Notably, it was observed that **6a** is not the product, but its demethylated derivative **6′a**. This phenomenon may be explained by the better leaving ability of the methoxy group compared to the hydroxy unit.

**Scheme 3:**
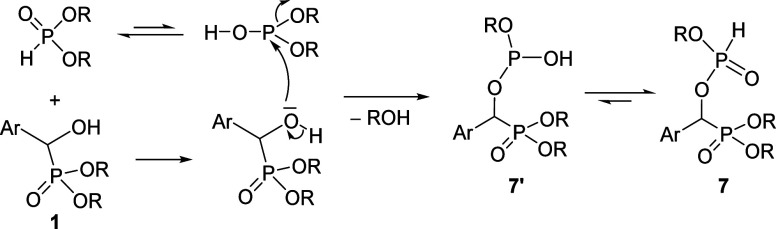
Proposed Mechanism for the Formation of Phosphorylated α-Hydroxy-benzylphosphonates (**7**)

The unexpected condensation reaction discovered by us is completely new. A putative mechanism is shown in Scheme 3. It was assumed that the oxygen atom of the α-hydroxy phosphonate **1** attacks the phosphorus atom of the trivalent tautomer form (>POH) of the reagent (>P(O)H). This is accompanied by the departure of an alcohol molecule to furnish the tautomeric form (**7′**) of α-alkoxyphosphoryloxy-benzylphosphonate (**7**).

We have computed the energetics of the proposed mechanistic pathway by DFT calculations at the M062*X*/6-31+(d,p) level of theory considering the solvent effect of toluene (SMD implicit solvent model) and 383 K as the temperature using the Gaussian 16 program package. We have chosen α-hydroxyphosphonate **1a** and dimethyl phosphite as reactants and an additional MeOH molecule to facilitate the proton transfer based on our previous studies on the esterification of P-acids leading to phosphonates and phosphates.^44,45^

**Scheme 4:**
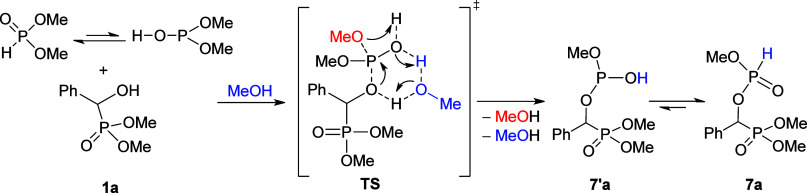
Computed Mechanism for the Formation of Phosphorylated α-Hydroxy-benzylphosphonate (**7a**)

We observed a concerted reaction where the oxygen atom of α-hydroxyphosphonate **1a** attacks the phosphorus atom of the trivalent tautomer form of the reagent, while a methanol molecule leaves the phosphite moiety. This is practically a nucleophilic substitution. The added methanol molecule acts as a proton acceptor and proton donor in the network; hence, the extra MeOH has an important role during the formation of product **7′a**. The proton transfers via the network shown make an energetically favorable transformation possible (Scheme 4).

The condensation is an autocatalytic transformation, as the methyl alcohol formed in the “first cycle” is needed in the TS of the “second cycle”. In the initial stage (in the first cycle), the missing MeOH molecules may be substituted by traces of water. The role of MeOH was proved, as repeating the condensation of hydroxyphosphonate **7a** with dimethyl phosphite in the presence of 1 equiv of MeOH; the condensation was faster (i.e., the completion required 16 h instead of 24 h).

**Figure 2:**
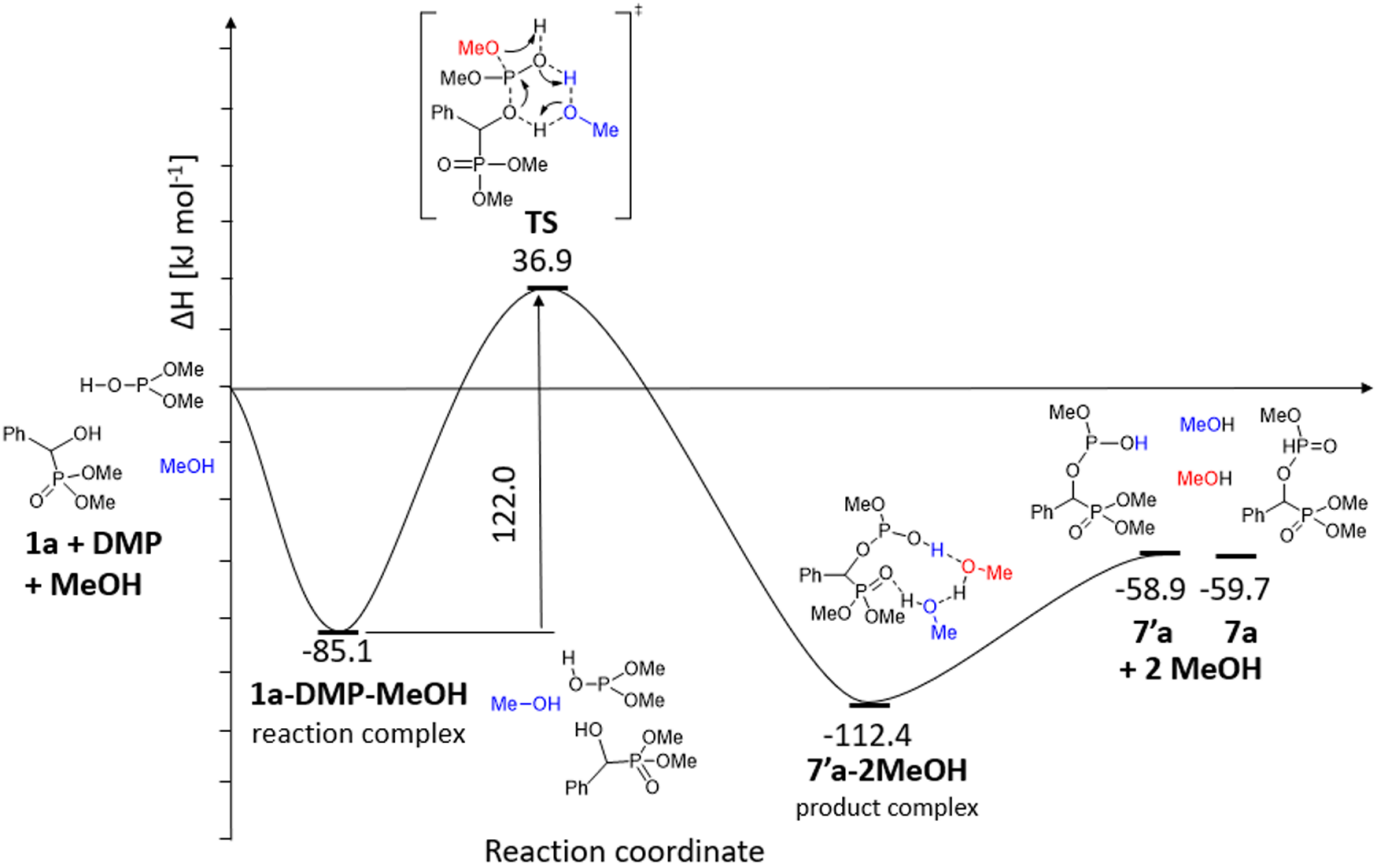
Enthalpy diagram for **1a** → **7a** transformation.

We have computed the enthalpy and Gibbs free energy profile of the reaction and found that the tautomerism of (MeO)_2_P(O)H to the trivalent (MeO)_2_POH (DMP) form is slightly endothermic (Δ*H* = 7.2 kJ mol^–1^ and Δ*G* = 0.3 kJ mol^–1^). It is assumed that both forms are present in refluxing toluene. In the first step, the reaction complex comprising the hydroxyphosphonate (**1a**), the phosphite, and the MeOH molecule is formed (Δ*H* = −85.1 kJ mol^–1^ and Δ*G* = 39.8 kJ mol^–1^). The transition state (Δ*H* = 122.0 kJ mol^–1^ and Δ*G* = 136.4 kJ mol^–1^) belonging to the concerted reaction may be overcome under the conditions applied (110 °C/24 h). The product complex containing **7′a** and two MeOH molecules has a lower energy (ΔΔ*H* = −27.3 kJ mol^–1^ and ΔΔ*G* = −20.9 kJ mol^–1^) than the starting complex due to H-bridge interactions. Comparison of the energies of the starting reactants and those of the product suggests a more significant exothermicity (Δ*H* = −58.9 kJ mol^–1^ and Δ*G* = −42.8 kJ mol^–1^). This may be a driving force for the reaction under discussion. Comparing **7a** and **7′a**, there is no significant difference neither in enthalpy (Δ*H* = −0.8 mol^–1^) nor in Gibbs free energy (Δ*G* = 2.2 kJ mol^–1^). This is presumably due to the intramolecular hydrogen bond between the P–OH and P=O functions in **7′a** decreasing the energy gain of the >P–OH → >P(O)H tautomerism (Figure 2).

**Scheme 5:**
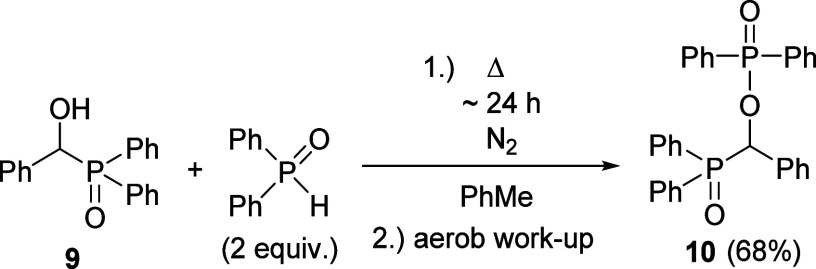
Reaction of Diphenyl α-Hydroxy-benzylphosphine Oxide **9** with Diphenylphosphine Oxide

As a logical continuation, the reaction of diphenyl-(α-hydroxybenzyl)phosphine oxide (**9**) with diphenylphosphine oxide was also tried that was carried out as the condensation of dialkyl α-hydroxy-benzylphosphonates (**1**) with dialkyl phosphites (24 h reflux in toluene). However, an inert atmosphere was ensured to avoid the oxidation of Ph_2_P(O)H. The workup and purification by preparative HPLC chromatography furnished, on the basis of ^31^P, ^13^C, and ^1^H spectral data, α-(diphenylphosphinoyloxy)benzyl-diphenylphosphine oxide (**10**) (Scheme 5).

**Scheme 6:**
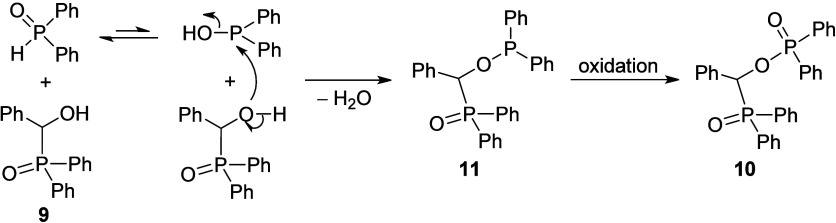
Proposed Mechanism for the Formation of Phosphinoyloxy-benzyl-diphenylphosphine Oxide (**10**)

In this case, the first step is the attack of the oxygen atom of the hydroxy group of the α-hydroxybenzyl-diphenylphosphine oxide (**9**) on the phosphorus atom of the trivalent tautomer of the Ph_2_P(O)H reagent that is connected with the loss of water to provide the phosphinous ester–phosphine oxide intermediate **11**. The latter species (**11**) may undergo oxidation on workup to the phosphinoyloxy-benzyl-diphenylphosphine oxide **10** (Scheme 6).

**Figure 3:**
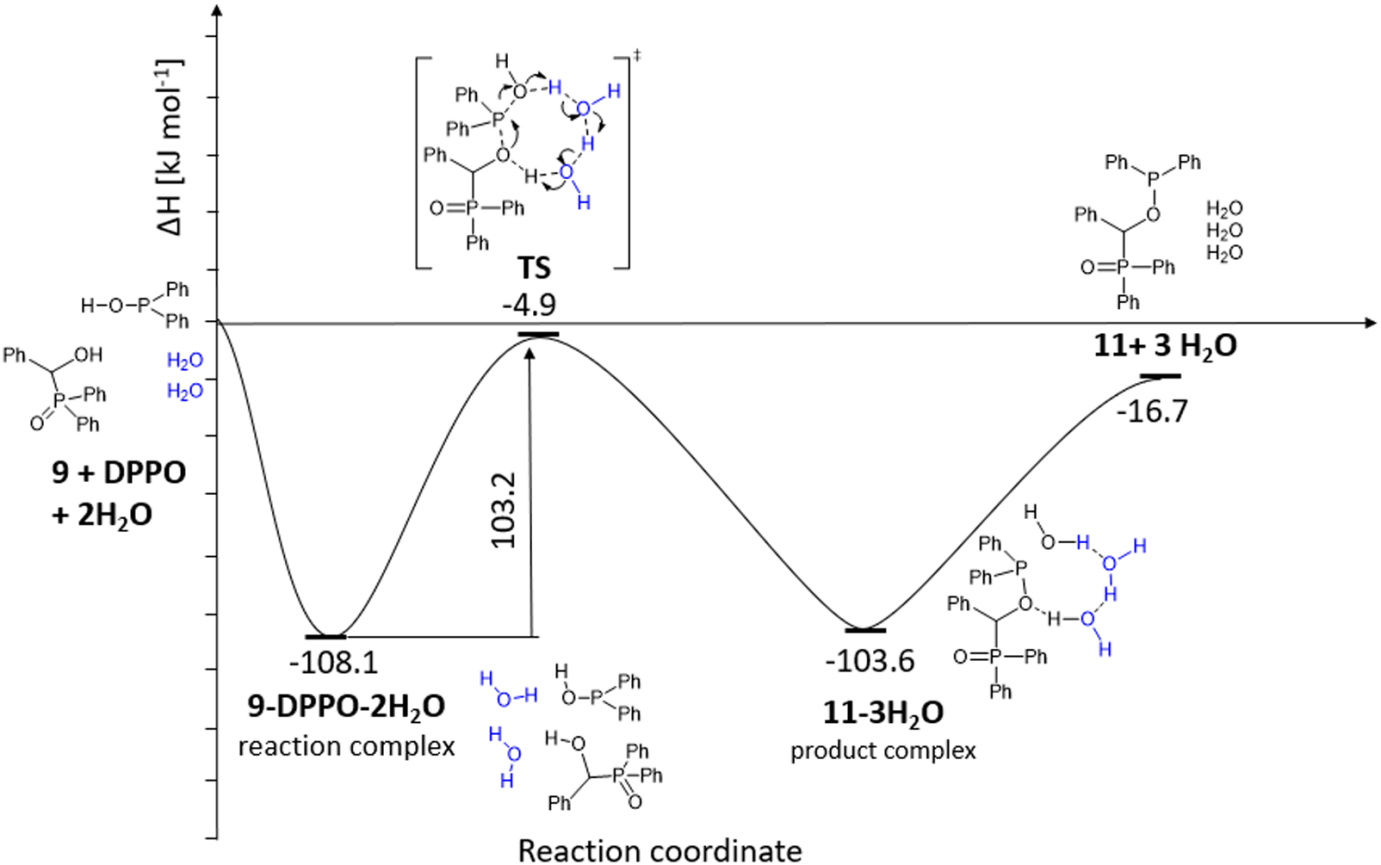
Enthalpy diagram for **9** → **11** transformation.

According to the computations, due to the large size of the phenyl rings, a more complex proton transfer network was necessary for the transformation. Having modeled (a) the two reactants themselves, (b) hydroxyphosphine oxide **9** with two diphenyphosphinous acid (Ph_2_POH) molecules, (c) one additional water molecule, and (d) two water molecules, it was found that only the last model has a realistic enthalpy for the transition state (Figure 3). In this case, the tautomerization of Ph_2_P(O)H to Ph_2_POH is practically thermoneutral (Δ*H* = 1.2 kJ mol^–1^ and Δ*G* = −2.8 kJ mol^–1^). Similarly to the **1a** → **7a** transformation, in the first step, the reaction complex of hydroxyphosphine oxide **9**, Ph_2_POH, and two water molecules is formed (Δ*H* = −108.1 kJ mol^–1^ and Δ*G* = 58.2 kJ mol^–1^). The higher Gibbs free energy as compared to that observed in the previous reaction might be a consequence of the complexation of four species decreasing the entropy. The energy requirement of the transition state (Δ*H* = 103.2 kJ mol^–1^ and Δ*G* = 149.1 kJ mol^–1^) is comparable with that found for the reaction of hydroxyphosphonate **1a** and (MeO)_2_P(O)H, but the formation of the product complex containing species **11** and three water molecules from the reaction complex is, in this case, slightly endothermic (Δ*H* = 4.5 kJ mol^–1^ and Δ*G* = 11.3 kJ mol^–1^). However, comparing the enthalpy level of the starting reactants and phosphinous ester–phosphine oxide intermediate **11**, the overall transformation is exothermic (Δ*H* = −16.7 kJ mol^–1^), while according to the Gibbs free energy, it is thermoneutral (Δ*G* = 2.1 kJ mol^–1^). The formation of the crowded structure decreases the entropy that is balanced by the enthalpy gain caused by the stabilizing π–π interactions between the phenyl rings, which may mean a driving force for the transformation under discussion.

The **9** → **11** transformation is again an autocatalytic process, as the water molecules formed in the initial stage of the reaction are needed to be incorporated into the corresponding TS. In the initial stage, the traces of water present in the mixture may play this role.

It is noteworthy that, in the reaction of dimethyl α-hydroxy-benzylphosphonate **1a** with dimethyl phosphite, a TS with a 6-membered is formed including one additional methyl alcohol molecule, while in the condensation of diphenyl-(α-hydroxybenzyl)phosphine oxide (**9**) with diphenylphosphine oxide, the TS is of an 8-membered ring incorporating two water molecules. The reason is that in the latter case, the sterical hindrance due to the presence of the phenyl groups allows only the establishment of an 8-membered ring.

**Scheme 7:**
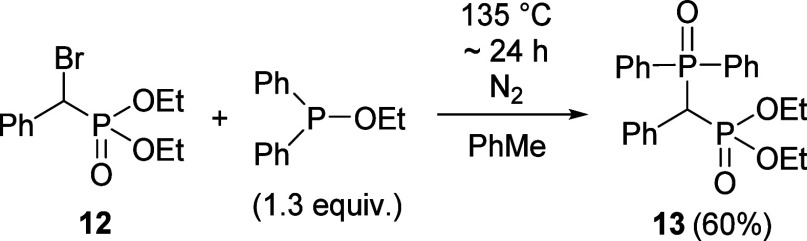
Reaction of Diethyl Bromo(phenyl)methylphosphonate with Ethoxydiphenylphosphine

Last but not least, diethyl α-bromobenzylphosphonate (**12**) was reacted in the Arbuzov reaction with ethyl diphenylphosphinite to afford the type of original target analogue phenylmethylene-phosphine oxide-phosphonate derivative **13** that was isolated and fully characterized (Scheme 7). Carrying out the Arbuzov reaction starting from 2.0 mmol of bromophosphonate **12**, the yield was 60%.

## Conclusions

To summarize our results, an unexpected reaction of dialkyl α-hydroxy-benzylphosphonates with dialkyl phosphites furnishing “phosphorylated” α-hydroxy-benzylphosphonates was observed. The mechanism supported by DFT calculations involved the nucleophilic attack of the oxygen atom of the hydroxy group of the hydroxyphosphonate on the phosphorus atom of the trivalent tautomer form of dialkyl phosphite, providing the tautomer form of the final alkoxyphosphoryloxy-benzylphosphonate. A novel proton transfer network made possible the transformation. Interestingly, the condensation of diphenyl-(α-hydroxybenzyl)phosphine oxide with diphenylphosphine oxide also led to a similar type of product with the P–C–O–P motif; however, in this case, the final step included the oxidation of the phosphinous ester–phosphine oxide intermediate. In the case of both reaction models investigated, the presence of traces of water was crucial to ensure the start of the autocatalytic reaction. The beneficial effect of 1 equiv of methyl alcohol added to the corresponding reaction mixture prior to heating was proved. A representative of the original targets a phenylmethylene-phosphine oxide-phosphonate derivative was prepared by the Arbuzov reaction of diethyl α-bromobenzylphosphonate with ethyl diphenylphosphinite.

## Experimental Section

### General Information

The ^31^P, ^13^C, and ^1^H NMR spectra were taken on a Bruker DRX-500 or Bruker Avance-300 spectrometer operating at 202, 126, and 500 MHz or 122, 75, and 300 MHz, respectively. The ^31^P chemical shifts are downfield relative to those of H_3_PO_4_, while the ^13^C and ^1^H chemical shifts are downfield relative to those of TMS. The couplings are given in Hz. The exact mass measurements were performed using an Agilent 6545 Q-TOF mass spectrometer (Santa Clara, CA) in high-resolution, positive electrospray mode. The melting point of product **1c** was determined using a Setaram Differential Scanning Calorimetry 92 device. The products were purified by gradient-elution preparative HPLC with UV detection at 214 nm, using water and acetonitrile as the mobile phase components; column type: Gemini 5 μM NX-C18.

### Procedures and Compound Characterization

#### Diethyl α-Hydroxy-3,5-di-*tert*-butylphenyl-methylphosphonate (**1c**)

The mixture of 2.0 mmol (0.44 g) of 3,5-di-*tert*-butylbenzaldehyde, 2.0 mmol (0.26 mL) of diethyl phosphite, and 0.20 mmol (27.9 μL) of triethyl amine in acetone (0.5 mL) was stirred at reflux. After 240 min, 3 mL of pentane was added to the reaction mixture, and it was cooled to 5 °C. The product crystallized from the reaction mixture. Filtration of the reaction mixture afforded product **1c** as white crystals in a purity of >99%. Yield: 0.65 g (90%); mp 84–85 °C.

^31^P {^1^H} NMR (202 MHz, CDCl_3_) δ 21.8; ^13^C {^1^H} NMR (126 MHz, CDCl_3_) δ 150.5 (s), 135.6 (s), 121.9 (bs), 121.5 (d, *J* = 5.9 Hz), 71.4 (d, *J* = 158.7 Hz), 63.3 and 62.8 (d, *J* = 7.2 Hz), 34.9 (s), 31.5 (s), 16.4 (t, *J* = 6.2 Hz); ^1^H NMR (500 MHz, CDCl_3_) δ 7.39–7.34 (m, 3H), 5.03 (d, 1H, *J* = 1.3 Hz, 4.10–3.93 (m, 4H), 1.35 (s, 18H), 1.29 and 1.21 (t, 6H, *J* = 6.9 Hz); HRMS (*m*/*z*): [M + Na]^+^ Calcd for C_19_H_33_O_4_PNa: 379.2014; Found 379.2007.

#### General Procedure for the Preparation of Dialkyl α-Alkoxyphosphoryloxy-benzylphosphonates (**7a**–**g**) and Dialkyl α-Hydroxyphosphoryloxy-benzylphosphonates (**8a**–**g**)

The mixture of 1.0 mmol of dialkyl α-hydroxy-α-aryl-methylphosphonate (dimethyl α-hydroxy-α-phenyl-methylphosphonate (**1a**): 0.22 g, diethyl α-hydroxy-α-phenyl-methylphosphonate (**1b**): 0.24 g, diethyl α-hydroxy-3,5-di-*tert*-butylphenyl-methylphosphonate (**1c**): 0.36 g, diethyl α-hydroxy-4-methylphenyl-methylphosphonate (**1d**): 0.26 g, diethyl α-hydroxy-4-chlorophenyl-methylphosphonate (**1e**): 0.28 g, diethyl α-hydroxy-4-trifluoromethylphenyl-methylphosphonate (**1f**): 0.31 g, diethyl α-hydroxy-4-nitrophenyl-methylphosphonate (**1g**): 0.29 g, and 2.0 mmol of dialkyl phosphite (diethyl phosphite: 0.26 mL, dimethyl phosphite: 0.18 mL) in toluene (13 mL) was stirred at 110 °C. After a 20–48 h reaction time (see in Scheme 2), the solvent was evaporated, and the crude product was purified by reversed-phase HPLC column chromatography (column type: Gemini 5 μM NX-C18), applying acetonitrile–water gradient elution (from 3 to 100% MeCN increasing with 1.5%/min at a flow rate of 30 mL/min) to afford a 25:75 mixture of **7a**/**8a**, a 55:45 mixture of **7b/8b**, a 75:25 mixture of **7c/8c**, a 55:45 mixture of **7d**/**8d**, a 45:55 mixture of **7e**/**8e**, a 50:50 mixture of **7f**/**8f**, and a 65:35 mixture of **7g**/**8g**.

##### Dimethyl α-Methoxyphosphoryloxy-benzylphosphonate (**7a**) and Dimethyl α-Hydroxyphosphoryloxy-benzylphosphonate (**8a**)

Yield: 0.24 g (85%), colorless oil.

**7a** (25%): ^31^P {^1^H} NMR (202 MHz, CDCl_3_) δ_P1_ 19.1 and δ_P2_ 9.4 (d, *J* = 25.9 Hz); ^13^C {^1^H} NMR (75 MHz, CDCl_3_) δ 132.7 (s), 129.33 (d, *J* = 3.9 Hz), 128.8 (d, *J* = 2.1 Hz), 127.68 (d, *J* = 5.9 Hz), 72.5 (dd, *J*_1_ = 171.4 Hz, *J*_2_ = 6.8 Hz), 54.07 (d, *J* = 6.9 Hz), 54.04 (d, *J* = 6.7 Hz), 51.7 (d, *J* = 6.7 Hz); ^1^H NMR (500 MHz, CDCl_3_) δ 7.55–7.38 (m, “a”), 6.97 (d, “b”, *J* = 728.2 Hz), 5.74 (dd, “c”, *J*_1_ = *J*_2_ = 11.9 Hz), 3.82 (d, “d”, *J* = 12.2 Hz), 3.70 (d, “d”, *J* = 10.6 Hz), 3.57 (d, “d”, *J* = 12.2 Hz); HRMS (*m*/*z*): [M + Na]^+^ Calcd for C_10_H_16_O_6_P_2_Na 317.0320; Found 317.0328.

**8a** (75%): ^31^P {^1^H} NMR (202 MHz, CDCl_3_) δ_P1_ 18.8 and δ_P2_ 8.6 (d, *J* = 25.2 Hz); ^13^C {^1^H} NMR (75 MHz, CDCl_3_) δ 132.7 (s), 129.37 (d, *J* = 3.0 Hz), 128.8 (d, *J* = 2.1 Hz), 127.71 (d, *J* = 5.8 Hz), 72.9 (dd, *J*_*1*_ = 172.5 Hz, *J*_*2*_ = 6.5 Hz), 54.09 (d, *J* = 7.1 Hz), 52.0 (d, *J* = 6.5 Hz); ^1^H NMR (500 MHz, CDCl_3_) δ 7.55–7.38 (m, “a”), 6.85 (d, “b”, *J* = 721.8 Hz), 5.70 (dd, “c”, *J*_*1*_ = *J*_*2*_ = 11.1 Hz), 3.78 (d, “d”, *J* = 10.7 Hz), 3.71 (d, “d”, *J* = 10.6 Hz), HRMS (*m*/*z*): [M + Na]^+^ Calcd for C_9_H_14_O_6_P_2_Na 303.0163; Found 303.0168.

“a”: total int. 10H; “b”: total int.: 2H; “c”: total int.: 2H; “d”: total int.: 15H.

##### Diethyl α-Ethoxyphosphoryloxy-benzylphosphonate (**7b**) and Diethyl α-Hydroxyphosphoryloxy-benzylphosphonate (**8b**)

Yield: 0.29 g (61%), colorless oil.

**7b** (55%): ^31^P {^1^H} NMR (202 MHz, CDCl_3_) δ_P1_ 16.7 and δ_P2_ 7.7 (d, *J* = 25.8 Hz); ^13^C {^1^H} NMR (126 MHz, CDCl_3_) δ 133.2 (bs), 129.1 (d, *J* = 2.8 Hz), 128.5 (d, *J* = 2.2 Hz), 127.6 (d, *J* = 5.8 Hz), 72.9 (dd, *J*_1_ = 171.2 Hz, *J*_2_ = 6.8 Hz), 63.60 (d, *J* = 6.6 Hz), 61.8 (d, *J* = 6.5 Hz), 16.36 and 16.3 (d, *J* = 5.7 Hz), 16.0 (d, *J* = 6.6 Hz); ^1^H NMR (500 MHz, CDCl_3_) δ 7.48–7.31 (m, “a”), 6.93 (d, 1H, *J* = 723.9 Hz), 5.66 (dd, 1H, *J*_1_ = *J*_2_ = 12.0 Hz), 4.24–3.83 (m, “b”), 1.31–1.15 (m, “c”); HRMS (*m*/*z*): [M + Na]^+^ Calcd for C_13_H_22_O_6_P_2_Na 359.0789; Found: 359.0789.

**8b** (45%): ^31^P {^1^H} NMR (202 MHz, CDCl_3_) δ_P1_ 16.4 and δ_P2_ 7.0 (d, *J* = 26.9 Hz); ^13^C {^1^H} NMR (126 MHz, CDCl_3_) δ 133.0 (bs), 129.2 (d, *J* = 2.9 Hz), 128.6 (d, *J* = 2.2 Hz), 127.7 (d, *J* = 5.8 Hz), 73.2 (dd, *J*_1_ = 172.5 Hz, *J*_2_ = 6.5 Hz), 63.62 (d, *J* = 7.0 Hz), 63.60 (d, *J* = 6.6 Hz), 62.1 (d, *J* = 6.4 Hz) 16.34 (d, *J* = 5.8 Hz), 16.1 (d, *J* = 6.5 Hz); ^1^H NMR (500 MHz, CDCl_3_) δ 7.48–7.31 (m, “a”), 6.81 (d, 1H, *J* = 715.2 Hz), 5.60 (dd, 1H, *J*_1_ = *J*_2_ = 11.2 Hz), 4.24–3.83 (m, “b”), 1.31–1.15 (m, “c”); HRMS (*m*/*z*): [M + Na]^+^ Calcd for C_11_H_18_O_6_P_2_Na 331.0476; Found: 331.0472.

“a”: total int.: 10H; “b”: total int.: 10H; “c”: total int.: 15H.

##### Diethyl α-Ethoxyphosphoryloxy-3,5-di-*tert*-butylbenzylphosphonate (**7c**) and Diethyl α-Hydroxyphosphoryloxy-3,5-di-*tert*-butylbenzylphosphonate (**8c**)

Yield: 0.24 g (55%), colorless oil.

**7c** (75%): ^31^P {^1^H} NMR (122 MHz, CDCl_3_) δ_P1_ 17.0 and δ_P2_ 7.7 (d, *J* = 26.6 Hz); ^13^C {^1^H} NMR (75 MHz, CDCl_3_) δ 151.0 (d, *J* = 2.2 Hz), 132.0 (t, *J* = 2.1 Hz), 123.0 (d, *J* = 2.8 Hz), 122.07 (d, *J* = 5.8 Hz), 73.6 (dd, *J*_*1*_ = 171.7 Hz, *J*_*2*_ = 7.0 Hz), 63.7 (d, *J* = 7.2 Hz), 61.9 (d, *J* = 6.6 Hz), 34.9 (s), 31.4 (s), 16.4 and 16.2 (d, *J* = 5.8 Hz), 16.0 (d, *J* = 6.5 Hz); ^1^H NMR (300 MHz, CDCl_3_) δ 7.41–7.39 and 7.33–7.30 (m, 3H), 6.97 (d, 1H, *J* = 726.8 Hz), 5.71 (dd, 1H, *J*_1_ = *J*_2_ = 11.7 Hz), 4.15–3.85 (m, 6H), 1.32 (s, 18H), 1.28 (t, 3H, *J* = 7.1 Hz), 1.20 and 1.17 (t, 6H, *J* = 7.3 Hz); HRMS (*m*/*z*): [M + Na]^+^ Calcd for C_21_H_38_O_6_P_2_Na 471.2041; Found: 471.2036.

**8c** (25%): ^31^P {^1^H} NMR (122 MHz, CDCl_3_) δ_P1_ 16.7 and δ_P2_ 7.1 (d, *J* = 27.5 Hz); ^13^C {^1^H} NMR (75 MHz, CDCl_3_) δ 151.1 (d, *J* = 2.2 Hz), 131.7 (bs), 123.1 (d, *J* = 2.7 Hz), 122.13 (d, *J* = 5.1 Hz), 73.9 (dd, *J*_*1*_ = 173.1 Hz, *J*_*2*_ = 6.4 Hz), 62.2 (d, *J* = 6.4 Hz), ^1^H NMR (300 MHz, CDCl_3_) δ 5.63 (dd, 1H, *J*_1_ = *J*_2_ = 11.1 Hz), and the other signals are common with those of the major isomer. HRMS (*m*/*z*): [M + Na]^+^ Calcd for C_19_H_34_O_6_P_2_Na 443.1728; Found 443.1734.

##### Diethyl α-Ethoxyphosphoryloxy-4-methylbenzylphosphonate (**7d**) and Diethyl α-Hydroxyphosphoryloxy-4-methylbenzylphosphonate (**8d**)

Yield: 0.20 g (59%), colorless oil.

**7d** (55%): ^31^P {^1^H} NMR (202 MHz, CDCl_3_) δ_P1_ 16.9 and δ_P2_ 7.7 (d, *J* = 26.6 Hz); ^13^C {^1^H} NMR (126 MHz, CDCl_3_) δ 139.1 (d, *J* = 2.9 Hz), 130.2 (bs), 129.26 (d, *J* = 2.1 Hz), 127.7 (d, *J* = 5.9 Hz), 72.9 (dd, *J*_1_ = 172.2 Hz, *J*_2_ = 6.9 Hz), 63.62 (d, *J* = 7.3 Hz), 61.8 (d, *J* = 6.4 Hz), 21.2 (s), 16.38 and 16.30 (d, *J* = 5.7 Hz), 16.1 (d, *J* = 6.6 Hz); ^1^H NMR (500 MHz, CDCl_3_) δ 7.40–7.38 and 7.20–7.18 (m, “a”), 6.94 (d, 1H, *J* = 723.0 Hz), 5.65 (dd, 1H, *J*_1_ = *J*_2_ = 11.7 Hz), 4.26–3.87 (m, “b”), 2.35 (s, “c”), 1.34–1.19 (m, “d”); HRMS (*m*/*z*): [M + Na]^+^ Calcd for C_14_H_24_O_6_P_2_Na 373.0946; Found: 373.0944.

**8d** (45%): ^31^P {^1^H} NMR (202 MHz, CDCl_3_) δ_P1_ 16.6 and δ_P2_ 7.0 (d, *J* = 28.1 Hz); ^13^C {^1^H} NMR (126 MHz, CDCl_3_) δ 139.2 (d, *J* = 3.0 Hz), 129.9 (bs), 129.32 (d, *J* = 2.2 Hz), 127.8 (d, *J* = 5.9 Hz), 73.2 (dd, *J*_1_ = 173.7 Hz, *J*_2_ = 6.5 Hz), 63.64 (d, *J* = 6.7 Hz), 62.1 (d, *J* = 6.4 Hz), 21.2 (s), 16.40 (d, *J* = 5.5 Hz), 16.2 (d, *J* = 6.5 Hz); ^1^H NMR (500 MHz, CDCl_3_) δ 7.40–7.38 and 7.20–7.18 (m, “a”), 6.82 (d, 1H, *J* = 714.5 Hz), 5.58 (dd, 1H, *J*_1_ = *J*_2_ = 11.1 Hz), 4.26–3.87 (m, “b”), 2.35 (s, “c”), 1.34–1.19 (m, “d”); HRMS (*m*/*z*) [M + Na]^+^ Calcd for C_12_H_20_O_6_P_2_Na 345.0633; Found: 345.0626.

“a”: total int.: 8H; “b”: total int.: 10H; “c”: total int.: 6H; “d”: total int.: 15H.

##### Diethyl α-Ethoxyphosphoryloxy-4-chlorobenzylphosphonate (**7e**) and Diethyl α-Hydroxyphosphoryloxy-4-chlorobenzylphosphonate (**8e**)

Yield: 0.24 g (66%), colorless oil.

**7e** (45%): ^31^P {^1^H} NMR (202 MHz, CDCl_3_) δ_P1_ 16.3 and δ_P2_ 7.9 (d, *J* = 24.8 Hz); ^13^C {^1^H} NMR (126 MHz, CDCl_3_) δ 135.1 (d, *J* = 3.2 Hz), 132.0 (bs), 129.0 (d, *J* = 5.6 Hz), 128.8 (d, *J* = 2.2 Hz), 72.3 (dd, *J*_1_ = 171.4 Hz, *J*_2_ = 6.6 Hz), 63.73 (d, *J* = 6.5 Hz), 61.9 (d, *J* = 6.3 Hz), 16.41 and 16.3 (d, *J* = 5.7 Hz), 16.1 (d, *J* = 6.4 Hz); ^1^H NMR (500 MHz, CDCl_3_) δ 7.46–7.32 (m, “a”), 6.98 (d, 1H, *J* = 724.5 Hz), 5.67 (dd, 1H, *J*_1_ = *J*_2_ = 11.3 Hz), 4.28–3.89 (m, “b”), 1.36–1.22 (m, “c”); HRMS (*m*/*z*): [M + Na]^+^ Calcd for C_13_H_21_ClO_6_P_2_Na 393.0400; Found 393.0393; [M + H]^+^ Calcd for C_13_H_22_ClO_6_P_2_ 371.0580; Found: 371.0577.

**8e** (55%): ^31^P {^1^H} NMR (202 MHz, CDCl_3_) δ_P1_ 16.0 and δ_P2_ 6.9 (d, *J* = 25.9 Hz); ^13^C {^1^H} NMR (126 MHz, CDCl_3_) δ 135.2 (d, *J* = 3.4 Hz), 131.8 (bs), 129.1 (d, *J* = 5.6 Hz), 128.9 (d, *J* = 2.2 Hz), 72.5 (dd, *J*_1_ = 172.7 Hz, *J*_2_ = 6.6 Hz), 63.71 (d, *J* = 6.6 Hz), 62.2 (d, *J* = 6.3 Hz), 16.40 (d, *J* = 5.6 Hz), 16.2 (d, *J* = 6.6 Hz); ^1^H NMR (500 MHz, CDCl_3_) δ 7.46–7.32 (m, “a”), 6.85 (d, 1H, *J* = 716.0 Hz,), 5.61 (dd, 1H, *J*_1_ = *J*_2_ = 11.3 Hz), 4.28–3.89 (m, “b”), 1.36–1.22 (m, “c”); HRMS (*m*/*z*): [M + Na]^+^ Calcd for C_11_H_17_ClO_6_P_2_Na 365.0087; Found: 365.0075.

“a”: total int.: 8H; “b”: total int.: 10H; “c”: total int.: 15H.

##### Diethyl α-Ethoxyphosphoryloxy-4-trifluoromethylbenzylphosphonate (**7f**) and Diethyl α-Hydroxyphosphoryloxy-4-trifluoromethylbenzylphosphonate (**8f**)

Yield: 0.27 g (69%), colorless oil.

**7f** (50%): ^31^P {^1^H} NMR (202 MHz, CDCl_3_) δ_P1_ 15.9 and δ_P2_ 8.0 (d, *J* = 23.4 Hz); ^13^C {^1^H} NMR (75 MHz, CDCl_3_) δ 137.3 (bs), 127.8 (d, *J* = 5.4 Hz), 125.6–125.4 (m), 123.8 (d, *J* = 272.3 Hz), 72.3 (dd, *J*_1_ = 169.4 Hz, *J*_2_ = 6.7 Hz), 63.86 (d, *J* = 6.8 Hz), 62.1 (d, *J* = 6.5 Hz), 16.37 and 16.30 (d, *J* = 4.8 Hz), 16.1 (d, *J* = 6.8 Hz); ^1^H NMR (500 MHz, CDCl_3_) δ 7.68–7.63 (m, “a”), 7.02 (d, 1H, *J* = 726.2 Hz), 5.78 (dd, 1H, *J*_1_ = 13.1 Hz, *J*_2_ = 11.3 Hz), 4.31–3.94 (m, “b”), 1.38–1.24 (m, “c”); HRMS (*m*/*z*): [M + Na]^+^ Calcd for C_14_H_21_F_3_O_6_P_2_Na 427.0663; Found: 427.0663; [M + H]^+^ Calcd for C_14_H_22_F_3_O_6_P_2_ 405.0844; Found: 405.0845; [M + H]^+^ Calcd for C_14_H_21_F_3_O_6_P_2_K 443.0403; Found: 443.0403.

**8f** (50%): ^31^P {^1^H} NMR (202 MHz, CDCl_3_) δ_P1_ 15.6 and δ_P2_ 6.9 (d, *J* = 24.6 Hz); ^13^C {^1^H} NMR (75 MHz, CDCl_3_) δ 137.5 (bs), 127.9 (d, *J* = 5.4 Hz), 125.6–125.4 (m), 123.8 (d, *J* = 272.3 Hz), 72.5 (dd, *J*_1_ = 170.9 Hz, *J*_2_ = 6.6 Hz), 63.82 (d, *J* = 6.8 Hz), 62.4 (d, *J* = 6.3 Hz) 16.30 (d, *J* = 5.0 Hz), 16.2 (d, *J* = 6.8 Hz); ^1^H NMR (500 MHz, CDCl_3_) δ 7.68–7.63 (m, “a”), 6.89 (d, 1H, *J* = 716.0 Hz,), 5.72 (dd, 1H, *J*_1_ = 14.0 Hz, *J*_2_ = 11.4 Hz,), 4.31–3.94 (m, “b”), 1.38–1.24 (m, “c”); HRMS (*m*/*z*): [M + Na]^+^ Calcd for C_12_H_17_F_3_O_6_P_2_Na 399.0350; Found: 399.0342.

“a”: total int.: 8H; (b) total int.: 10H; (c) total int.: 15H.

##### Diethyl α-Ethoxyphosphoryloxy-4-nitrobenzylphosphonate (**7g**) and Diethyl α-Hydroxyphosphoryloxy-4-nitrobenzylphosphonate (**8g**)

Yield: 0.24 g (64%), colorless oil.

**7g** (65%): ^31^P {^1^H} NMR (202 MHz, CDCl_3_) δ_P1_ 15.3 and δ_P2_ 8.2 (d, *J* = 21.9 Hz); ^13^C {^1^H} NMR (126 MHz, CDCl_3_) δ 148.1 (d, *J* = 3.3 Hz), 140.8 (bs), 128.2 (d, *J* = 5.1 Hz), 123.67 (d, *J* = 2.3 Hz), 72.1 (dd, *J*_1_ = 168.1 Hz, *J*_2_ = 6.6 Hz), 63.9 (d, *J* = 6.7 Hz), 62.2 (d, *J* = 6.3 Hz), 16.42 and 16.37 (d, *J* = 5.0 Hz), 16.2 (d, *J* = 6.7 Hz); ^1^H NMR (500 MHz, CDCl_3_) δ 8.29–8.23 and 7.71–7.66 (m, “a”), 7.06 (d, *J* = 727.8 Hz, “b”), 5.84 (dd, *J*_1_ = 13.9 Hz, *J*_2_ = 11.4 Hz, “c”), 4.32–3.98 (m, “d”), 1.40–1.27 (m, “e”); HRMS (*m*/*z*): [M + Na]^+^ Calcd for C_13_H_21_NO_8_P_2_Na 404.0640; Found: 404.0642; [M + H]^+^ Calcd for C_13_H_22_NO_8_P_2_ 382.0821; Found: 382.0822.

**8g** (35%): ^31^P {^1^H} NMR (202 MHz, CDCl_3_) δ_P1_ 15.0 and δ_P2_ 6.8 (d, *J* = 22.9 Hz); ^13^C {^1^H} NMR (126 MHz, CDCl_3_) δ 148.2 (d, *J* = 3.5 Hz), 140.6 (bs), 128.3 (d, *J* = 5.1 Hz), 123.71 (d, *J* = 2.3 Hz), 72.2 (dd, *J*_1_ = 169.6 Hz, *J*_2_ = 6.7 Hz), 64.0 (d, *J* = 7.2 Hz), 62.6 (d, *J* = 6.2 Hz), 16.39 (d, *J* = 5.5 Hz), 16.30 (d, *J* = 6.9 Hz); ^1^H NMR (500 MHz, CDCl_3_) δ 8.29–8.23 and 7.71–7.66 (m, “a”), 6.93 (d, “b”, *J* = 719.2 Hz), 5.79 (dd, “c”, *J*_1_ = 14.4 Hz, *J*_2_ = 11.4 Hz), 4.32–3.98 (m, “d”), 1.40–1.27 (m, “e”); HRMS (*m*/*z*): [M + Na]^+^ Calcd for C_11_H_17_NO_8_P_2_Na 376.0327; Found: 376.0331.

“a”: total int: 8H; “b”: total int: 2H; “c”: total int: 2H; “d”: total int: 10H; “e”: total int.: 15H.

### (α-Phosphinoyloxy-benzyl)-diphenylphosphine Oxide (**10**)

The mixture of 1.0 mmol (0.31 g) of diphenyl-(α-hydroxy-α-phenylmethyl)phosphine oxide and 2.0 mmol (0.40 g) of diphenylphosphine oxide in toluene (5 mL) was stirred at 110 °C under a N_2_ atmosphere. After a 24 h reaction time, the solvent was evaporated and the crude product was purified by reversed-phase HPLC column chromatography as above to afford 0.35 g (68%) of product **10** as a colorless oil.

^31^P {^1^H} NMR (202 MHz, CDCl_3_) δ_P1_ 35.7 and δ_P2_ 29.5 (d, *J* = 24.7 Hz); ^13^C {^1^H} NMR (75 MHz, CDCl_3_) δ 132.5–131.1 (m) and 129.3–127.8 (m), δ 74.8 (dd, *J*_*1*_ = 86.1 Hz, *J*_*2*_ = 7.6 Hz); ^1^H NMR (500 MHz, CDCl_3_) δ 8.02–7.99 and 7.62–7.01 (m, 25H), 6.33 (dd, 1H, *J*_*1*_ = 10.4 Hz, *J*_*2*_ = 2.3 Hz); HRMS (*m*/*z*): [M + Na]^+^ Calcd for C_31_H_26_O_3_P_2_Na 531.1255, Found: 531.1258.

#### Diethyl α-(Diphenylphosphinoyl)-benzylphosphonate (**13**)

The mixture of 1.0 mmol (0.31 g) of diethyl α-bromo-α-phenyl-methylphosphonate and 1.3 mmol (0.28 mL) of ethyldiphenylphosphinite in toluene (1.5 mL) was heated at 135 °C in a sealed tube under a N_2_ atmosphere. After a 24 h reaction time, the solvent was evaporated and the crude product was purified by reversed-phase HPLC column chromatography as above to afford 0.21 g (48%) of product **13** as a colorless oil.

The reaction was also carried out on a 2-fold scale, reacting 2.0 mmol (0.62 g) of diethyl α-bromo-α-phenyl-methylphosphonate and 2.6 mmol (0.56 mL) of ethyldiphenylphosphinite in toluene (3 mL). In this case, the yield of compound **13** was 60% (0.51 g).

^31^P {^1^H} NMR (122 MHz, CDCl_3_) δ_P1_ 26.6 and δ_P2_ 18.8 (d, *J* = 5.1 Hz); ^13^C {^1^H} NMR (126 MHz, CDCl_3_) δ 132.6 (d, *J* = 101.7 Hz), 131.7 and 131.3 (d, *J* = 2.8 Hz), 131.6 and 131.09 (d, *J* = 9.0 Hz), 131.14 (d, *J* = 3.9 Hz), 130.0 (bs), 128.4 (d, *J* = 3.1 Hz), 128.3 and 128.1 (d, *J* = 12.3 Hz), 127.5 (bs), 63.2 and 62.8 (d, *J* = 7.1 Hz), 48.2 (dd, *J*_*1*_ = 131.2 Hz, *J*_*2*_ = 58.0 Hz), 16.06 and 16.02 (d, *J* = 6.2 Hz); ^1^H NMR (500 MHz, CDCl_3_) δ 8.05–8.01, 7.60–7.45 and 7.34–7.15 (m, 15H), 4.25 (dd, 1H, *J*_*1*_ = 24.9 Hz, *J*_*2*_ = 12.1 Hz), 4.02–3.79 (m, 4H), 1.06 and 1.02 (t, 6H, *J* = 7.1 Hz); HRMS (*m*/*z*): [M + Na]^+^ Calcd for C_23_H_26_O_4_P_2_Na 451.1204; Found: 451.1204.

### Computational Method

DFT computations at the M062X/6-31+G (d,p) level of theory were performed considering the solvent effect of toluene using the SMD solvent model with the Gaussian 16 program package.^46−49^ The geometries of the molecules were optimized in all cases, and frequency calculations were also performed to ensure that the structures were in a local minimum or at a saddle point. The conformations of the reported structures were determined by a conformational analysis. The solution-phase enthalpies and Gibbs free energies were obtained by frequency calculations as well. The H and G values obtained were given under standard state; the corrected total energies of the molecules were taken into account. Entropic and thermal corrections were evaluated for isolated molecules using standard rigid rotor harmonic oscillator approximations; that is, the enthalpy and Gibbs free energy were taken as the “sum of electronic and thermal free energies” printed in Gaussian 16 vibrational frequency calculations. The standard state correction was also taken into account. The transition states were optimized with QST3 or with the TS (Berny) method. The transition states were identified by having one imaginary frequency in the Hessian matrix, and IRC calculations were performed to prove that the transition states connected two corresponding minima. For the details of the calculations, see the Supporting Information.

## Data Availability

The data underlying this study are available in the published article and its Supporting Information.

## Supporting Information Available

^31^P, ^13^C, and ^1^H NMR spectra; data of the theoretical calculations (PDF)
