# Supplementary material for: Unexpected Reaction of Dialkyl α-Hydroxy-benzylphosphonates with Dialkyl Phosphites and a Few Related Reactions
Source: J Org Chem. 2024 Dec 17;90(1):439–47. doi: 10.1021/acs.joc.4c02355 (PMC11731303; doi:10.1021/acs.joc.4c02355)
Supplement: Supplementary file 1 — jo4c02355_si_001.pdf [file jo4c02355_si_001.pdf]

Supporting Information

Unexpected Reaction of Dialkyl α-Hydroxy-Benzylphosphonates with Dialkyl Phosphites and a Few Related Reactions

Zsuzsanna Szalai,[a] Péter Ábrányi-Balogh,[a,b,c] and György Keglevich[a,*]

[a]*Department of Organic Chemistry and Technology, Faculty of Chemical Technology and Biotechnology, Budapest University of Technology and Economics, 1111 Budapest, Műegyetem rkp. 3. Hungary.*

keglevich.gyorgy@vbk.bme.hu.

[b]*Medicinal Chemistry Research Group, HUN-REN Research Centre for Natural Sciences, 1117 Budapest, Hungary.*

[c]*National Drug Research and Development Laboratory, HUN-REN Research Centre for Natural Sciences, 1117 Budapest, Hungary.*

Table of Contents

1. Spectra of the compounds **1c**, **7a-g**, **8a-g**, **10** and **13** synthesized....................................S2
2. Theoretical calculations..S37

1. Spectra of the compounds 1c, 7a-g, 8a-g, 10 and 13 synthesized

^{31}P {^{1}H} NMR (202 MHz, $CDCl_3$)

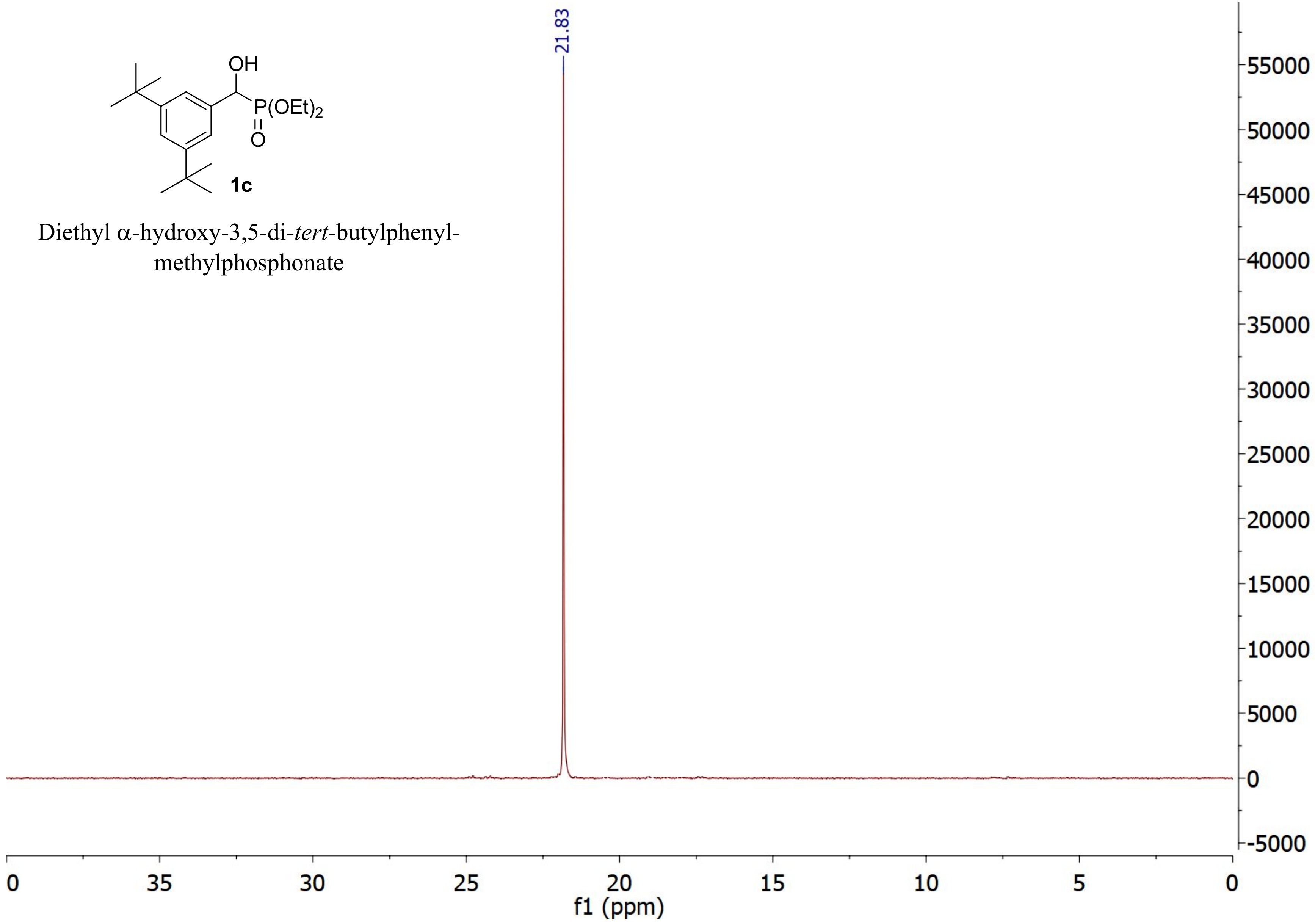

1c

Diethyl α-hydroxy-3,5-di-*tert*-butylphenyl-
methylphosphonate

$^{13}C\ \{^{1}H\}$ NMR (126 MHz, $CDCl_3$)

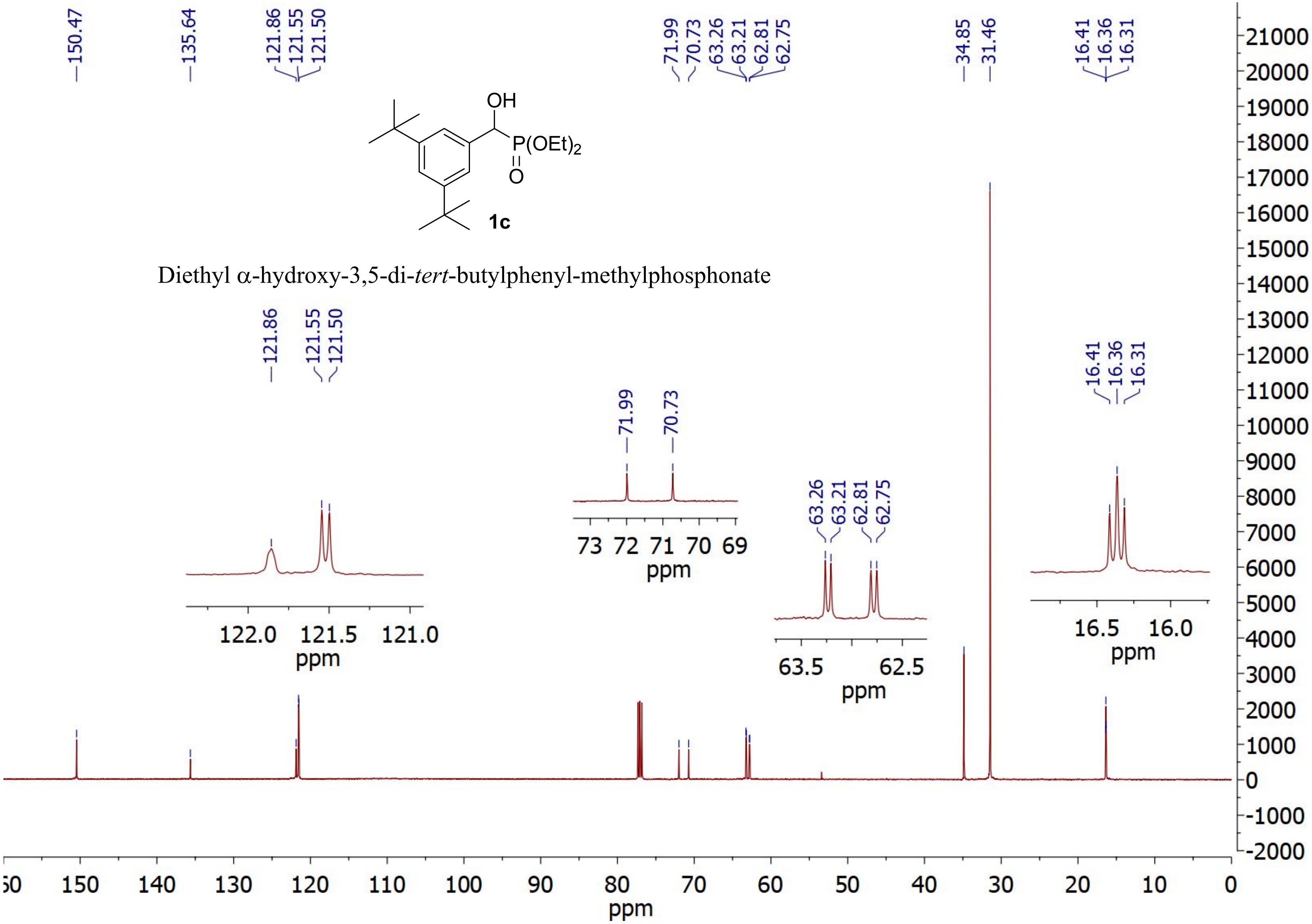

1c

Diethyl α-hydroxy-3,5-di-*tert*-butylphenyl-methylphosphonate

^{1}H NMR (500 MHz, $CDCl_3$)

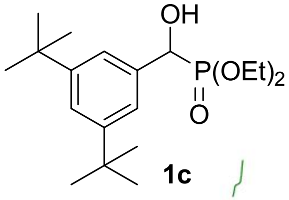

1c

Diethyl α-hydroxy-3,5-di-*tert*-butylphenyl-methylphosphonate

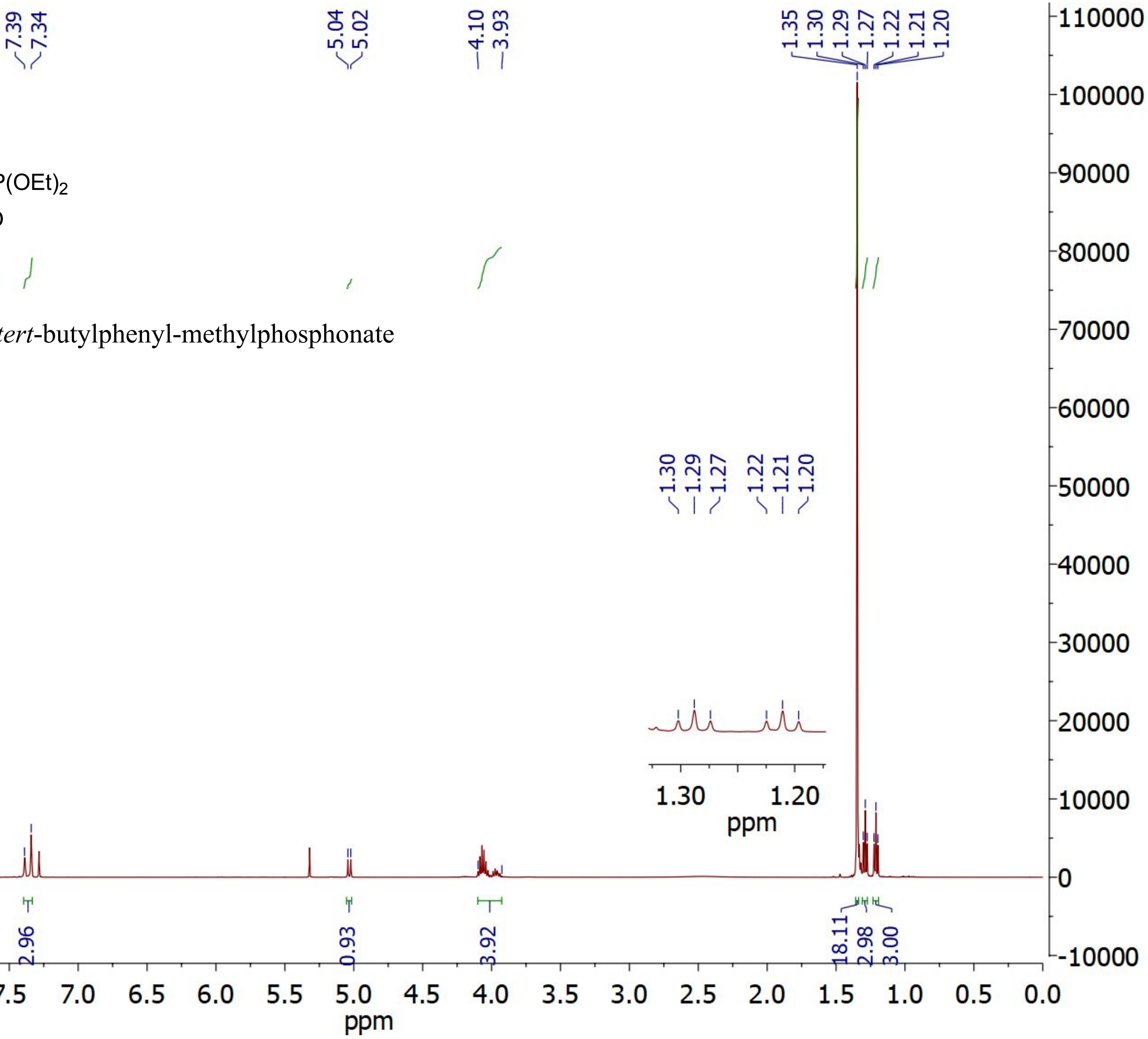

^{31}P {^{1}H} NMR (202 MHz, $CDCl_3$)

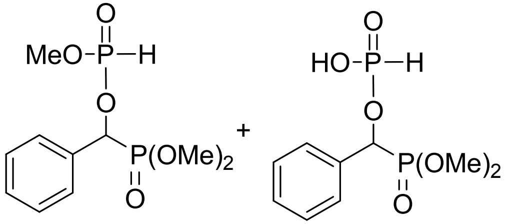

7a (25%) **8a** (75%)

Dimethyl α-methoxyphosphoryloxy-benzylphosphonate (**7a**) and
Dimethyl α-hydroxyphosphoryloxy-benzylphosphonate (**8a**)

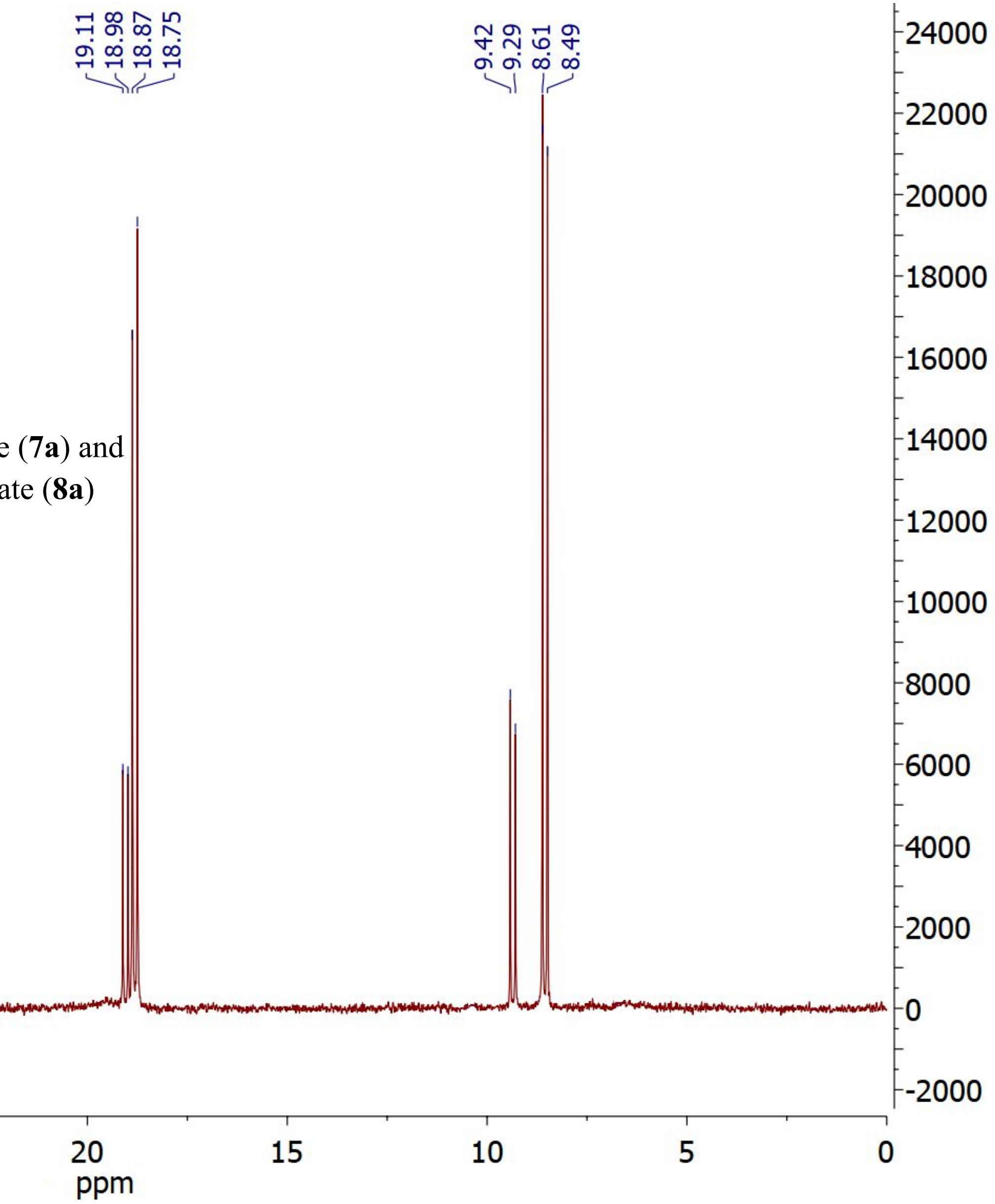

$^{13}C\ \{^{1}H\}$ NMR (75 MHz, $CDCl_3$)

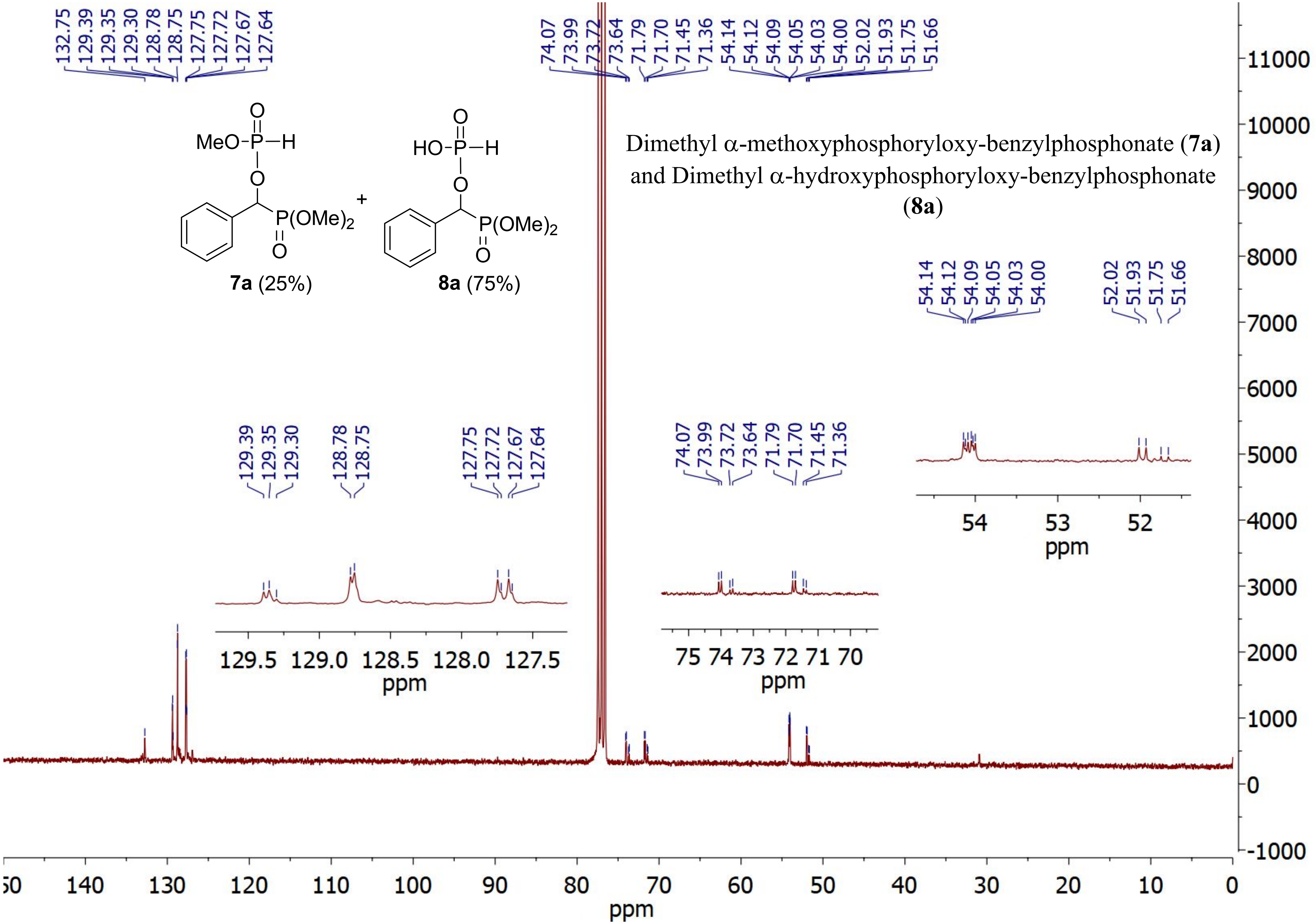

^{1}H NMR (500 MHz, $CDCl_3$)

Dimethyl α-methoxyphosphoryloxy-benzylphosphonate (**7a**) and
Dimethyl α-hydroxyphosphoryloxy-benzylphosphonate (**8a**)

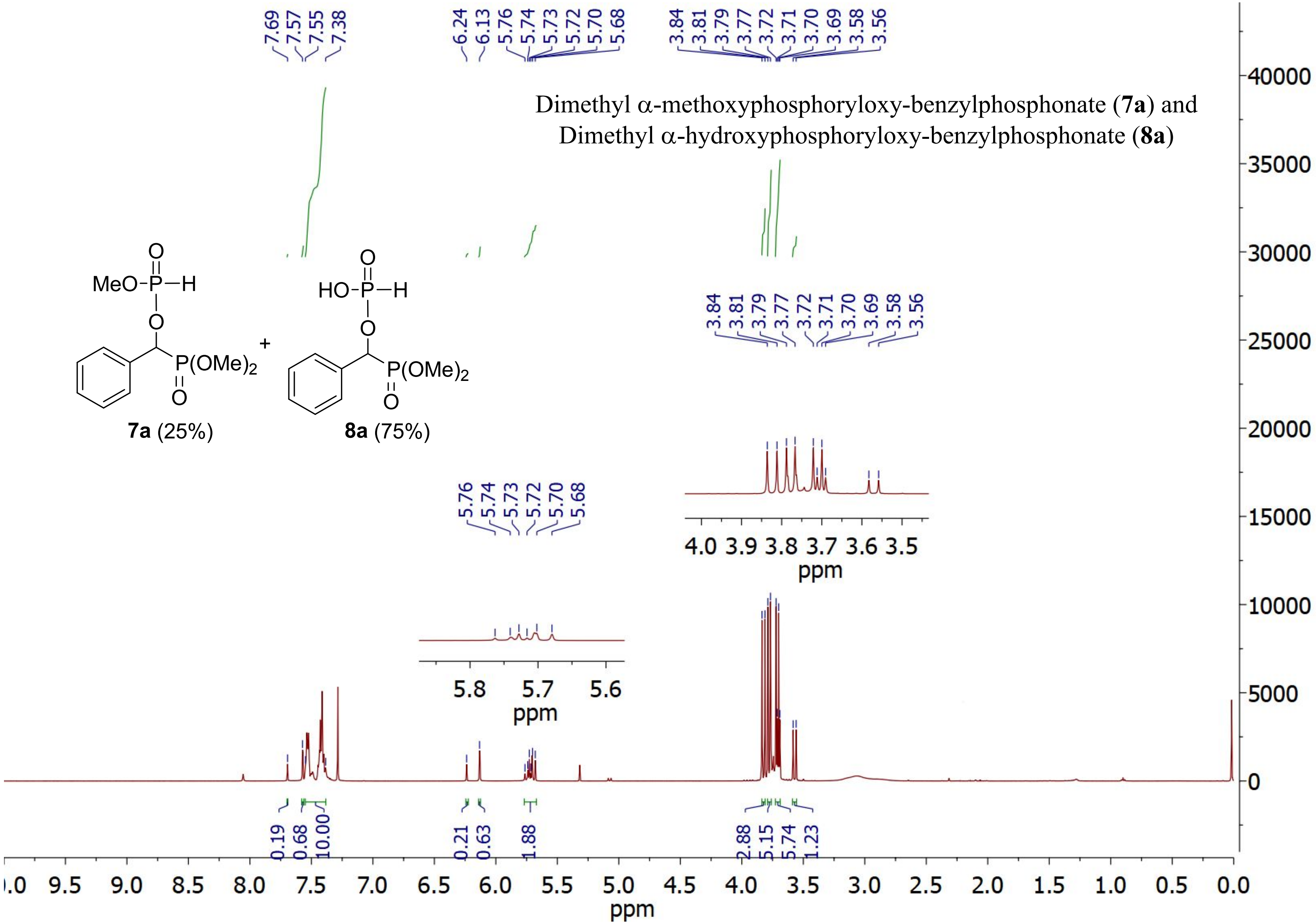

$^{31}P\ \{^{1}H\}$ NMR (202 MHz, $CDCl_3$)

7b

Diethyl α-ethoxyphosphoryloxy-benzylphosphonate

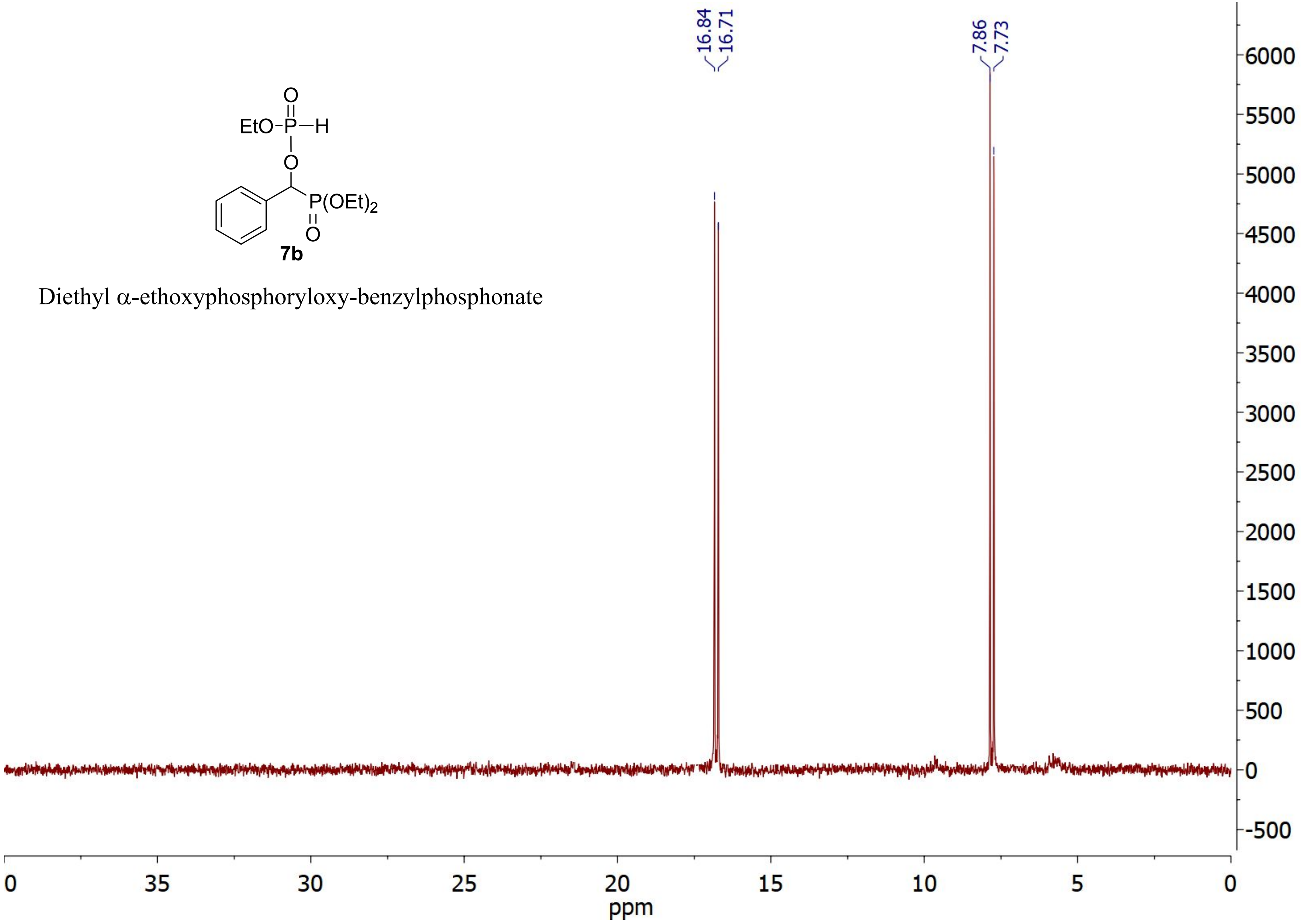

^{13}C {^{1}H} NMR (126 MHz, $CDCl_3$)

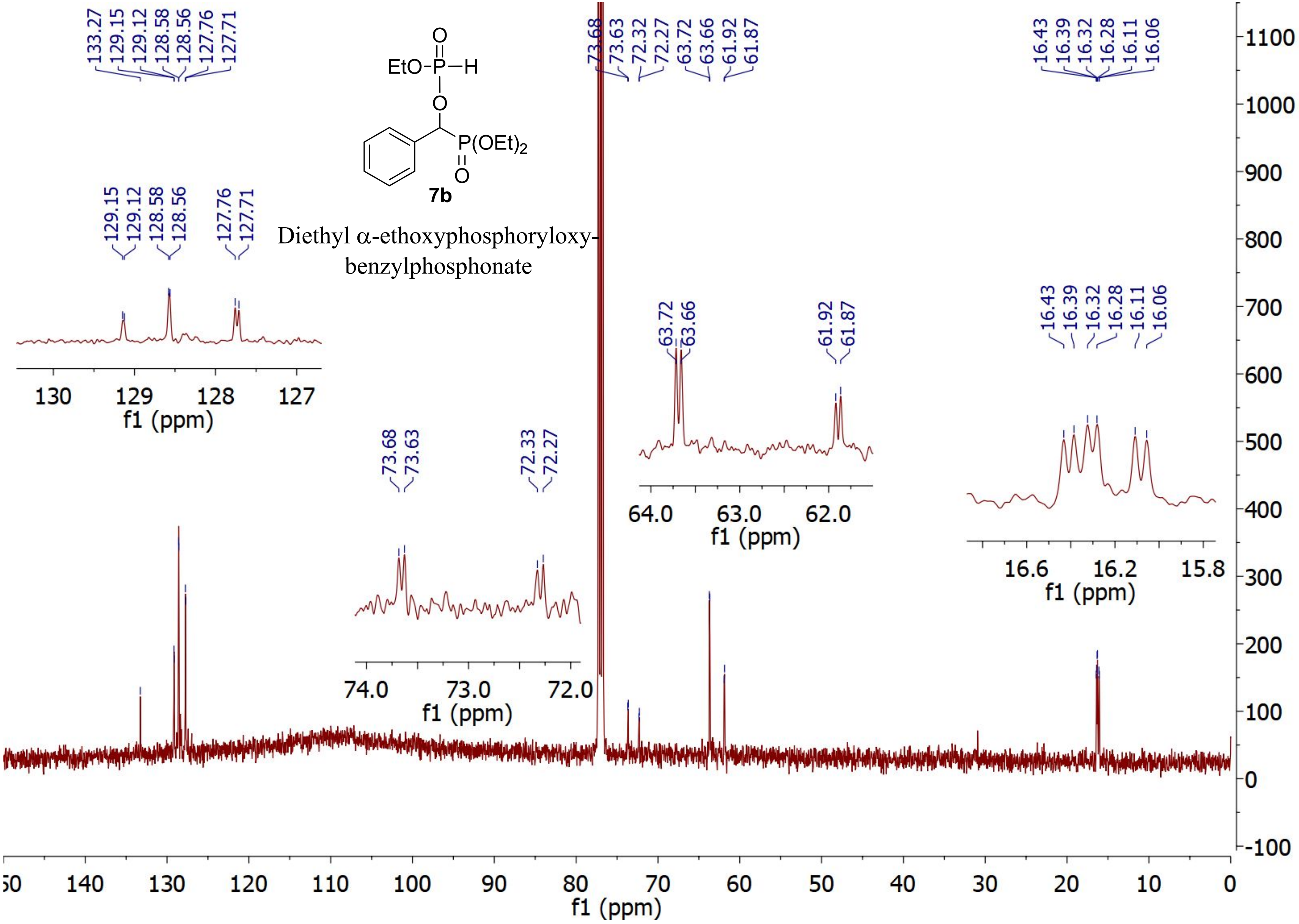

Diethyl α-ethoxyphosphoryloxy-benzylphosphonate

^{1}H NMR (500 MHz, $CDCl_3$)

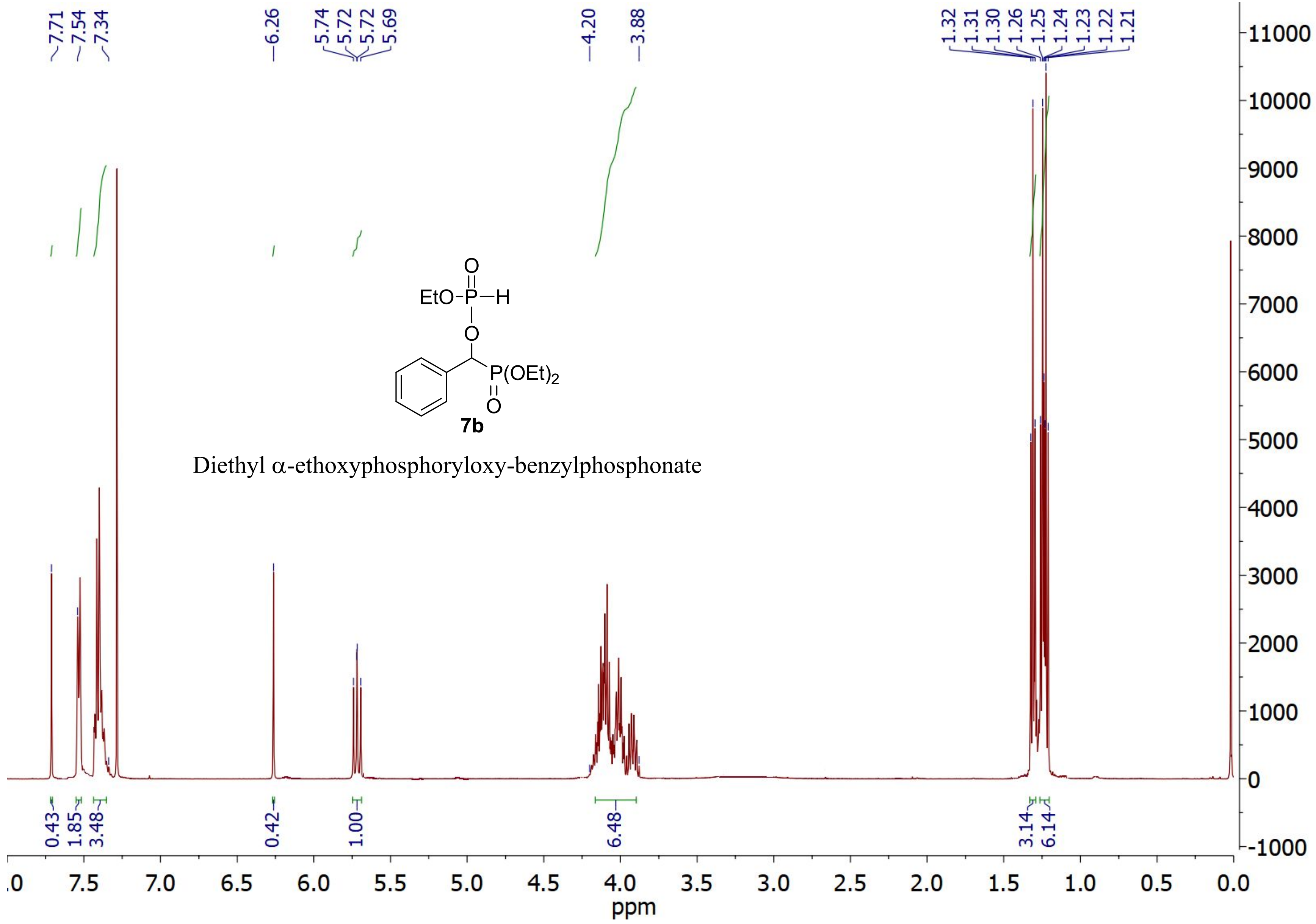

7b

Diethyl α-ethoxyphosphoryloxy-benzylphosphonate

^{31}P {^{1}H} NMR (202 MHz, $CDCl_3$)

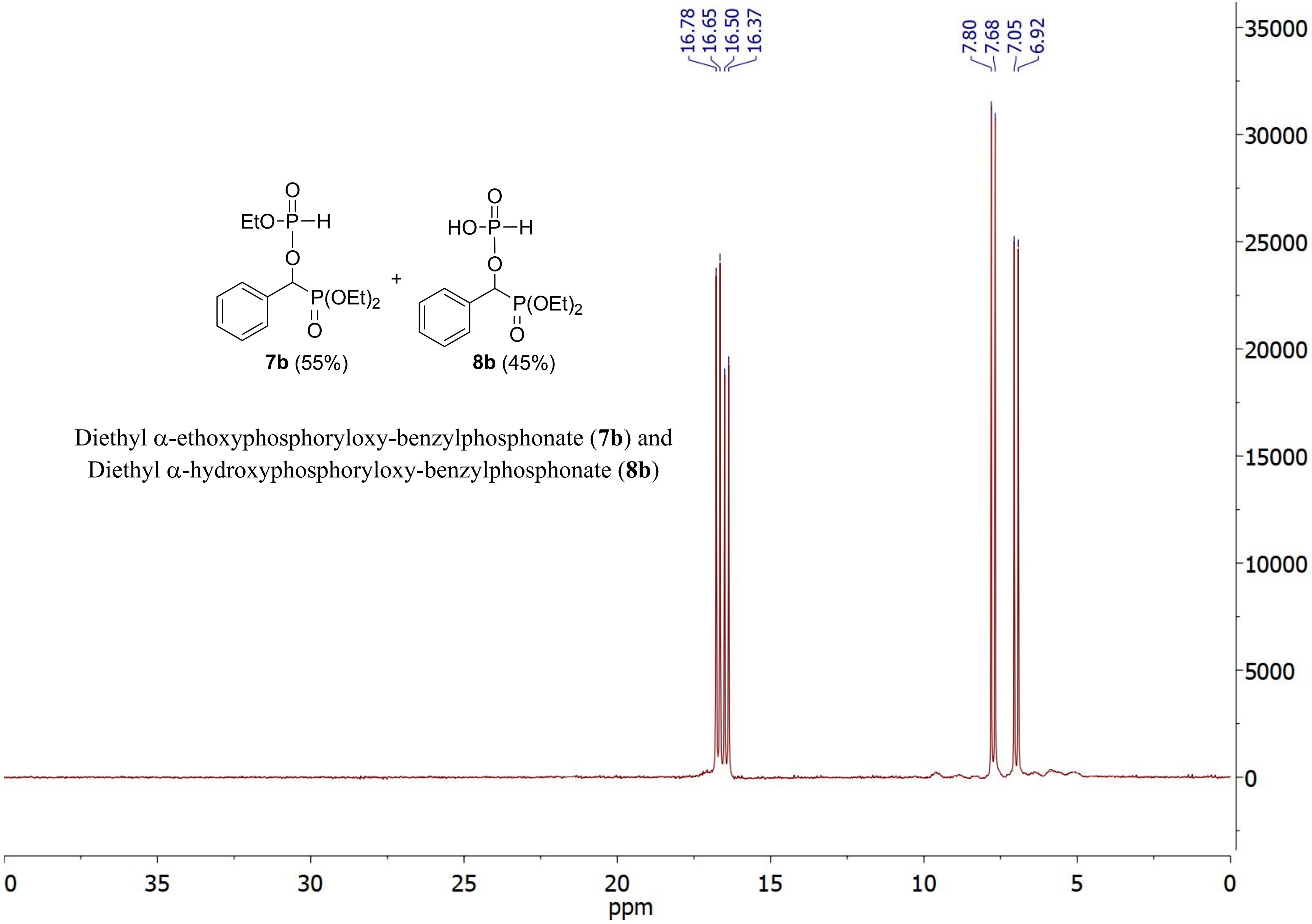

^{13}C {^{1}H} NMR (126 MHz, $CDCl_3$)

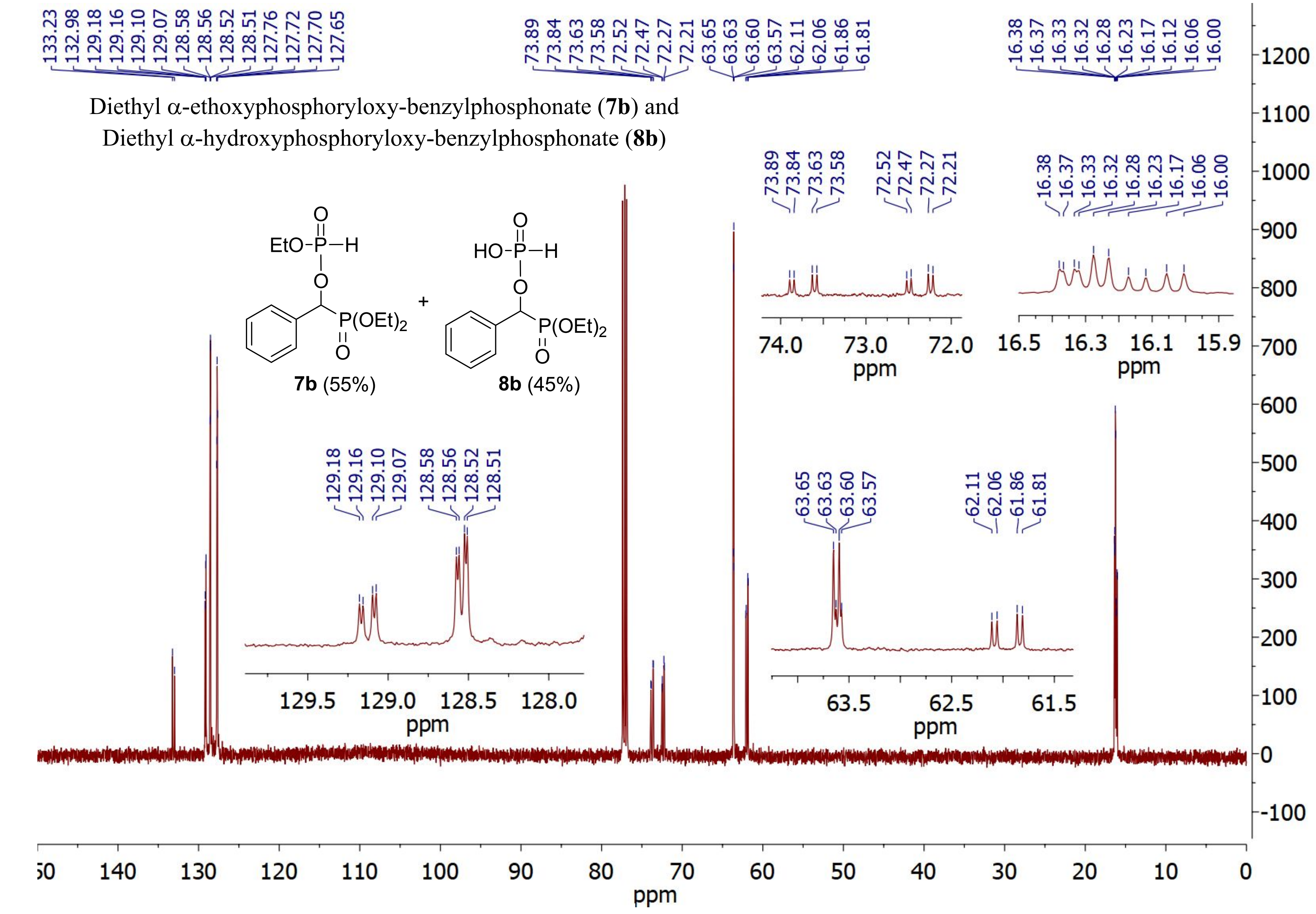

^{1}H NMR (500 MHz, CDCl$_3$)

Diethyl α-ethoxyphosphoryloxy-benzylphosphonate (**7b**) and
Diethyl α-hydroxyphosphoryloxy-benzylphosphonate (**8b**)

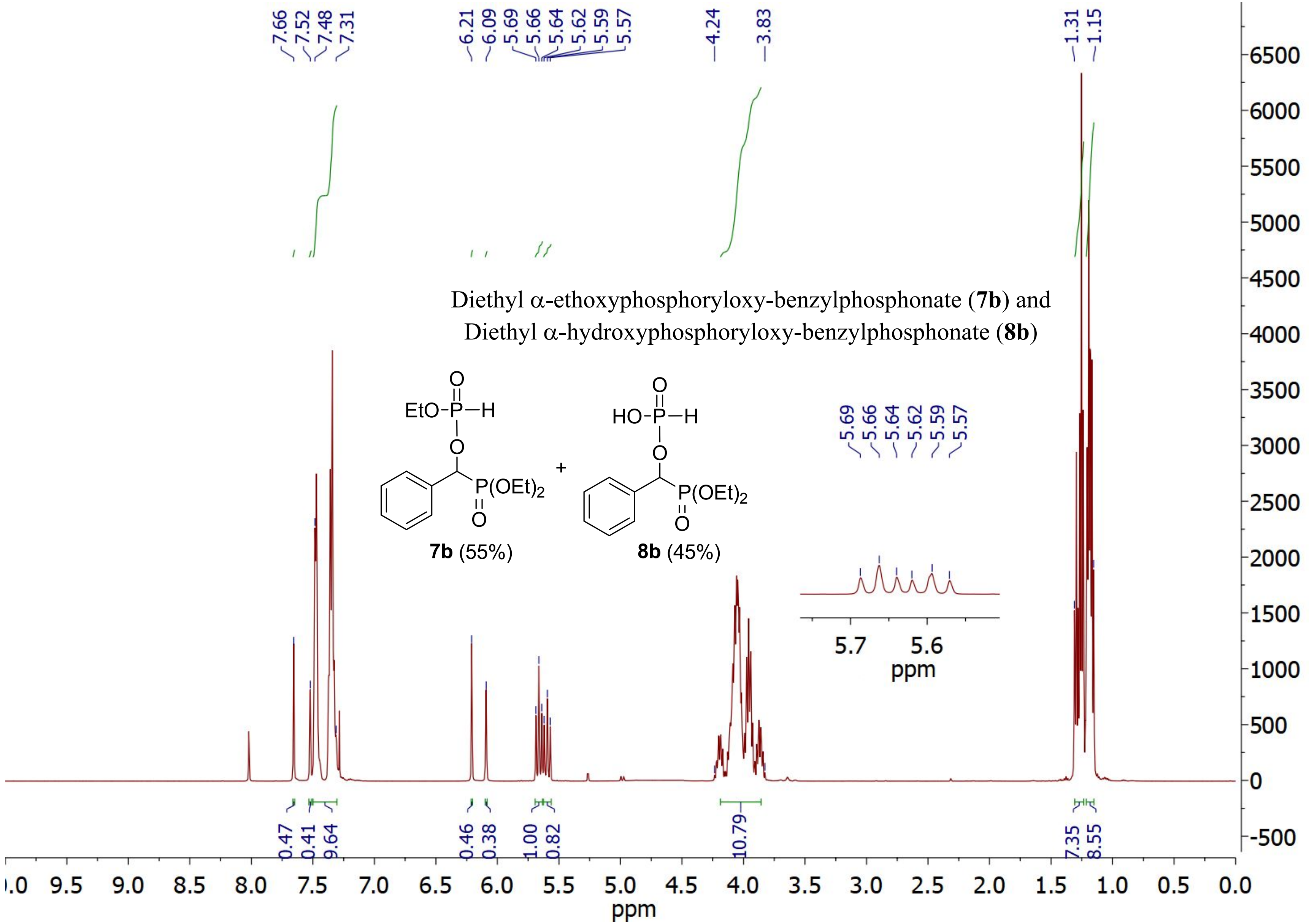

$^{31}P\ \{^{1}H\}$ NMR (122 MHz, $CDCl_3$)

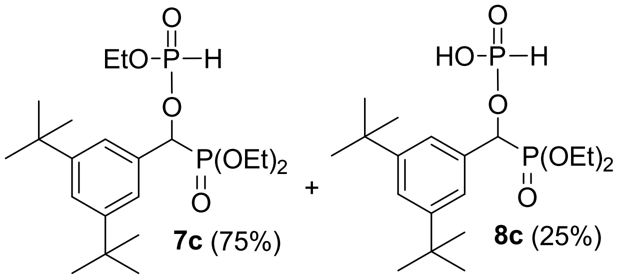

7c (75%) **8c** (25%)

Diethyl α-ethoxyphosphoryloxy-3,5-di-*tert*-butylbenzylphosphonate (**7c**) and diethyl α-hydroxyphosphoryloxy-3,5-di-*tert*-butylbenzylphosphonate (**8c**)

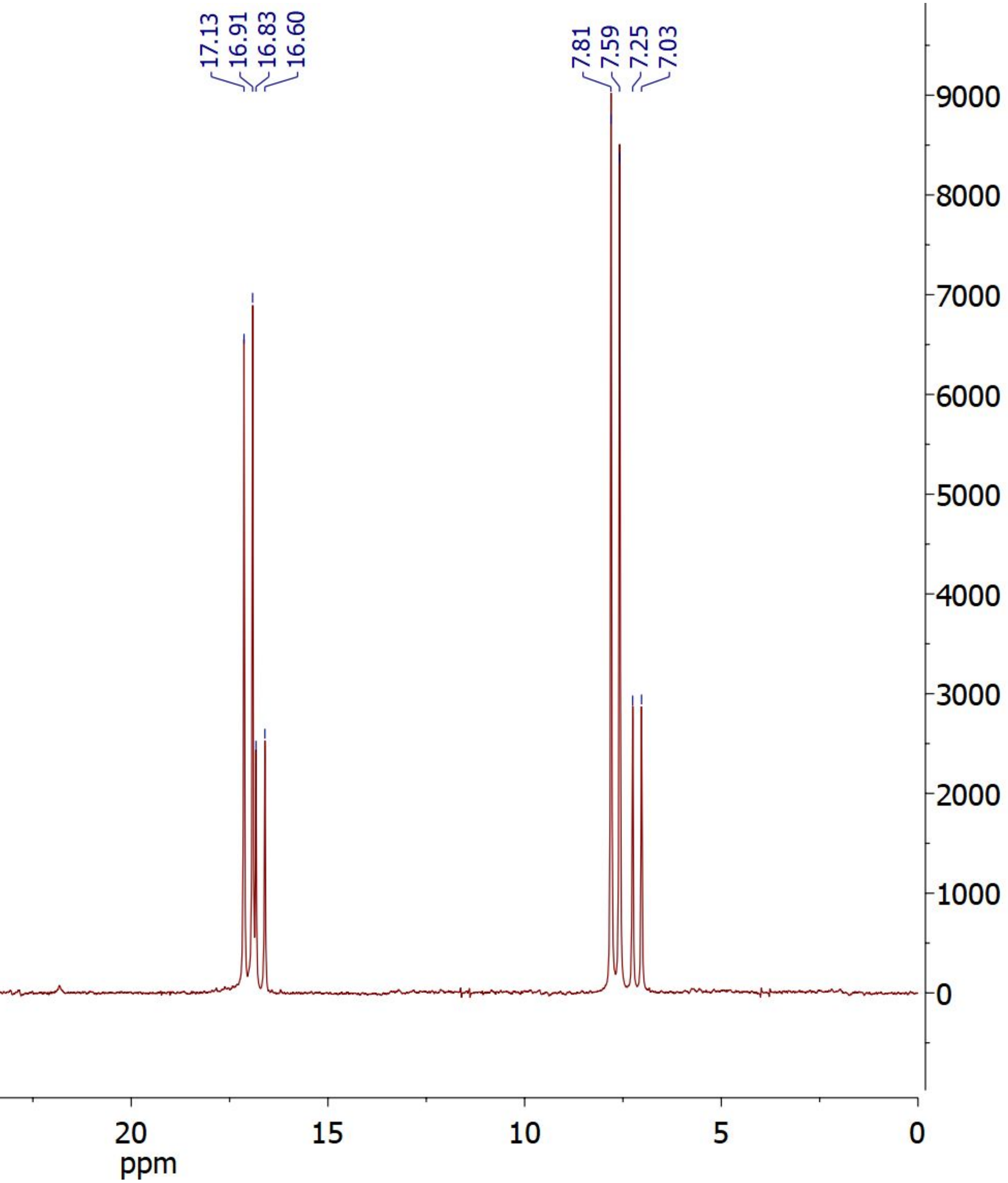

^{13}C {^{1}H} NMR (75 MHz, $CDCl_3$)

Diethyl α-ethoxyphosphoryloxy-3,5-di-*tert*-butylbenzylphosphonate (**7c**) and diethyl α-hydroxyphosphoryloxy-3,5-di-*tert*-butylbenzylphosphonate (**8c**)

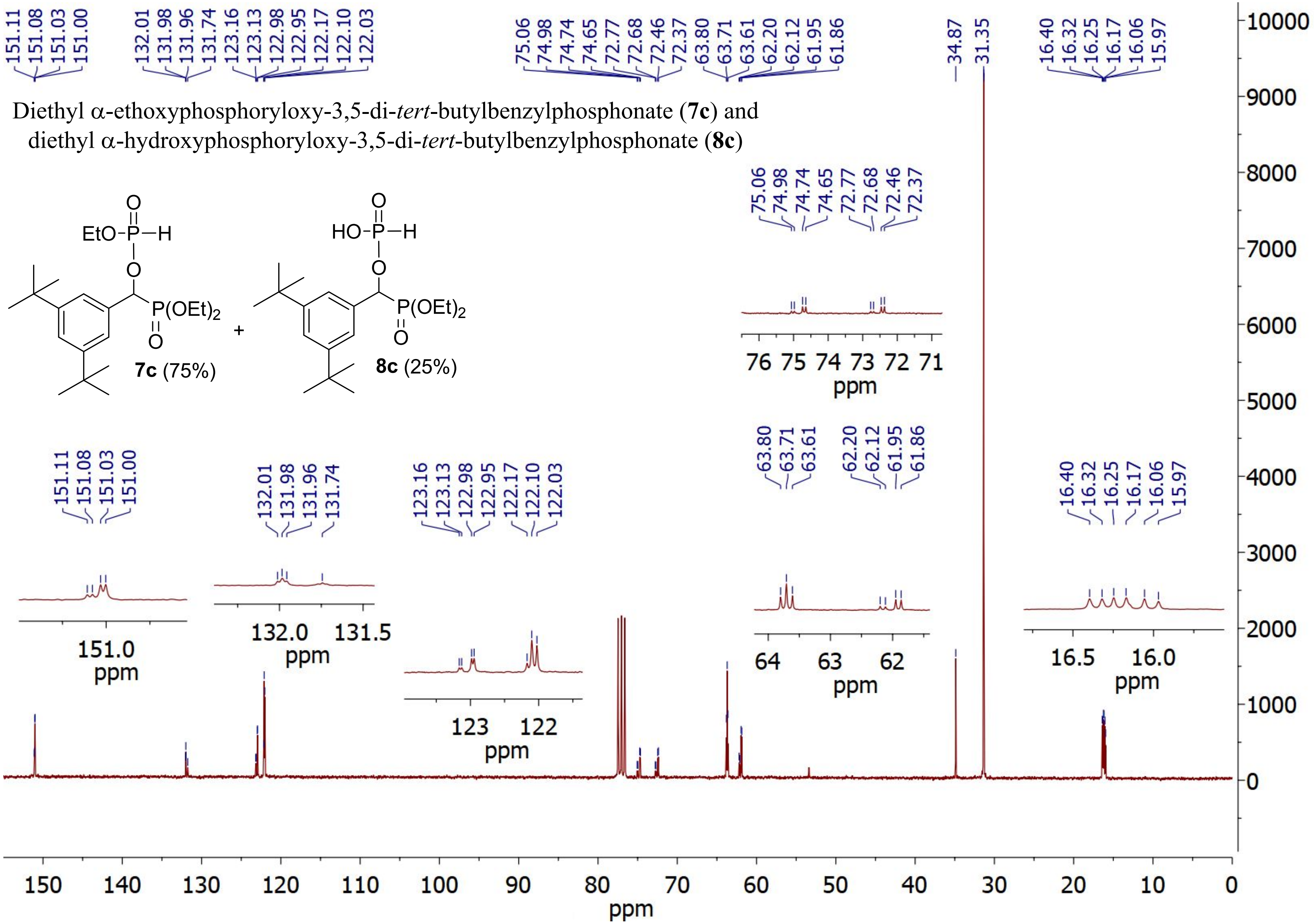

7c (75%) **8c** (25%)

^{1}H NMR (300 MHz, CDCl$_3$)

Diethyl α-ethoxyphosphoryloxy-3,5-di-*tert*-butylbenzylphosphonate (**7c**) and diethyl α-hydroxyphosphoryloxy-3,5-di-*tert*-butylbenzylphosphonate (**8c**)

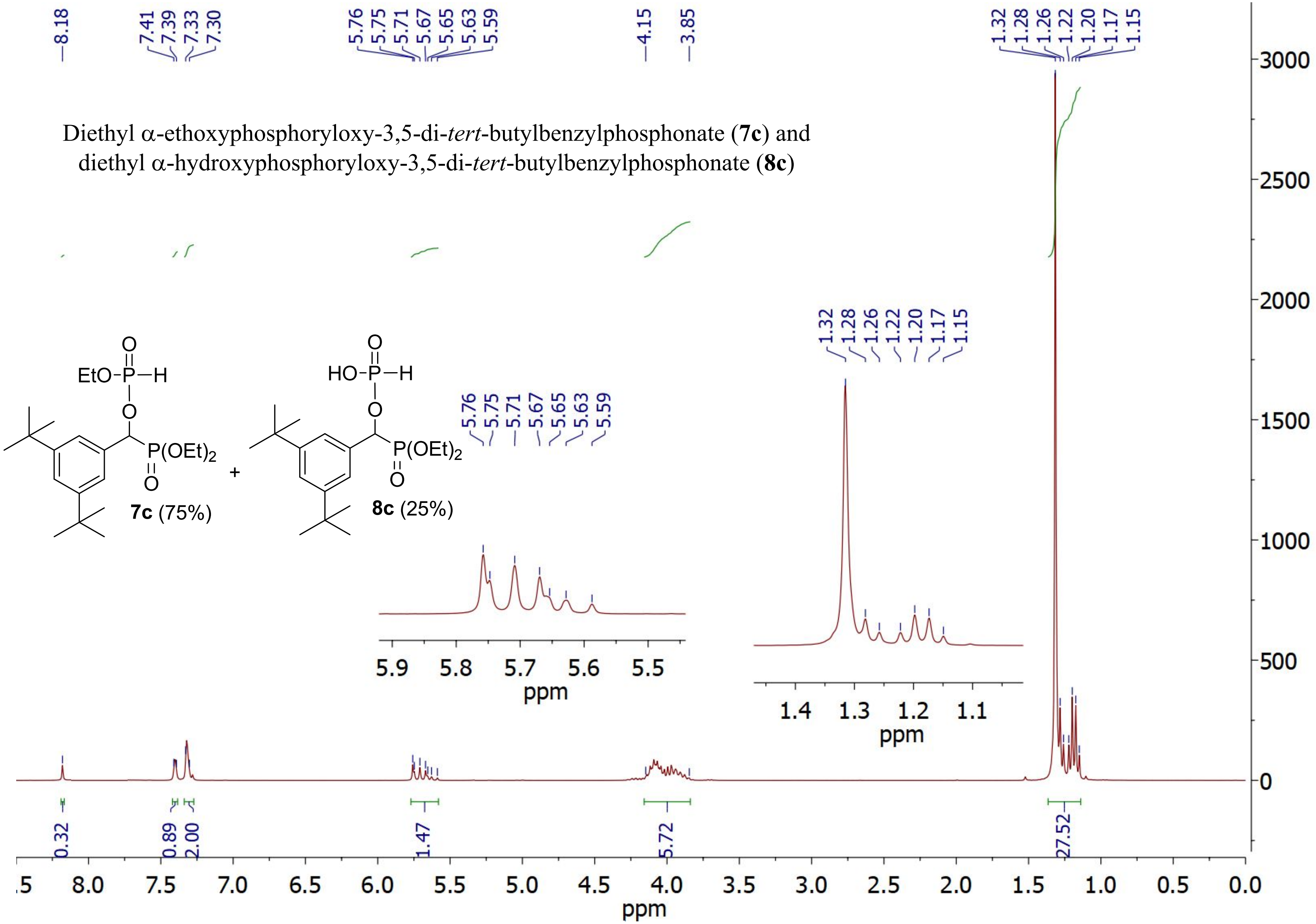

^{31}P {^{1}H} NMR (202 MHz, $CDCl_3$)

Diethyl α-ethoxyphosphoryloxy-4-methylbenzylphosphonate (**7d**) and diethyl α-hydroxyphosphoryloxy-4-methylbenzylphosphonate (**8d**)

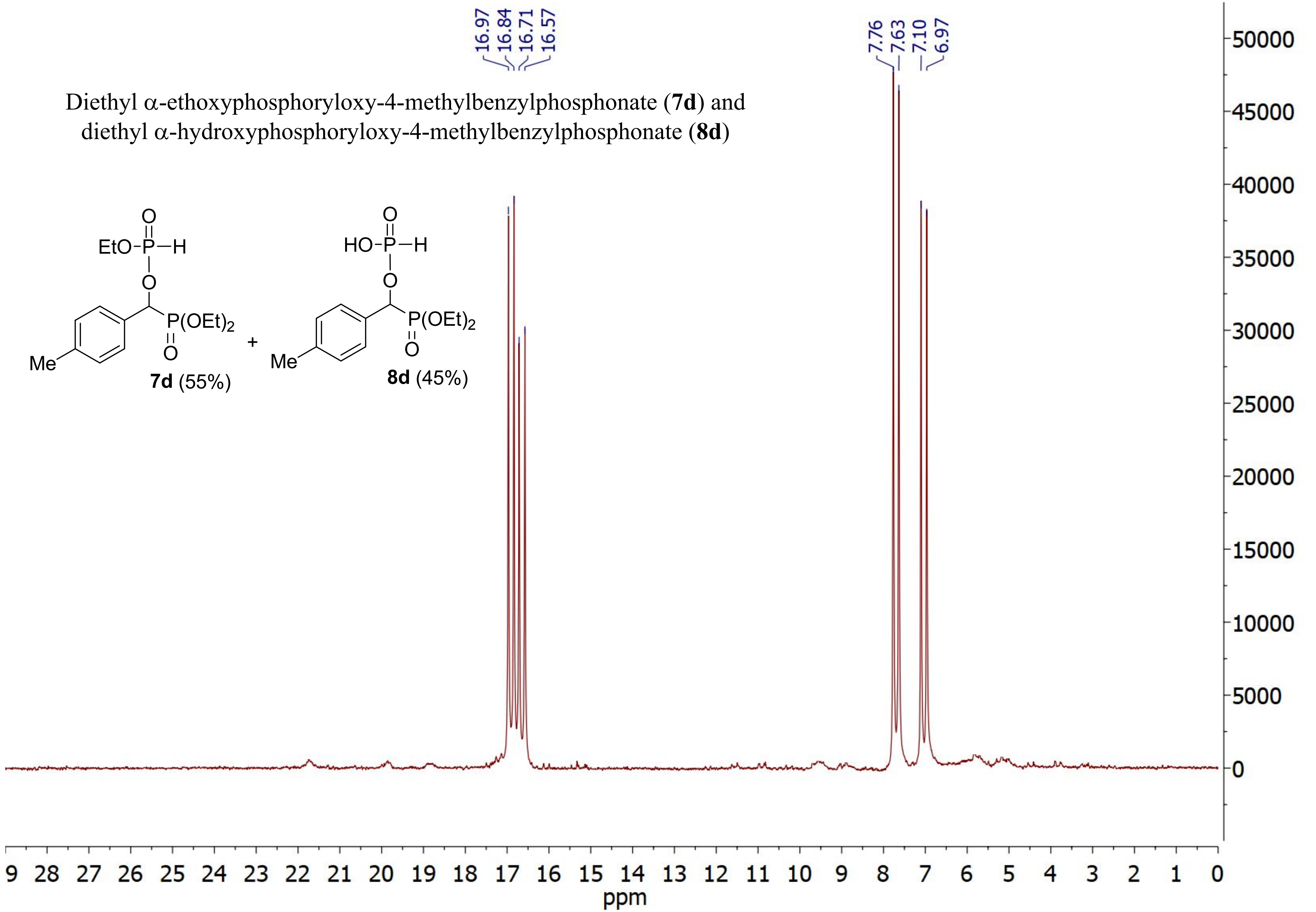

$^{13}C\ \{^1H\}$ NMR (126 MHz, $CDCl_3$)

Diethyl α-ethoxyphosphoryloxy-4-methylbenzylphosphonate (**7d**) and diethyl α-hydroxyphosphoryloxy-4-methylbenzylphosphonate (**8d**)

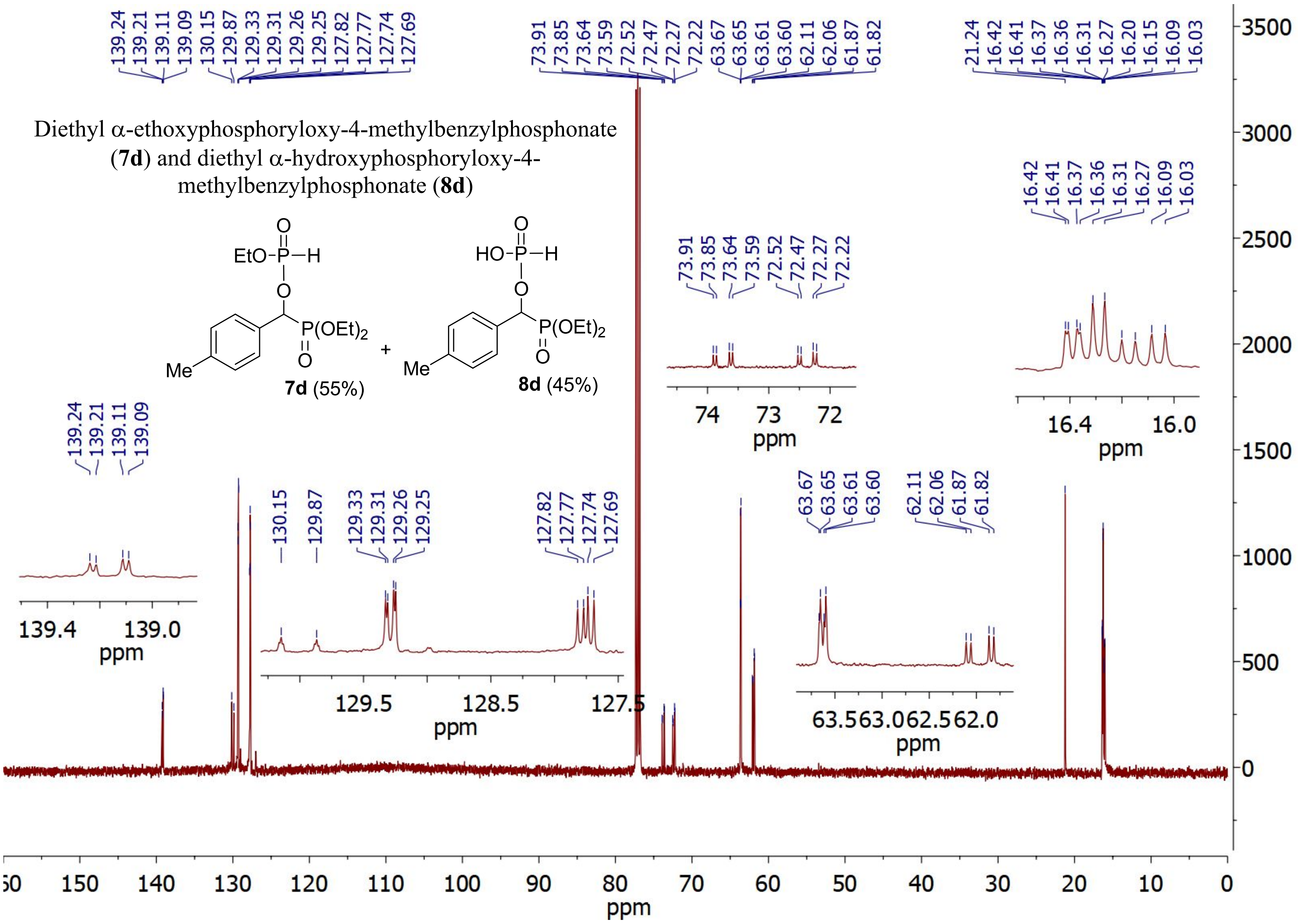

^{1}H NMR (500 MHz, $CDCl_3$)

Diethyl α-ethoxyphosphoryloxy-4-methylbenzylphosphonate (**7d**) and diethyl α-hydroxyphosphoryloxy-4-methylbenzylphosphonate (**8d**)

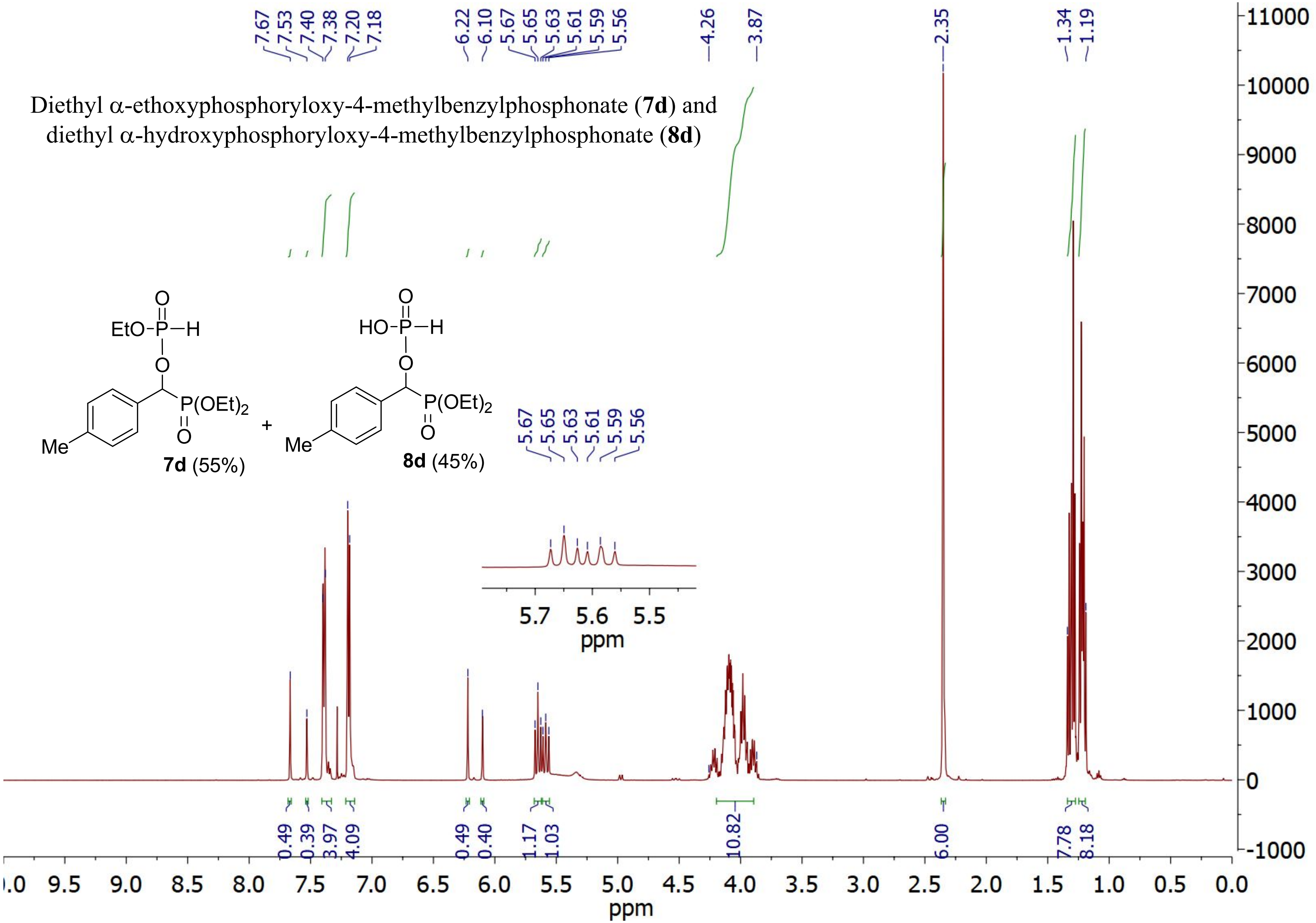

^{31}P {^{1}H} NMR (202 MHz, $CDCl_3$)

Diethyl α-ethoxyphosphoryloxy-4-chlorobenzylphosphonate (**7e**) and diethyl α-hydroxyphosphoryloxy-4-chlorobenzylphosphonate (**8e**)

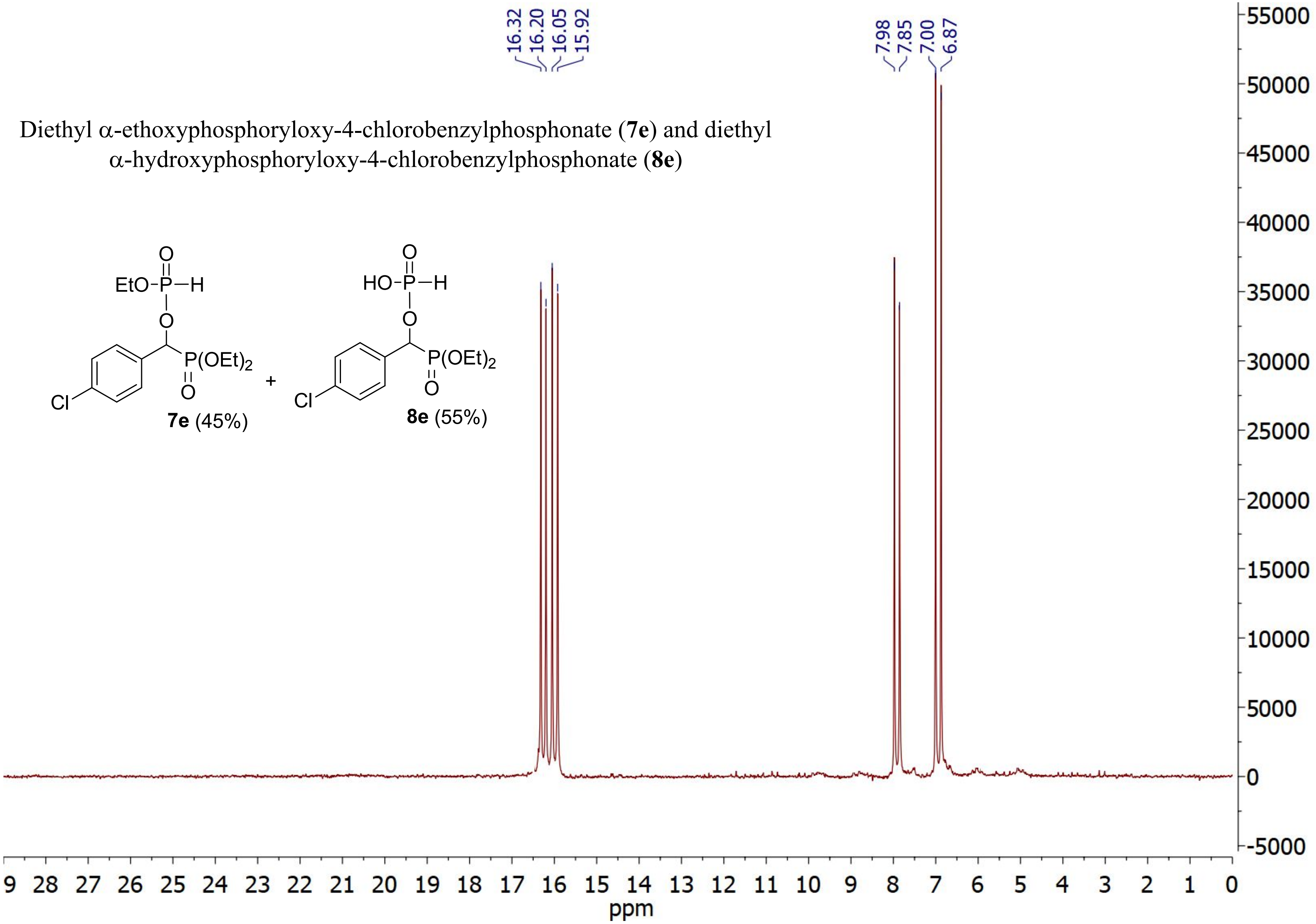

7e (45%) **8e** (55%)

^{13}C {^{1}H} NMR (126 MHz, $CDCl_3$)

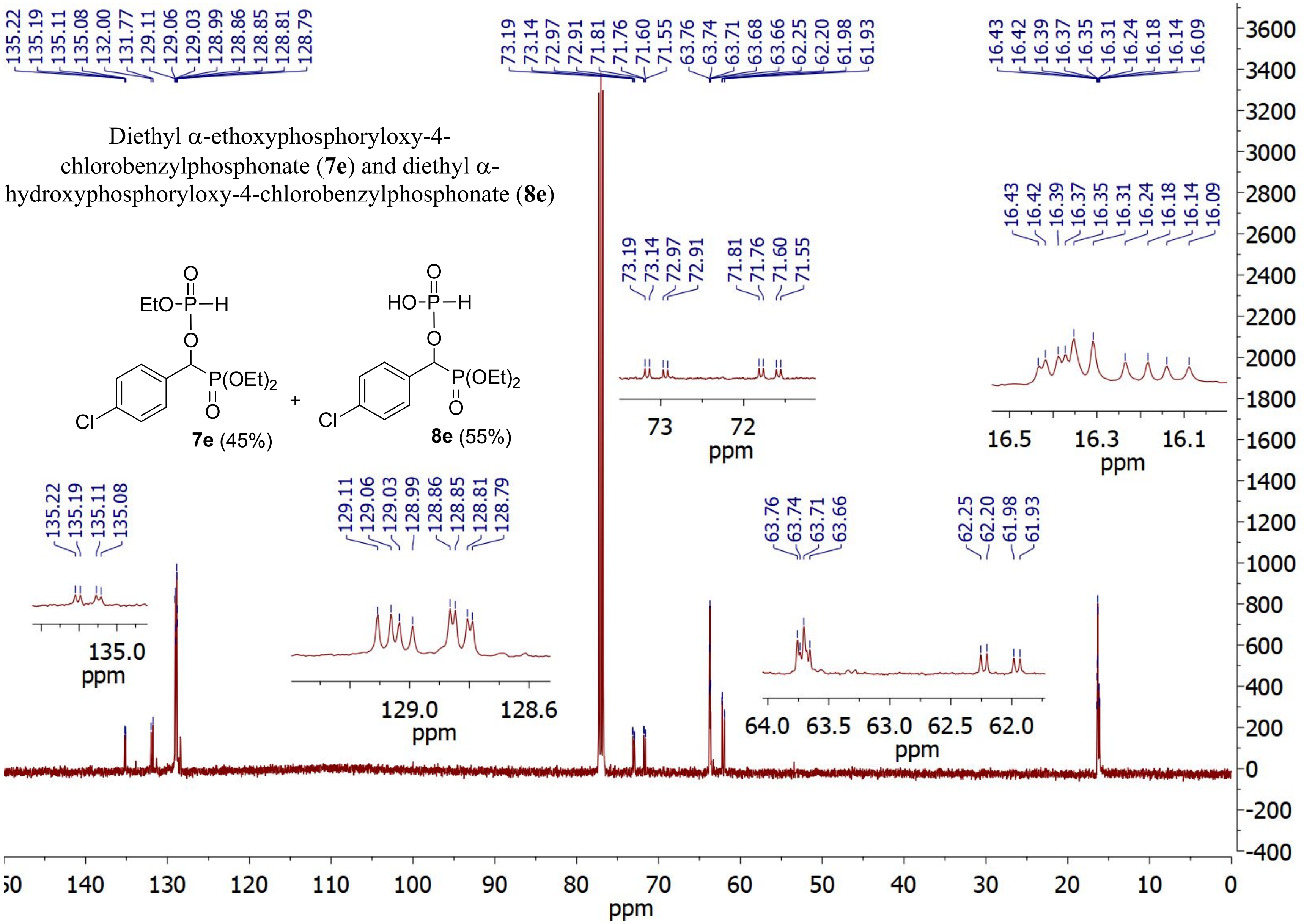

^{1}H NMR (500 MHz, $CDCl_3$)

Diethyl α-ethoxyphosphoryloxy-4-chlorobenzylphosphonate (**7e**) and diethyl α-hydroxyphosphoryloxy-4-chlorobenzylphosphonate (**8e**)

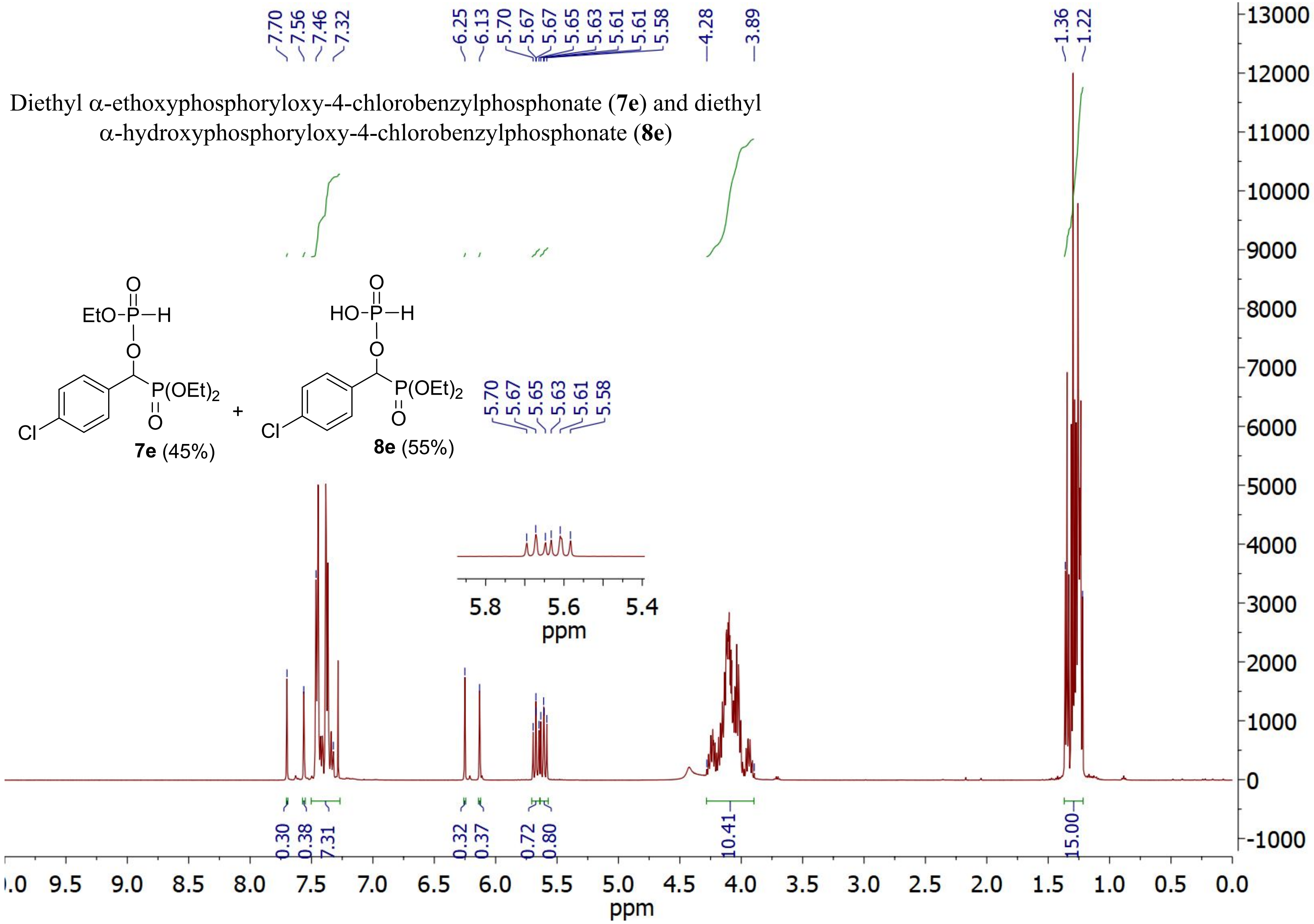

^{31}P {^{1}H} NMR (202 MHz, $CDCl_3$)

Diethyl α-ethoxyphosphoryloxy-4-trifluoromethylbenzylphosphonate (**7f**) and diethyl α-hydroxyphosphoryloxy-4-trifluoromethylbenzylphosphonate (**8f**)

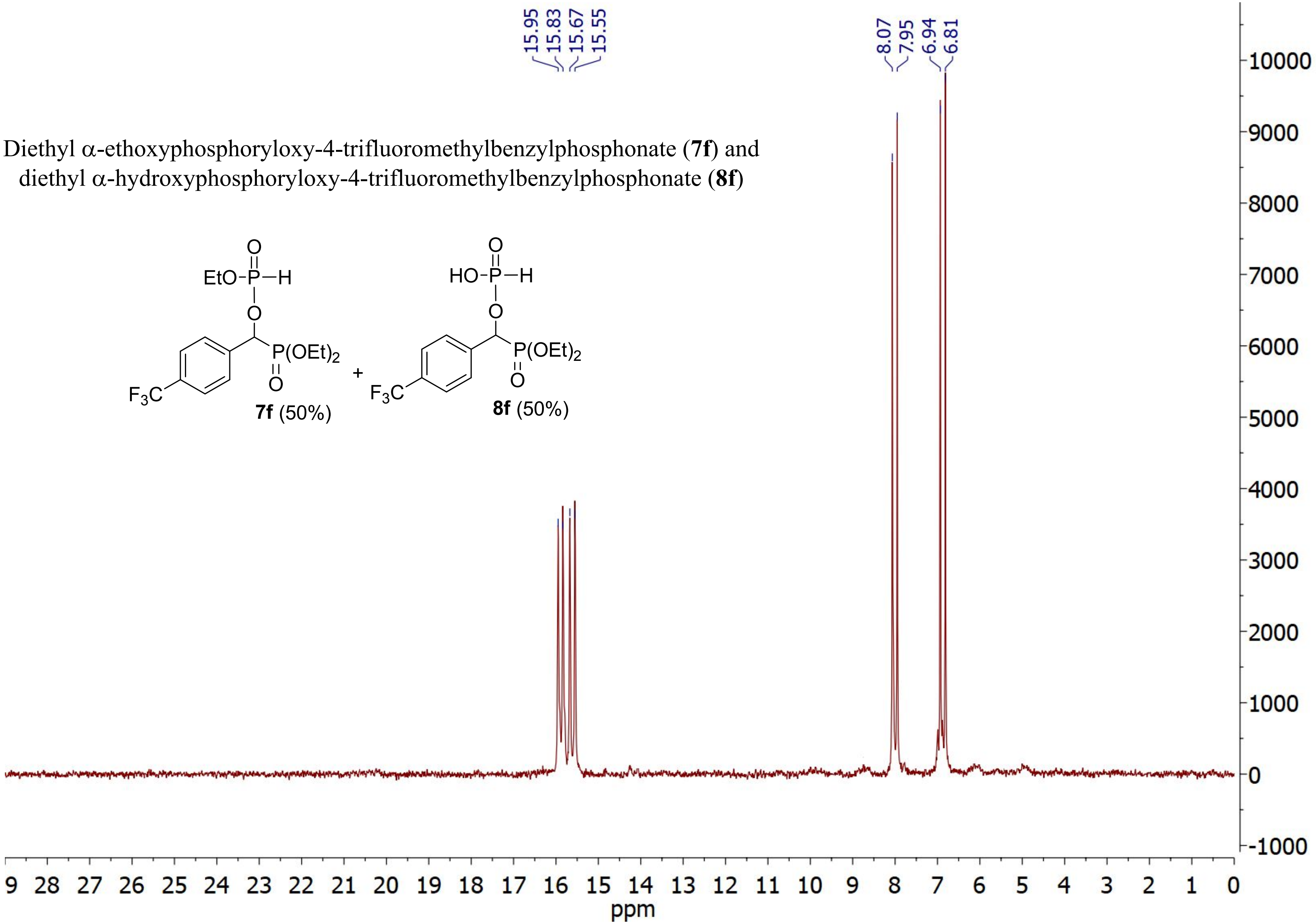

^{13}C {^{1}H} NMR (75 MHz, $CDCl_3$)

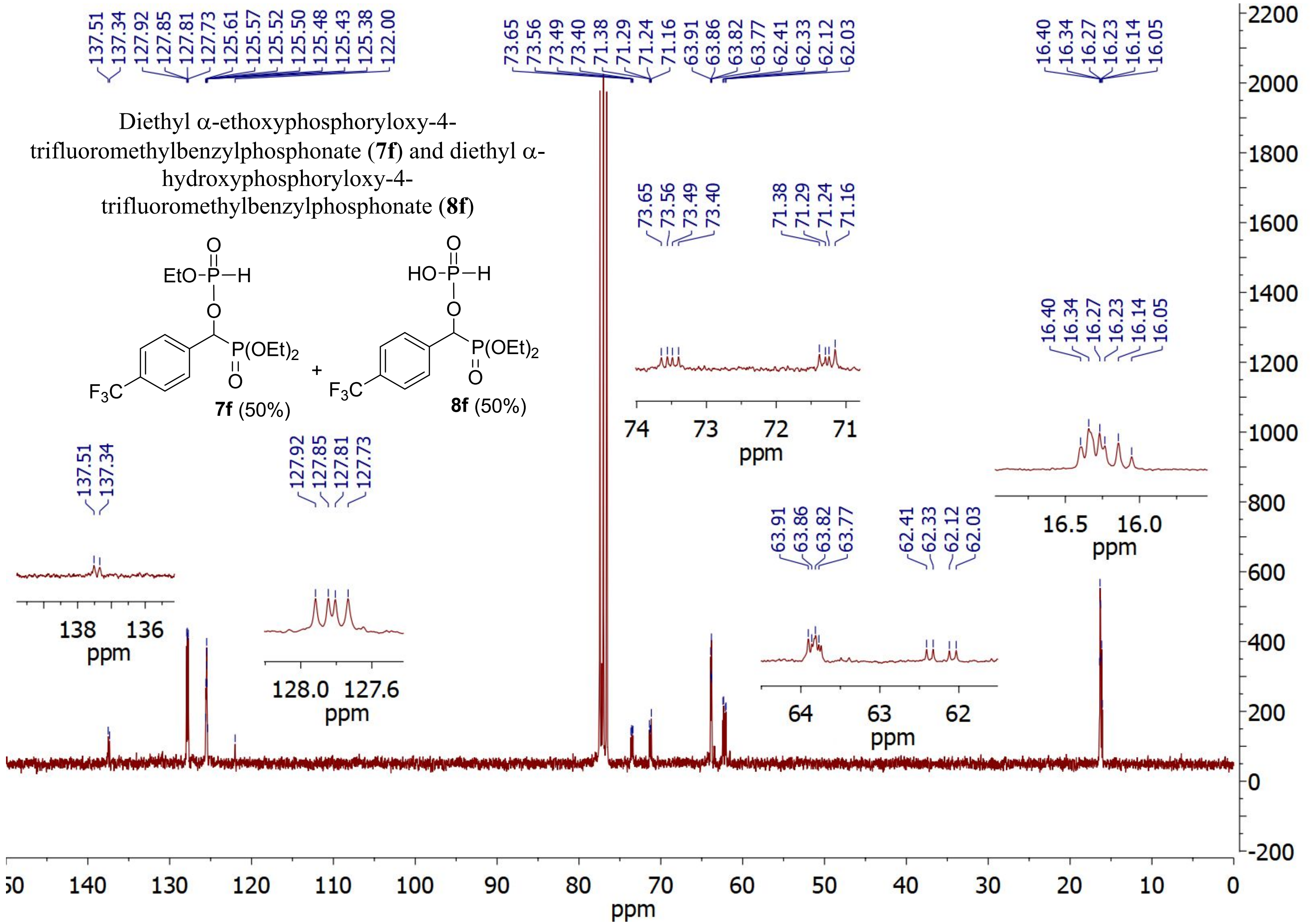

^{1}H NMR (500 MHz, CDCl$_3$)

Diethyl α-ethoxyphosphoryloxy-4-trifluoromethylbenzylphosphonate (**7f**) and diethyl α-hydroxyphosphoryloxy-4-trifluoromethylbenzylphosphonate (**8f**)

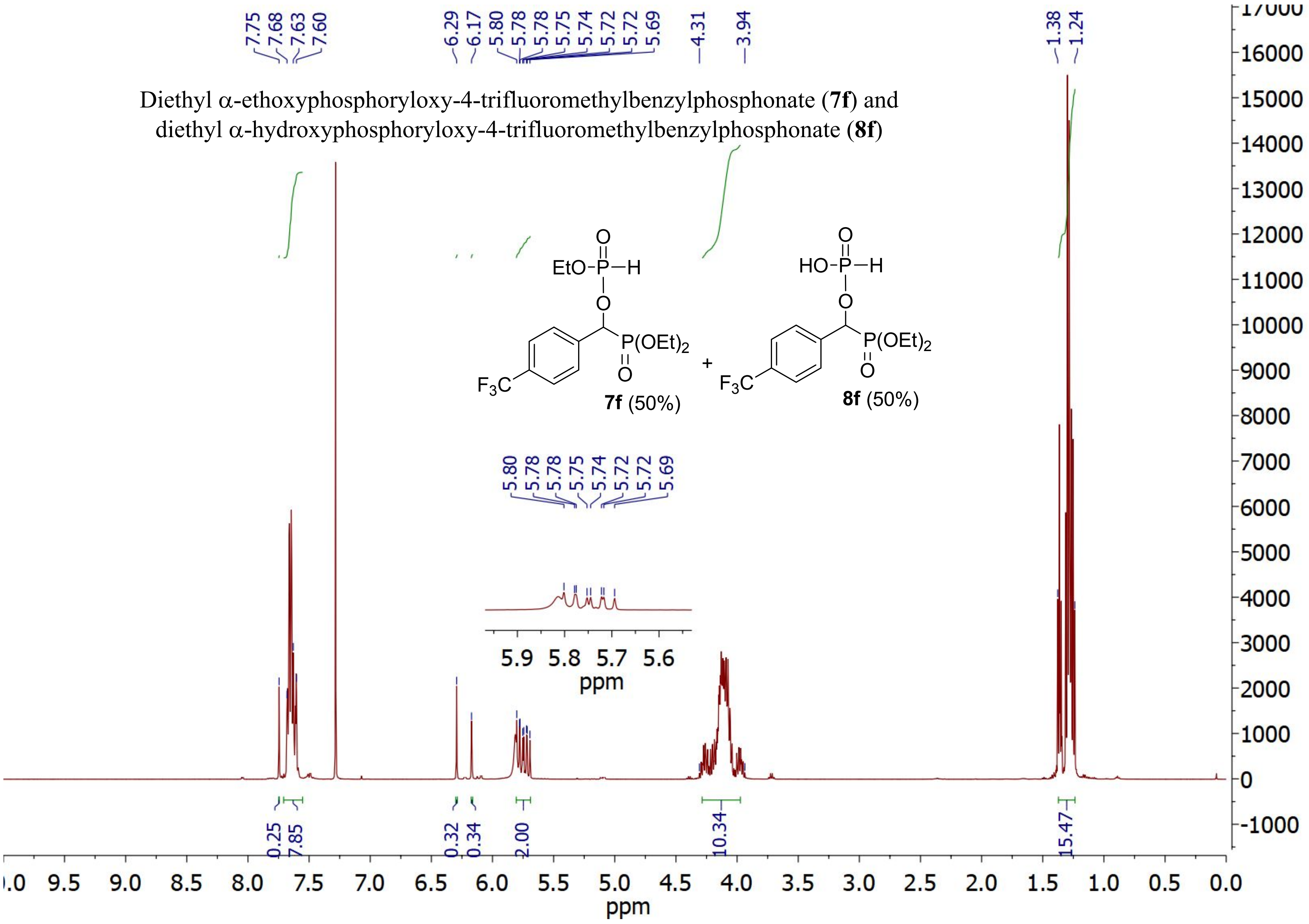

$^{31}P\ \{^{1}H\}$ NMR (202 MHz, $CDCl_3$)

Diethyl α-ethoxyphosphoryloxy-4-nitrobenzylphosphonate (**7g**) and diethyl α-hydroxyphosphoryloxy-4-nitrobenzylphosphonate (**8g**)

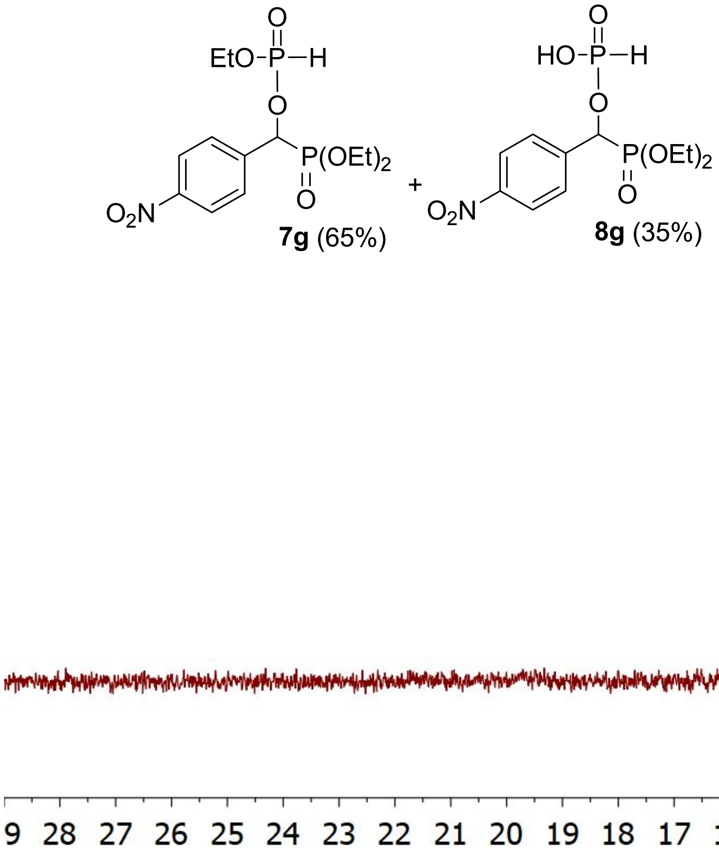

7g (65%) **8g** (35%)

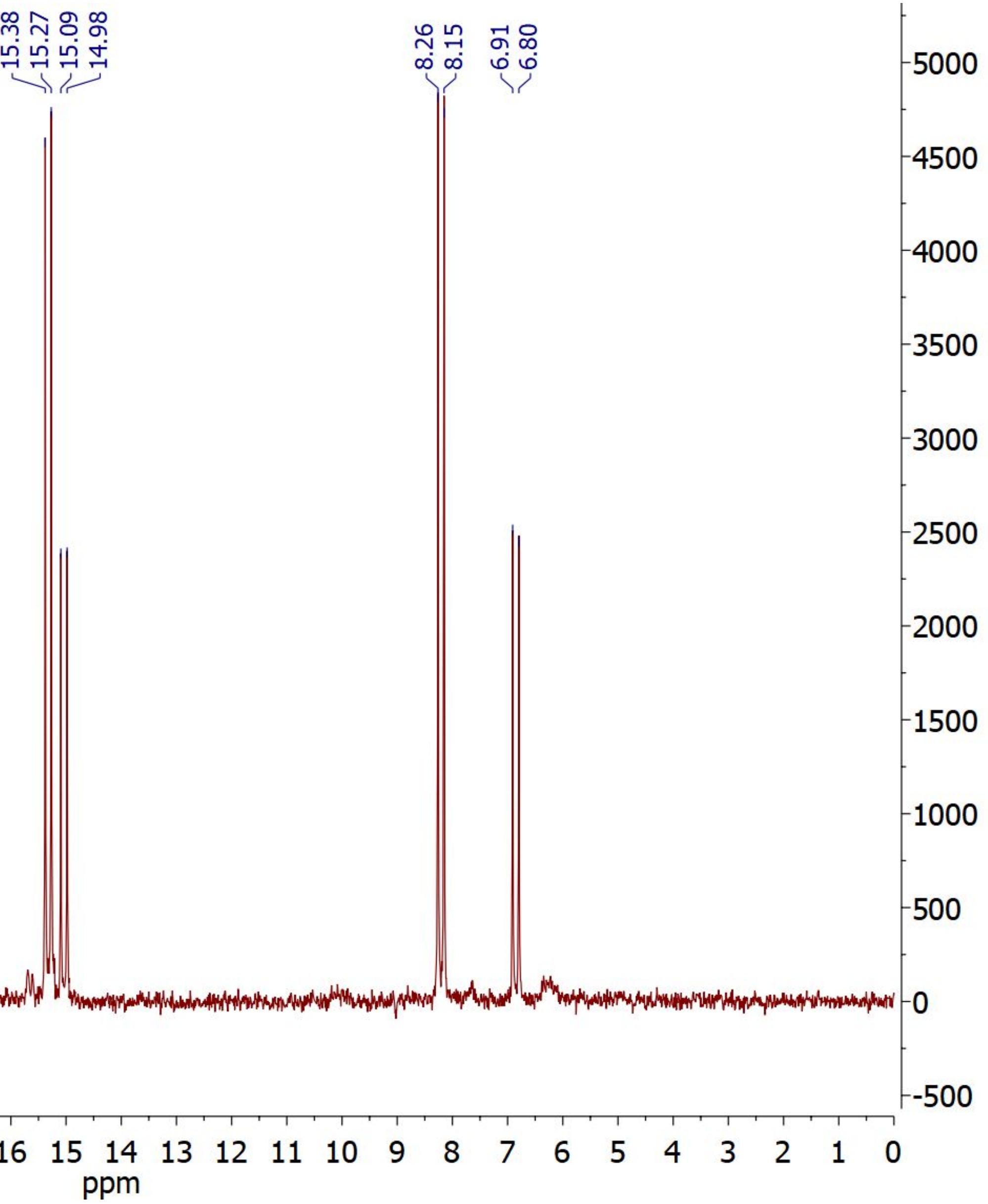

$^{13}C\ \{^1H\}$ NMR (126 MHz, $CDCl_3$)

Diethyl α-ethoxyphosphoryloxy-4-nitrobenzylphosphonate (**7g**) and diethyl α-hydroxyphosphoryloxy-4-nitrobenzylphosphonate (**8g**)

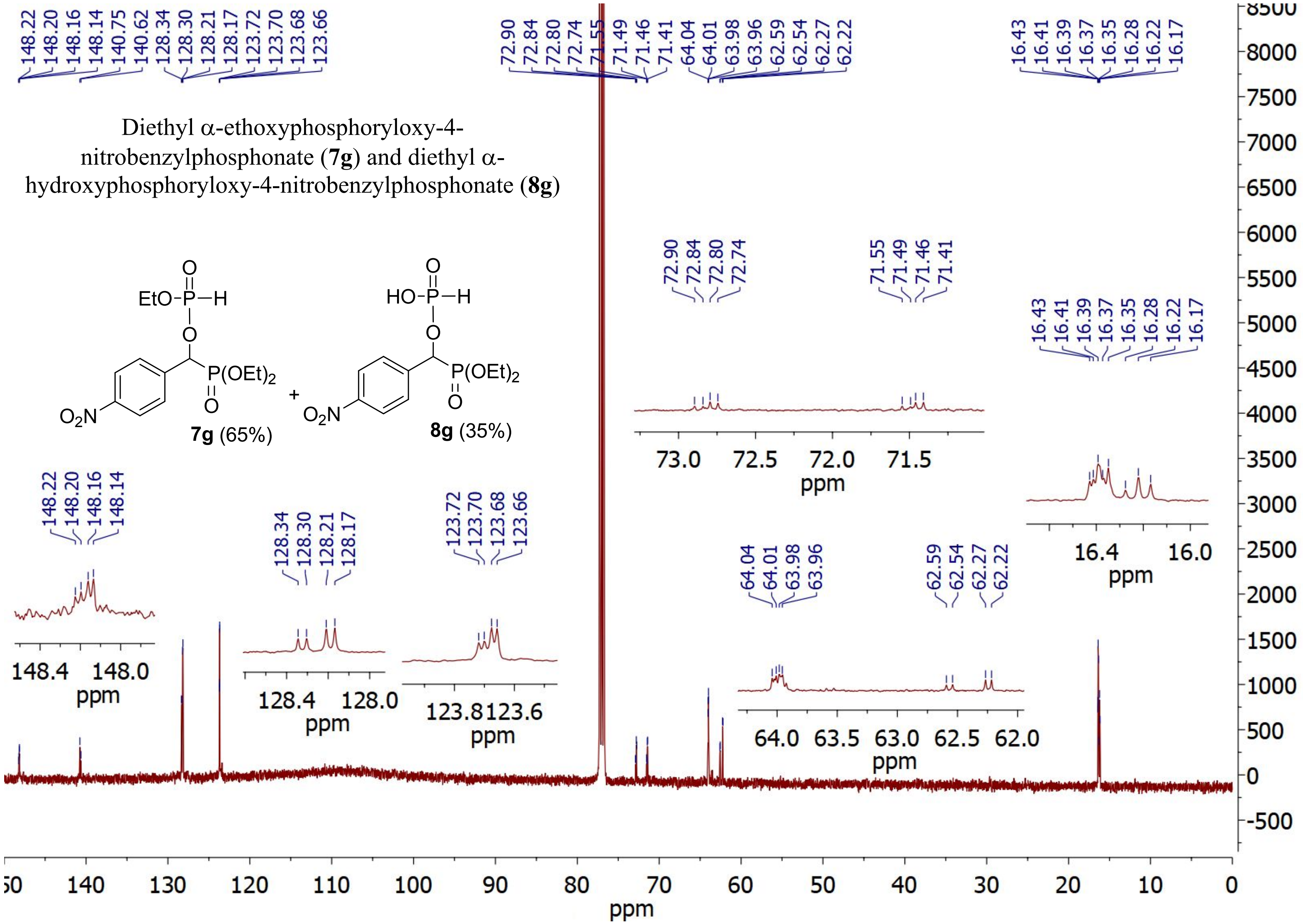

^{1}H NMR (500 MHz, $CDCl_3$)

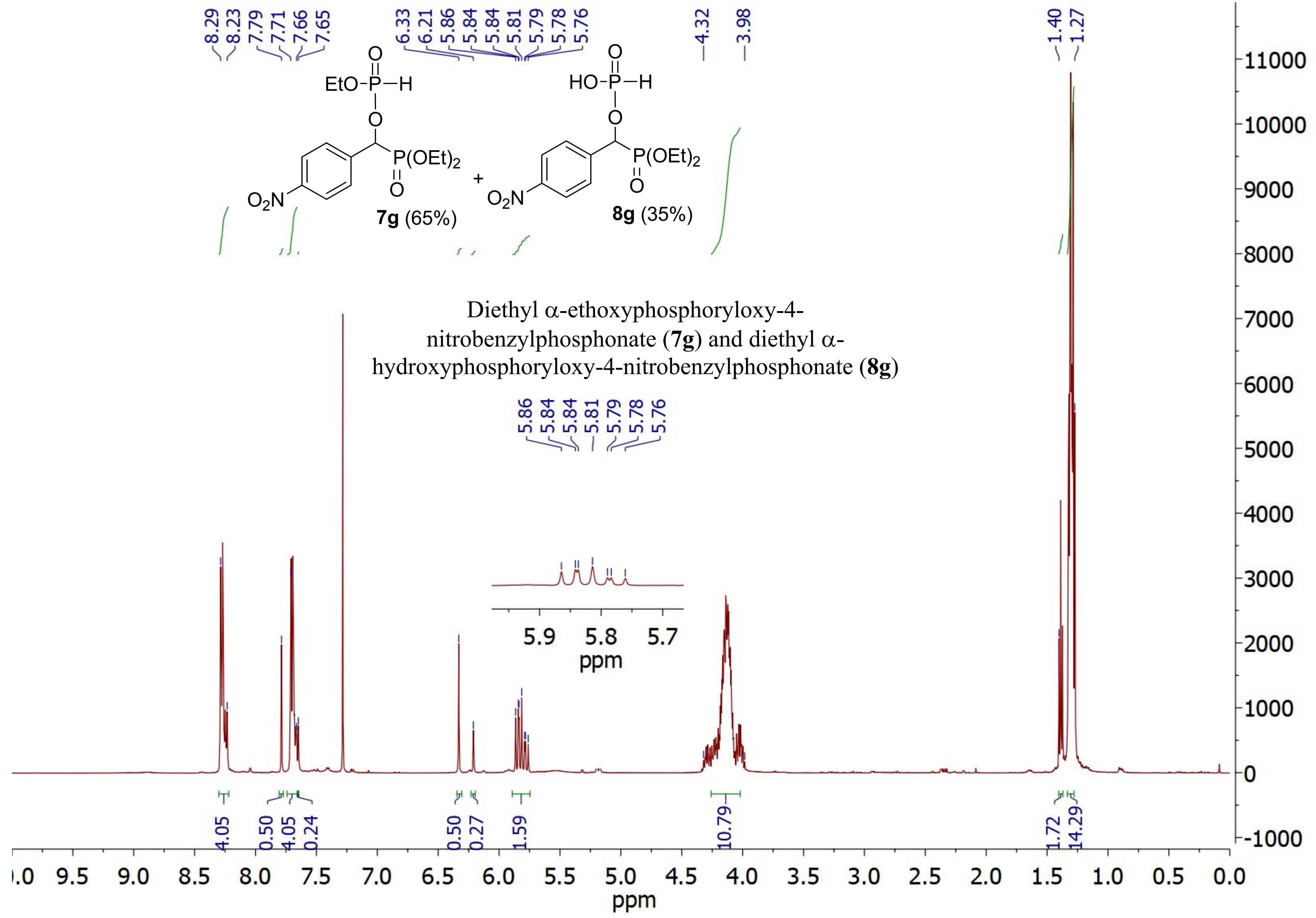

Diethyl α-ethoxyphosphoryloxy-4-nitrobenzylphosphonate (**7g**) and diethyl α-hydroxyphosphoryloxy-4-nitrobenzylphosphonate (**8g**)

$^{31}P\{^1H\}$ NMR (202 MHz, $CDCl_3$)

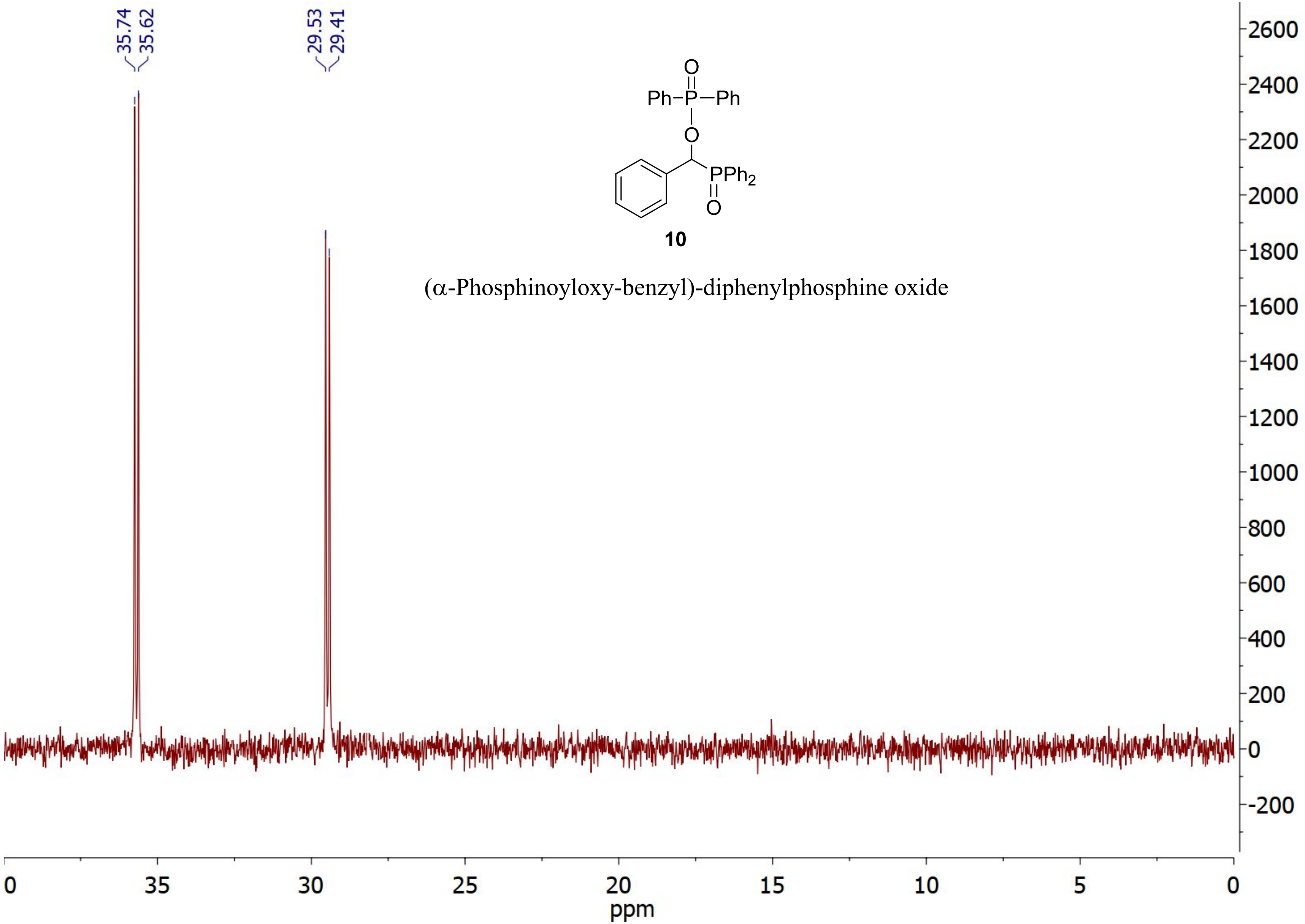

^{13}C {^{1}H} NMR (75 MHz, $CDCl_3$)

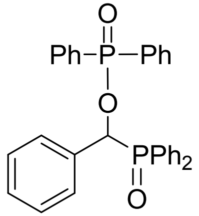

10

(α-Phosphinoyloxy-benzyl)-diphenylphosphine oxide

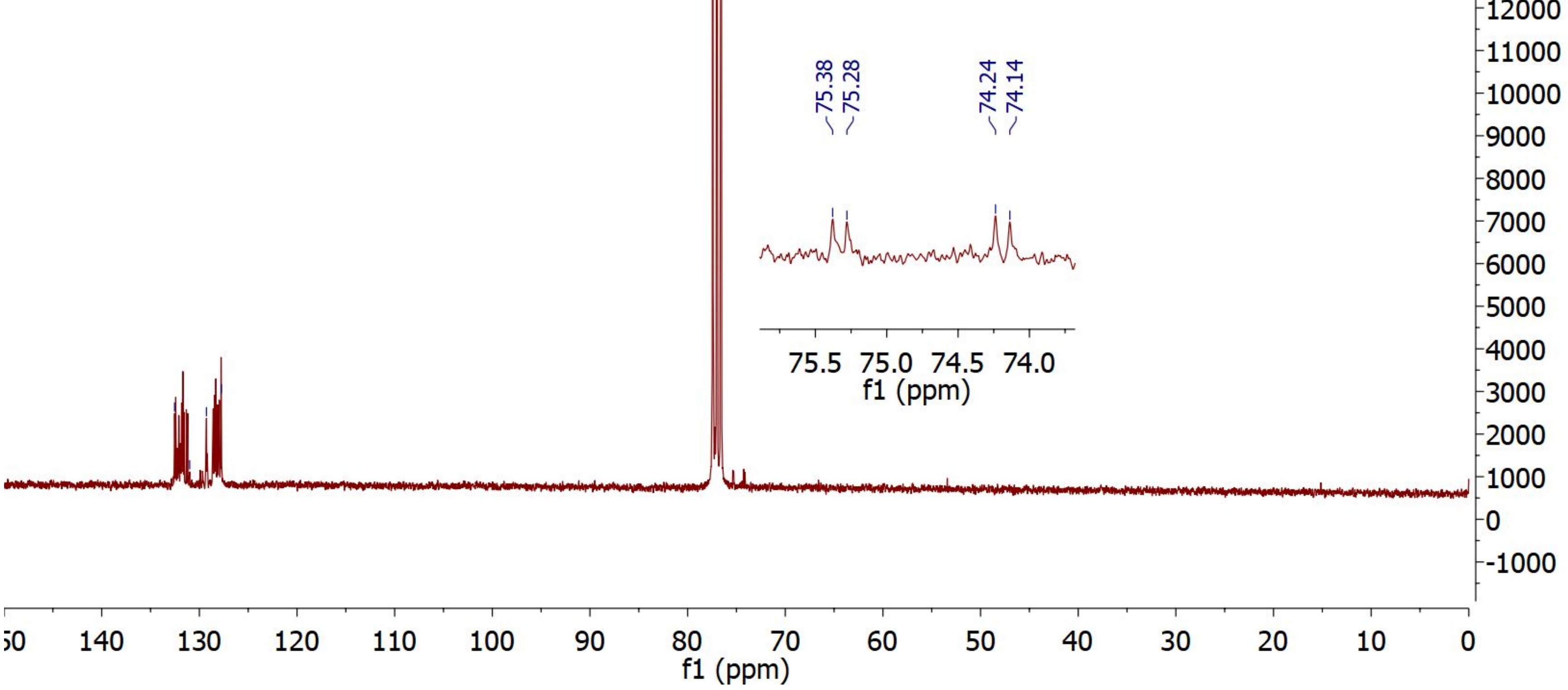

^{1}H NMR (500 MHz, $CDCl_3$)

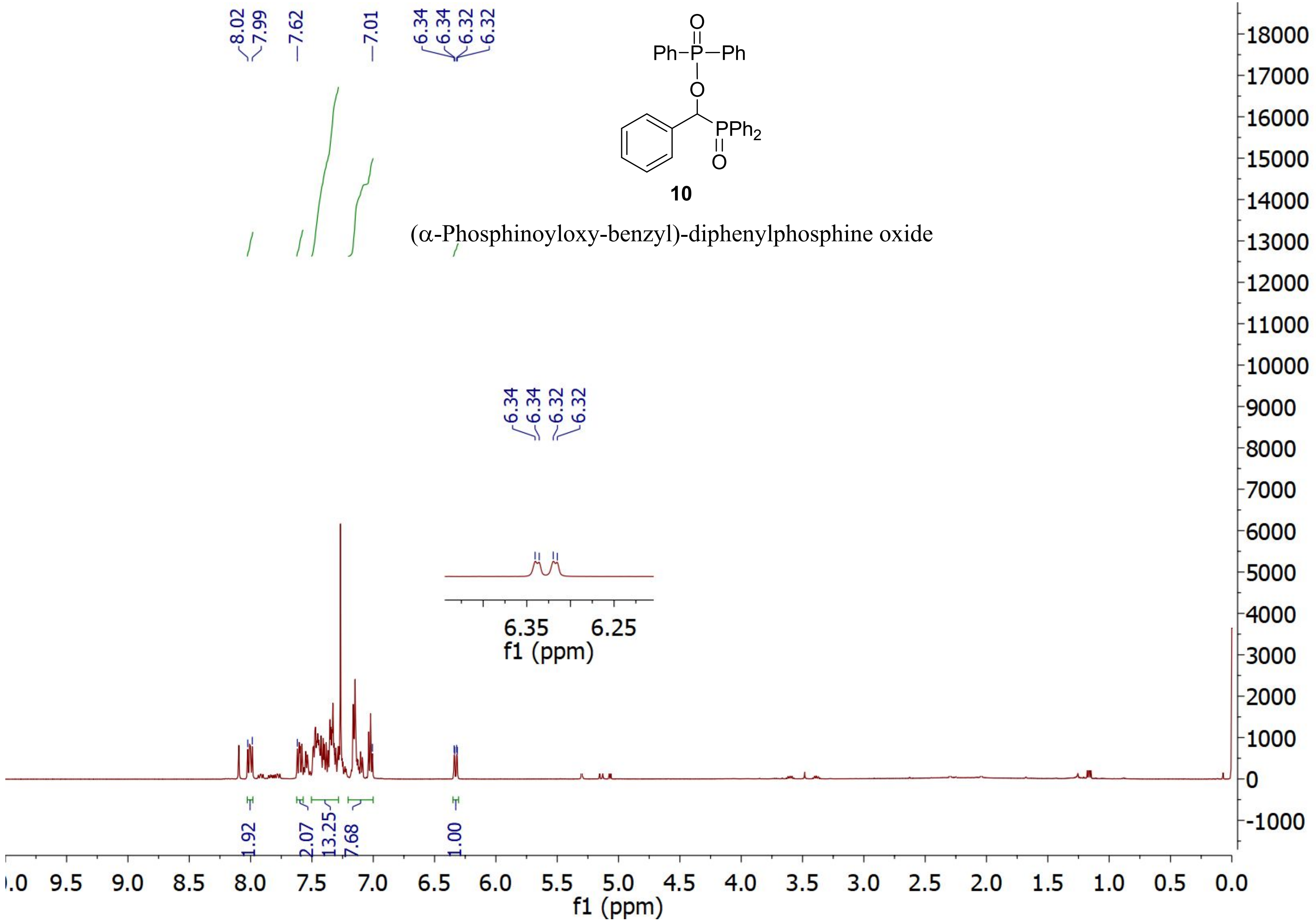

$^{31}P\{^1H\}$ NMR (202 MHz, $CDCl_3$)

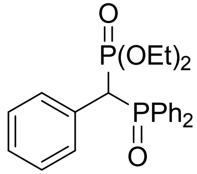

13

Diethyl α-(diphenylphosphinoyl)-
benzylphosphonate

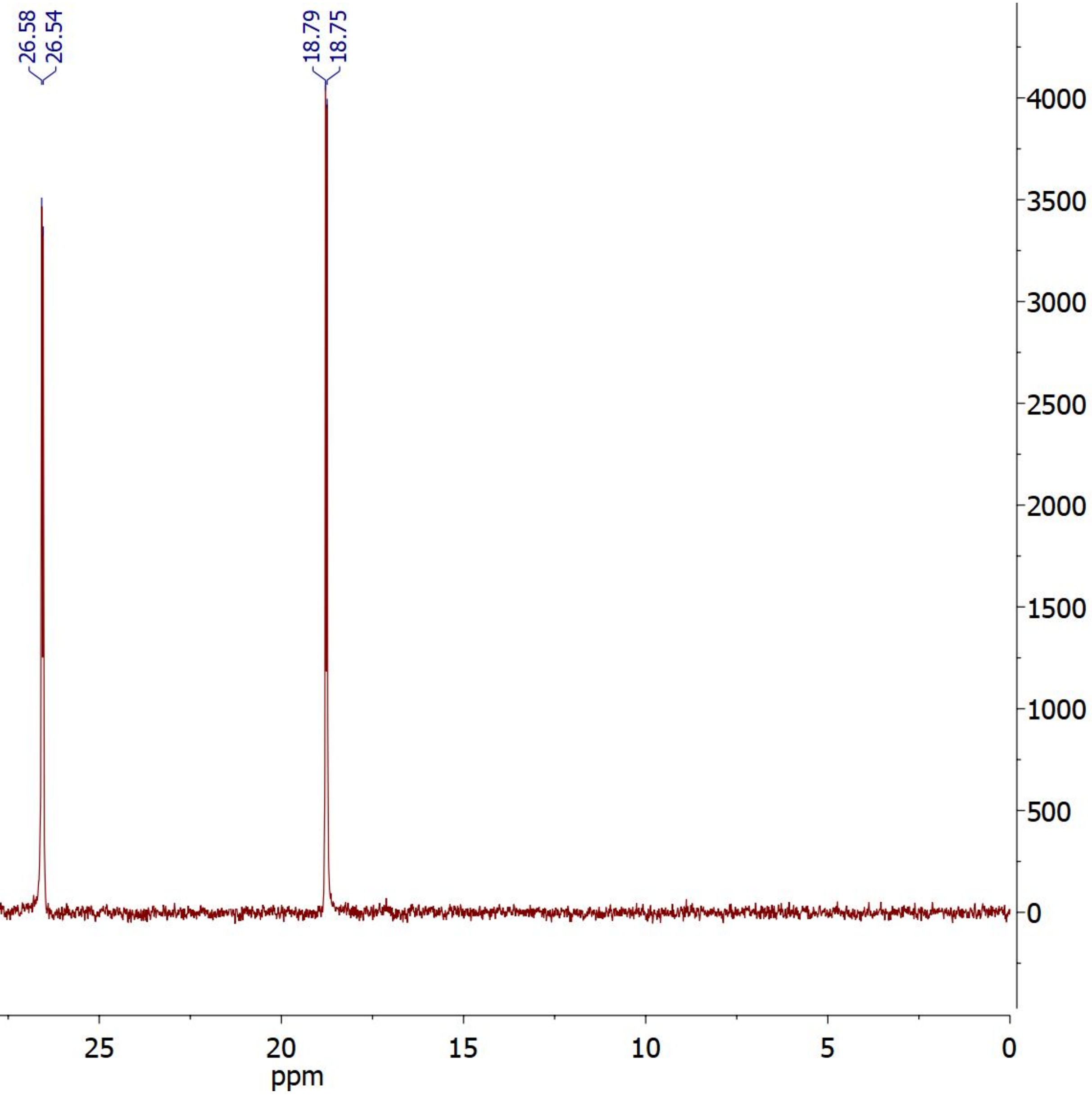

$^{13}C\ \{^{1}H\}$ NMR (126 MHz, $CDCl_3$)

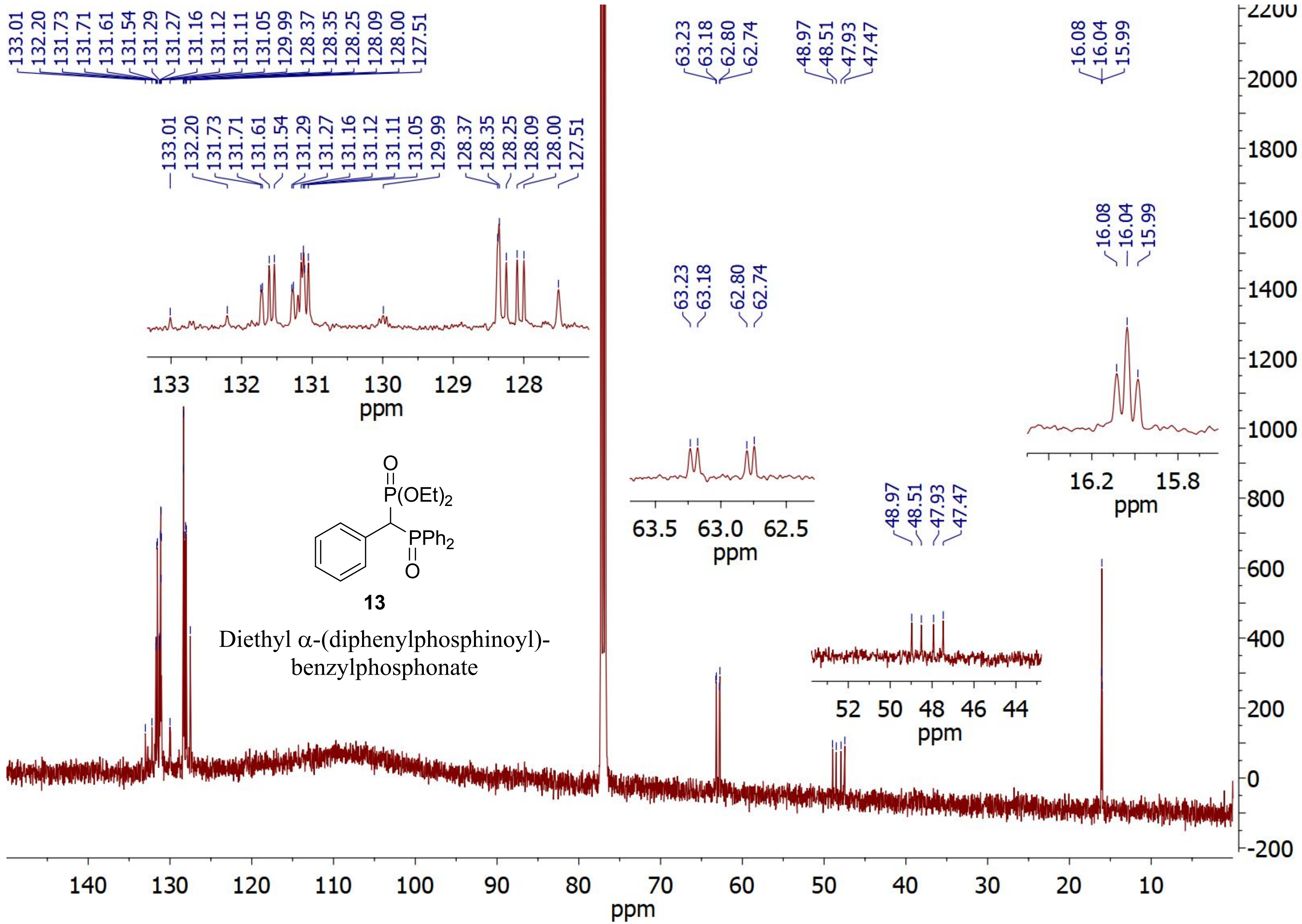

DEPT NMR (126 MHz, $CDCl_3$)

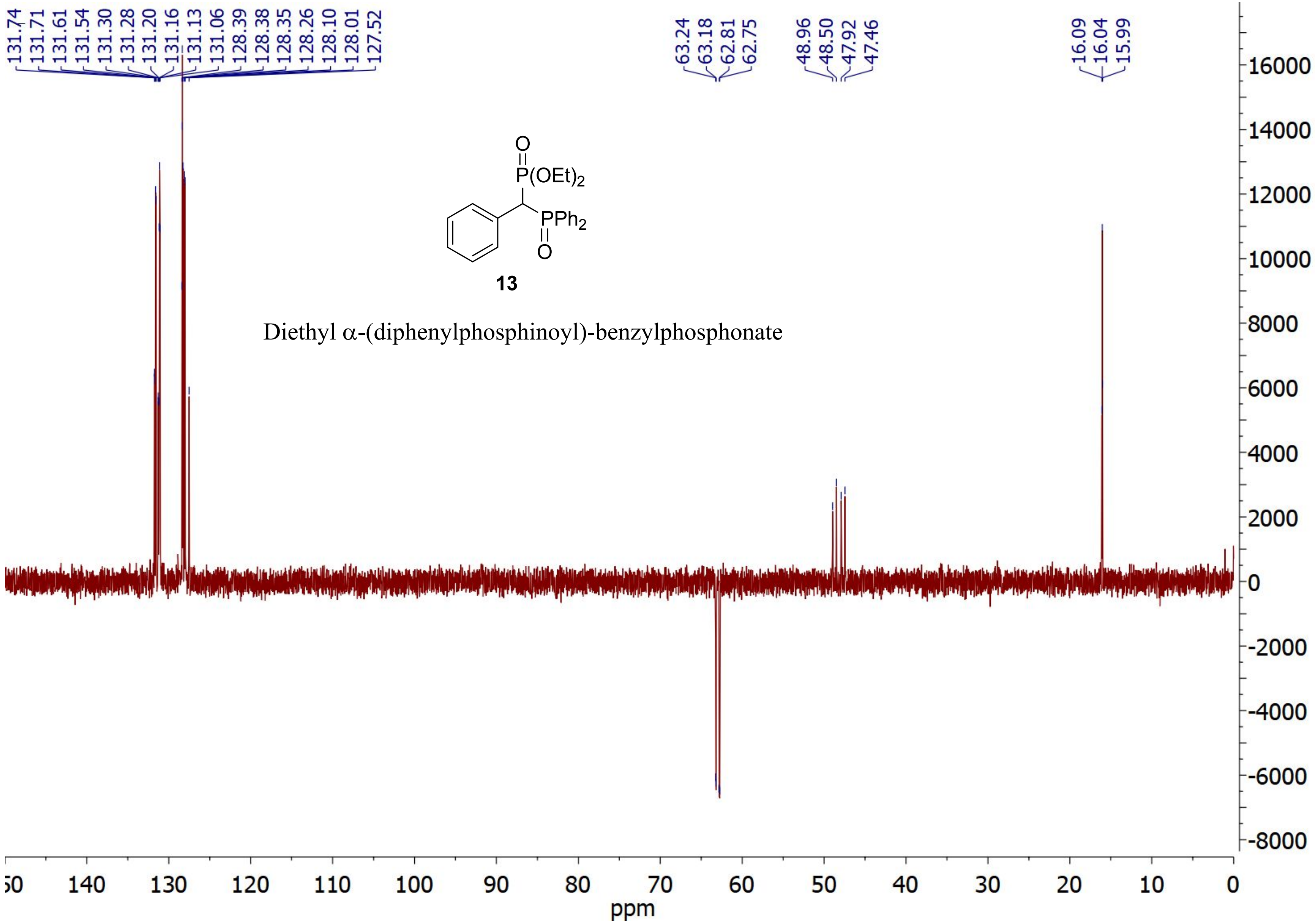

Diethyl α-(diphenylphosphinoyl)-benzylphosphonate

^{1}H NMR (500 MHz, $CDCl_3$)

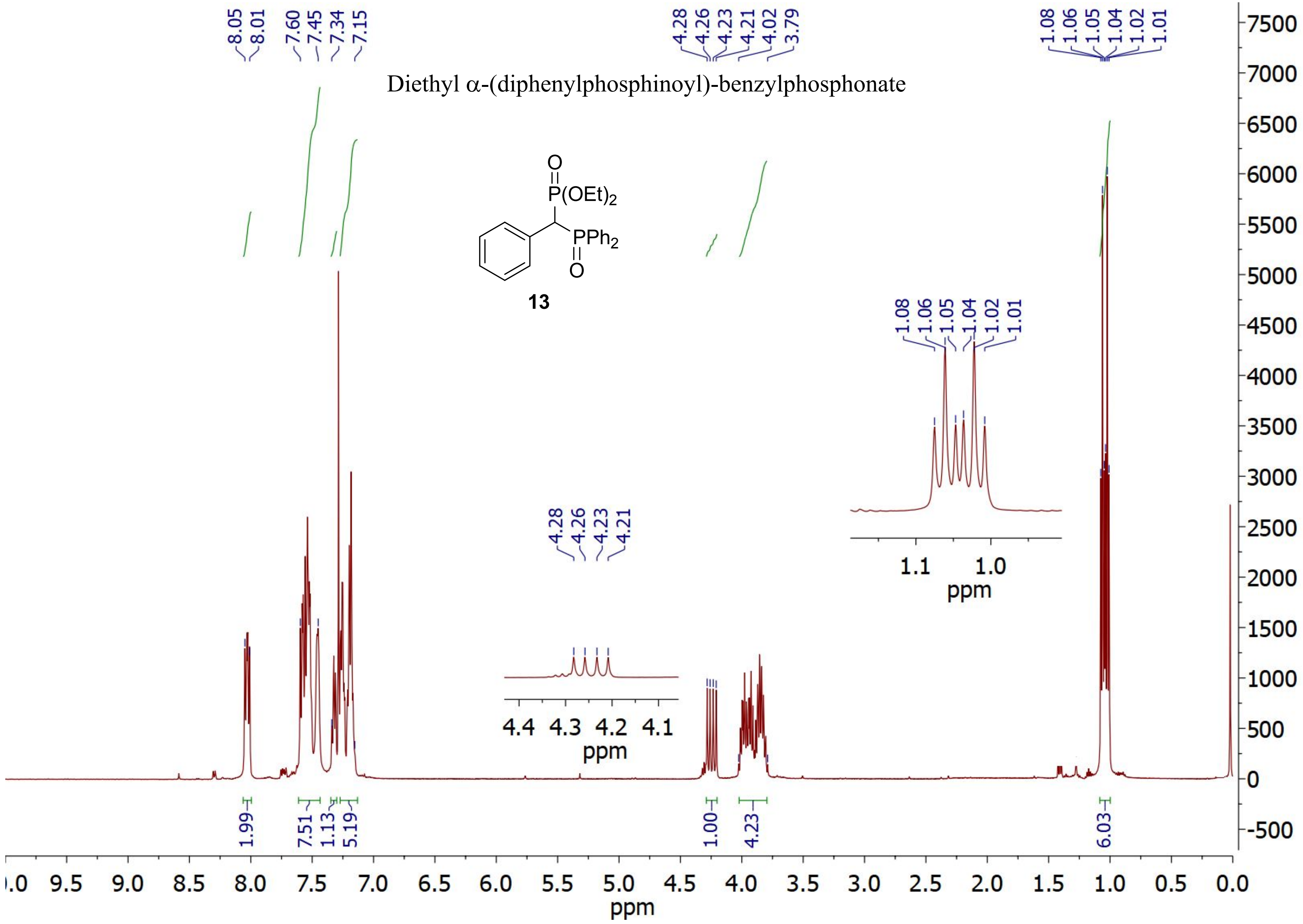

2. Theoretical calculations

Energy values obtained for the computation of the **1a** → **7a** and **9** → **11** transformations and negative frequencies of the computed transition states.

	E	ZPE	U	H	G	S	vib freq
MeOH	-115.6747439	-115.622784	-115.61825	-115.617037	-115.653317	59.44	
H_2O	-76.39960698	-76.37811	-76.374453	-76.37324	-76.401992	47.108	
dimethyl phosphite (oxo)	-647.3380095	-647.235924	-647.224573	-647.22336	-647.280174	93.084	
dimethyl phosphite (hydroxy)	-647.3357544	-647.234278	-647.221833	-647.22062	-647.280042	97.357	
diphenyl phosphine oxide (oxo)	-880.2648389	-880.066926	-880.048003	-880.046791	-880.123158	125.12	
diphenyl phosphine oxide (hydroxy)	-880.2656051	-880.067165	-880.04752	-880.046307	-880.124209	127.635	
**7a'**	-1524.458512	-1524.19183	-1524.160492	-1524.159279	-1524.266115	175.04	
**7a**	-1524.458899	-1524.191673	-1524.160796	-1524.159583	-1524.265301	173.21	
**11**	-2029.614905	-2029.127255	-2029.079577	-2029.078364	-2029.222512	236.173	
**1a**	-992.8224805	-992.605061	-992.581778	-992.580565	-992.667631	142.649	
**9**	-1225.744457	-1225.430613	-1225.400131	-1225.398918	-1225.501113	167.437	
**1a-DMP-MeOH**	-1755.870369	-1755.495679	-1755.451862	-1755.450649	-1755.585815	221.457	
**7a'-2MeOH**	-1755.880332	-1755.505129	-1755.462269	-1755.461057	-1755.593773	217.444	
**1a-7a' TS**	-1755.819607	-1755.447179	-1755.405354	-1755.404141	-1755.533827	212.479	-328.79
**9-DPPO-2H_2O**	-2258.858881	-2258.295043	-2258.234137	-2258.232924	-2258.407123	285.409	
**9-11 TS**	-2258.809556	-2258.249962	-2258.19421	-2258.192997	-2258.348621	254.975	-310.22
**11-3H_2O**	-2258.857648	-2258.293031	-2258.232439	-2258.231226	-2258.401131	278.374	

Coordinates of the computed structures

H_2O

Input orientation:

Center Number	Atomic Number	Atomic Type	Coordinates (Angstroms) X	Y	Z
1	8	0	-4.343542	1.595546	-1.396209
2	1	0	-3.504178	2.068264	-1.396209
3	1	0	-5.026319	2.275112	-1.396209

dimethyl phosphite (oxo)

Input orientation:

Center Number	Atomic Number	Atomic Type	Coordinates (Angstroms) X	Y	Z
1	15	0	-4.647792	0.386125	0.007613
2	8	0	-4.723338	0.999998	-1.471941
3	8	0	-3.603051	-0.642786	0.132829
4	8	0	-4.472781	1.644939	0.988291
5	6	0	-5.582973	2.116938	-1.716516
6	6	0	-5.567695	2.302702	1.632279
7	1	0	-5.766206	3.255330	1.135043
8	1	0	-6.473875	1.687230	1.619597
9	1	0	-6.586462	1.944258	-1.308296
10	1	0	-5.155154	3.027332	-1.287899
11	1	0	-5.966610	-0.022189	0.274513
12	1	0	-5.276005	2.483462	2.667516
13	1	0	-5.656201	2.224813	-2.798373

dimethyl phosphite (hydroxy)

Input orientation:

Center Number	Atomic Number	Atomic Type	Coordinates (Angstroms) X	Y	Z
1	15	0	-5.421113	0.610740	-0.025796
2	8	0	-4.699563	1.013440	-1.438984
3	8	0	-4.254133	-0.410165	0.521525
4	1	0	-4.573524	-1.319767	0.588416
5	8	0	-4.994889	1.964218	0.804099
6	6	0	-5.322855	2.042716	-2.206301
7	6	0	-5.484216	2.087056	2.137504
8	1	0	-6.567062	1.918505	2.183235
9	1	0	-4.977171	1.381547	2.804096

10	1	0	-6.405042	1.881448	-2.290258
11	1	0	-5.134827	3.022376	-1.756904
12	1	0	-5.268151	3.104939	2.464632
13	1	0	-4.880268	2.008849	-3.202691

MeOH

Input orientation:

Center Number	Atomic Number	Atomic Type	Coordinates (Angstroms) X	Y	Z
1	8	0	0.434921	1.619419	-2.116149
2	1	0	1.355671	1.407030	-2.303735
3	6	0	0.366447	2.933522	-1.589191
4	1	0	-0.687117	3.143326	-1.395538
5	1	0	0.918030	3.026058	-0.645556
6	1	0	0.744297	3.679860	-2.298683

----

diphenyl phosphine oxide (oxo)

Input orientation:

Center Number	Atomic Number	Atomic Type	Coordinates (Angstroms) X	Y	Z
1	15	0	-1.823138	-0.806882	-0.746688
2	8	0	-1.345929	-1.592871	-1.931200
3	6	0	-3.404948	0.055722	-0.939594
4	6	0	-4.531442	-0.659535	-1.362661
5	6	0	-3.506801	1.421907	-0.668552
6	6	0	-5.752408	-0.007757	-1.506096
7	1	0	-4.449737	-1.721649	-1.580869
8	6	0	-4.733232	2.072024	-0.809358
9	1	0	-2.630767	1.983621	-0.351764
10	6	0	-5.853327	1.356854	-1.227444
11	1	0	-6.625738	-0.561914	-1.835908
12	1	0	-4.810581	3.134250	-0.598925
13	1	0	-6.807760	1.862026	-1.340926
14	6	0	-1.992284	-1.827556	0.746214
15	6	0	-2.592526	-1.333997	1.909153
16	6	0	-1.481712	-3.126547	0.716063
17	6	0	-2.677756	-2.140805	3.040435
18	1	0	-3.002638	-0.326304	1.928885
19	6	0	-1.572906	-3.933356	1.850088
20	1	0	-1.024085	-3.495424	-0.197888
21	6	0	-2.167530	-3.439927	3.009840
22	1	0	-3.145816	-1.760441	3.943136

23	1	0	-1.179424	-4.945008	1.827008
24	1	0	-2.238558	-4.069245	3.892087
25	1	0	-0.979918	0.255183	-0.348772

diphenyl phosphine oxide (hydroxy)

Input orientation:

Center Number	Atomic Number	Atomic Type	Coordinates (Angstroms) X	Y	Z
1	15	0	-1.633315	-0.537683	-0.632823
2	8	0	-1.561976	-1.650596	-1.881927
3	6	0	-3.333083	0.133536	-0.843725
4	6	0	-4.432645	-0.696753	-1.098431
5	6	0	-3.521098	1.514705	-0.755708
6	6	0	-5.699978	-0.147788	-1.264748
7	1	0	-4.289912	-1.772884	-1.165722
8	6	0	-4.795719	2.065736	-0.909240
9	1	0	-2.668081	2.163275	-0.568151
10	6	0	-5.882446	1.234644	-1.166327
11	1	0	-6.549074	-0.794298	-1.466109
12	1	0	-4.934541	3.140229	-0.835770
13	1	0	-6.873455	1.660506	-1.292736
14	6	0	-1.948984	-1.734542	0.724466
15	6	0	-2.524122	-1.281416	1.918468
16	6	0	-1.549690	-3.069859	0.621278
17	6	0	-2.704520	-2.152368	2.989086
18	1	0	-2.845340	-0.245447	2.008004
19	6	0	-1.733897	-3.942119	1.695392
20	1	0	-1.108720	-3.426075	-0.304746
21	6	0	-2.309119	-3.486634	2.879349
22	1	0	-3.158618	-1.792082	3.907457
23	1	0	-1.427904	-4.980261	1.603776
24	1	0	-2.452171	-4.167043	3.713491
25	1	0	-0.941415	-1.358161	-2.561220

1a

Input orientation:

Center Number	Atomic Number	Atomic Type	Coordinates (Angstroms) X	Y	Z
1	8	0	-5.064305	1.443458	-1.457762
2	6	0	-5.778598	2.652528	-1.653004
3	1	0	-6.103848	2.988012	-0.662987
4	6	0	-4.983672	3.749422	-2.327608

5	6	0	-3.954152	3.447752	-3.220373
6	6	0	-5.298541	5.087451	-2.073386
7	6	0	-3.248454	4.471111	-3.851175
8	1	0	-3.688806	2.414469	-3.423435
9	6	0	-4.596150	6.109707	-2.705572
10	1	0	-6.099020	5.327550	-1.377763
11	6	0	-3.567769	5.803156	-3.597356
12	1	0	-2.447856	4.224471	-4.541784
13	1	0	-4.846256	7.145727	-2.497938
14	1	0	-3.015474	6.598793	-4.088008
15	15	0	-7.268608	2.175985	-2.610313
16	8	0	-8.241100	1.397064	-1.593027
17	8	0	-8.041660	3.553299	-2.811860
18	8	0	-6.914097	1.379920	-3.815041
19	6	0	-9.297837	3.552318	-3.512438
20	6	0	-8.148764	-0.032561	-1.479133
21	1	0	-9.173189	3.128732	-4.512048
22	1	0	-10.041866	2.984144	-2.948076
23	1	0	-7.239993	-0.310076	-0.938901
24	1	0	-8.154705	-0.494758	-2.468777
25	1	0	-4.972510	0.999005	-2.314636
26	1	0	-9.605556	4.594602	-3.588216
27	1	0	-9.024317	-0.351445	-0.913254

7a’

Input orientation:

Center Number	Atomic Number	Atomic Type	Coordinates (Angstroms) X	Y	Z
1	8	0	0.128351	-0.608138	0.713880
2	6	0	-0.647505	-0.081726	-0.358652
3	1	0	-0.156961	-0.280618	-1.321295
4	6	0	-2.041777	-0.656218	-0.374065
5	6	0	-2.602835	-1.198390	0.781481
6	6	0	-2.789649	-0.609854	-1.551510
7	6	0	-3.900237	-1.700969	0.752743
8	1	0	-2.013898	-1.231968	1.691458
9	6	0	-4.089300	-1.105702	-1.575013
10	1	0	-2.351220	-0.187529	-2.452399
11	6	0	-4.646375	-1.654379	-0.422462
12	1	0	-4.330086	-2.129762	1.652498
13	1	0	-4.663206	-1.070821	-2.495551
14	1	0	-5.657798	-2.047728	-0.441297
15	15	0	-0.631458	1.739611	-0.142663
16	8	0	-1.340376	2.351859	-1.446884
17	8	0	-1.710181	1.986891	0.996456

18	8	0	0.727355	2.300789	0.111934
19	6	0	-1.832040	3.313379	1.537409
20	6	0	-0.512615	2.653016	-2.580894
21	1	0	-0.865820	3.662197	1.908351
22	1	0	0.226724	3.412867	-2.321255
23	1	0	-1.179127	3.026500	-3.357557
24	15	0	1.700091	-1.059967	0.451169
25	8	0	2.242138	0.154913	-0.502080
26	8	0	1.396133	-2.139807	-0.755773
27	1	0	-2.544518	3.245724	2.358146
28	1	0	-2.212037	3.996650	0.773778
29	1	0	-0.000454	1.755588	-2.943131
30	1	0	1.956589	1.045846	-0.196034
31	6	0	2.538405	-2.663207	-1.425540
32	1	0	2.999209	-1.902473	-2.063073
33	1	0	3.286897	-3.033920	-0.713516
34	1	0	2.200219	-3.496846	-2.042610

7a

Input orientation:

Center Number	Atomic Number	Atomic Type	Coordinates (Angstroms) X	Y	Z
1	8	0	-0.051172	-1.291873	0.233032
2	6	0	-1.075707	-0.595885	-0.485694
3	1	0	-0.996936	-0.819278	-1.556150
4	6	0	-2.420875	-1.013906	0.046747
5	6	0	-2.621474	-1.127841	1.423992
6	6	0	-3.479595	-1.240643	-0.831363
7	6	0	-3.872783	-1.486179	1.913826
8	1	0	-1.794784	-0.937116	2.100846
9	6	0	-4.732956	-1.590820	-0.337353
10	1	0	-3.320824	-1.143430	-1.901876
11	6	0	-4.929560	-1.717448	1.035196
12	1	0	-4.024882	-1.579460	2.984308
13	1	0	-5.552907	-1.770782	-1.025154
14	1	0	-5.905285	-1.995775	1.420679
15	15	0	-0.816399	1.205941	-0.275894
16	8	0	0.555984	1.444934	-1.080174
17	8	0	-1.953741	1.735276	-1.273574
18	8	0	-0.820999	1.756363	1.095476
19	6	0	-2.052500	3.152382	-1.472051
20	6	0	1.593796	2.252908	-0.496553
21	1	0	-2.123794	3.670249	-0.512277
22	1	0	1.769284	1.966226	0.539076
23	1	0	1.317973	3.309056	-0.559538

24	15	0	1.467848	-1.455655	-0.281085
25	8	0	2.468864	-0.487522	0.205814
26	8	0	1.305245	-1.547528	-1.870578
27	1	0	-2.958874	3.326493	-2.050793
28	1	0	-1.185045	3.514654	-2.031910
29	1	0	2.492502	2.065345	-1.083042
30	6	0	2.085554	-0.706653	-2.736326
31	1	0	1.498173	0.171769	-3.007769
32	1	0	3.007668	-0.394282	-2.242082
33	1	0	2.318143	-1.296782	-3.623233
34	1	0	1.683826	-2.772118	0.116912

1a-DMP-MeOH

Input orientation:

Center Number	Atomic Number	Atomic Type	Coordinates (Angstroms) X	Y	Z
1	8	0	-0.836867	0.840092	2.249041
2	6	0	-0.914549	0.052345	1.084145
3	1	0	0.063768	-0.382543	0.825689
4	6	0	-1.920240	-1.062061	1.239493
5	6	0	-2.929406	-0.974707	2.199295
6	6	0	-1.860644	-2.175751	0.396507
7	6	0	-3.868242	-1.998478	2.317817
8	1	0	-2.968057	-0.107595	2.849923
9	6	0	-2.801892	-3.195433	0.515146
10	1	0	-1.074273	-2.241451	-0.351996
11	6	0	-3.808427	-3.108974	1.477168
12	1	0	-4.650115	-1.925985	3.067875
13	1	0	-2.746315	-4.059243	-0.140462
14	1	0	-4.541191	-3.904681	1.571552
15	15	0	-1.371319	1.153413	-0.318101
16	8	0	-0.260694	2.333164	-0.268125
17	8	0	-0.878131	0.279590	-1.568192
18	8	0	-2.760572	1.667305	-0.377334
19	6	0	-1.312545	0.660489	-2.882866
20	6	0	-0.620200	3.592821	0.324437
21	1	0	-2.391461	0.517536	-2.975579
22	1	0	-0.899421	3.462468	1.373087
23	1	0	-1.449591	4.044733	-0.223193
24	15	0	1.975095	-0.939047	-1.368022
25	8	0	2.030676	0.656965	-0.952095
26	8	0	1.413031	-0.923605	-2.906235
27	8	0	3.550785	-1.178175	-1.831440
28	6	0	2.345459	1.670349	-1.910162
29	6	0	4.544435	-1.107230	-0.810185

30	1	0	4.726146	-0.066716	-0.520655
31	1	0	4.250106	-1.686002	0.073701
32	1	0	3.276156	1.433030	-2.437051
33	1	0	1.524107	1.776572	-2.624177
34	1	0	-0.779363	0.011259	-3.575486
35	1	0	-1.062330	1.707018	-3.084220
36	1	0	0.265685	4.225357	0.255862
37	1	0	2.471561	2.605564	-1.361798
38	1	0	5.459959	-1.532413	-1.223254
39	8	0	1.714146	1.651764	1.687950
40	1	0	1.604410	1.559834	0.725446
41	6	0	2.618069	0.647913	2.125915
42	1	0	2.702925	0.735568	3.210999
43	1	0	2.253083	-0.360203	1.886241
44	1	0	3.610548	0.786841	1.683007
45	1	0	2.127665	-0.802560	-3.552803
46	1	0	0.042768	1.270397	2.238553

1a-7a' TS

Input orientation:

Center Number	Atomic Number	Atomic Type	Coordinates (Angstroms) X	Y	Z
1	8	0	-0.441473	-0.203174	0.050515
2	6	0	0.672378	0.077066	-0.727703
3	1	0	0.467894	-0.004864	-1.810715
4	6	0	1.325572	1.419130	-0.443084
5	6	0	0.836160	2.234282	0.577516
6	6	0	2.414426	1.858971	-1.204042
7	6	0	1.431004	3.468633	0.843310
8	1	0	-0.026717	1.900469	1.145595
9	6	0	3.009183	3.089215	-0.938778
10	1	0	2.798551	1.230039	-2.003324
11	6	0	2.520301	3.898220	0.089080
12	1	0	1.036329	4.098206	1.635671
13	1	0	3.853636	3.419936	-1.536250
14	1	0	2.982886	4.859021	0.293816
15	15	0	1.699858	-1.352656	-0.234124
16	8	0	1.117417	-2.624984	-1.028517
17	8	0	3.132779	-1.183476	-0.918388
18	8	0	1.702176	-1.542753	1.251049
19	6	0	4.158145	-2.145711	-0.623237
20	6	0	-0.093632	-3.251427	-0.571678
21	1	0	4.339566	-2.187442	0.453471
22	1	0	-0.948953	-2.604861	-0.784576
23	1	0	-0.030291	-3.459053	0.500834

24	15	0	-2.239679	0.595991	-0.490224
25	8	0	-2.360639	0.556347	1.247206
26	8	0	-2.670034	-0.940084	-0.938362
27	8	0	-3.890980	1.178361	-0.381953
28	6	0	-4.524483	1.361729	-1.628358
29	1	0	-5.361187	2.052948	-1.494586
30	1	0	-3.834999	1.790656	-2.371053
31	1	0	5.056085	-1.804514	-1.137697
32	1	0	3.869577	-3.131774	-0.996826
33	1	0	-0.175166	-4.190535	-1.120556
34	1	0	-4.908015	0.412049	-2.027070
35	8	0	-0.746402	-0.839205	2.444392
36	1	0	-1.500231	-0.202718	2.027705
37	6	0	0.012064	-0.241267	3.520733
38	1	0	0.746155	-0.976105	3.844604
39	1	0	0.511601	0.662793	3.168023
40	1	0	-0.688732	-0.015228	4.323546
41	1	0	-0.158149	-0.932326	1.628378
42	6	0	-3.628944	-1.698027	-0.217052
43	1	0	-3.207712	-2.060326	0.729230
44	1	0	-4.531605	-1.112628	-0.010670
45	1	0	-3.890985	-2.557080	-0.838999
46	1	0	-3.268178	0.793975	1.495821

7a'-2MeOH

Input orientation:

Center Number	Atomic Number	Atomic Type	Coordinates (Angstroms) X	Y	Z
1	8	0	-0.452309	0.163500	-0.512957
2	6	0	0.930501	0.024454	-0.810324
3	1	0	1.100789	-0.091108	-1.891014
4	6	0	1.771727	1.159753	-0.273934
5	6	0	3.073202	1.343625	-0.751173
6	6	0	1.264309	2.019361	0.700791
7	6	0	3.862031	2.375746	-0.251577
8	1	0	3.467149	0.675841	-1.512991
9	6	0	2.055987	3.055643	1.196052
10	1	0	0.247274	1.882445	1.052801
11	6	0	3.354741	3.234256	0.725025
12	1	0	4.870568	2.514162	-0.628919
13	1	0	1.653794	3.724860	1.950533
14	1	0	3.968702	4.042106	1.111244
15	15	0	1.302690	-1.593282	-0.039475
16	8	0	0.553486	-2.684873	-0.939833
17	8	0	2.829563	-1.816107	-0.421238

18	8	0	0.965433	-1.684975	1.410853
19	6	0	3.520736	-2.964299	0.106182
20	6	0	-0.794905	-3.089421	-0.634970
21	1	0	3.492269	-2.952093	1.197908
22	1	0	-1.497950	-2.347531	-1.017905
23	1	0	-0.921276	-3.223850	0.442224
24	1	0	-0.664717	-1.345124	1.955909
25	15	0	-1.419156	1.002295	-1.596521
26	8	0	-3.389777	-1.112713	0.326241
27	8	0	-2.558670	-0.095693	-1.880030
28	1	0	-2.912886	-0.528341	-1.028458
29	8	0	-2.112664	1.905200	-0.387879
30	6	0	-4.401476	-0.290053	0.894540
31	6	0	-3.166415	2.776899	-0.792928
32	1	0	-2.838743	3.450854	-1.593828
33	1	0	-4.039541	2.209857	-1.135188
34	1	0	-5.255698	-0.291828	0.213087
35	1	0	-4.724568	-0.689590	1.861771
36	1	0	4.549862	-2.884294	-0.241088
37	1	0	3.069886	-3.883075	-0.277349
38	1	0	-0.948357	-4.041670	-1.142515
39	1	0	-4.048584	0.740496	1.025699
40	1	0	-3.442426	3.371975	0.079306
41	8	0	-1.568246	-1.103554	2.250251
42	1	0	-2.651662	-1.189791	0.987980
43	6	0	-1.544044	0.262726	2.652412
44	1	0	-0.617618	0.491728	3.189806
45	1	0	-1.639111	0.928673	1.785717
46	1	0	-2.386258	0.427250	3.330553

9

Input orientation:

Center Number	Atomic Number	Atomic Type	Coordinates (Angstroms) X	Y	Z
1	8	0	0.854259	0.517348	-1.917764
2	6	0	0.872516	-0.283824	-0.751025
3	1	0	-0.141118	-0.621941	-0.486159
4	6	0	1.759887	-1.486381	-0.962988
5	6	0	2.520792	-1.624301	-2.125465
6	6	0	1.836705	-2.467970	0.032243
7	6	0	3.349050	-2.734304	-2.291393
8	1	0	2.454373	-0.868536	-2.900606
9	6	0	2.667362	-3.571980	-0.136970
10	1	0	1.254271	-2.355677	0.942851
11	6	0	3.426398	-3.709290	-1.299741

12	1	0	3.934220	-2.834850	-3.200715
13	1	0	2.721723	-4.326683	0.641844
14	1	0	4.072765	-4.571965	-1.430598
15	15	0	1.419189	0.703699	0.737961
16	8	0	1.126005	-0.004661	2.030838
17	6	0	3.173958	1.093549	0.503750
18	6	0	3.733255	1.382096	-0.747432
19	6	0	3.982399	1.071399	1.643785
20	6	0	5.093959	1.662773	-0.847664
21	1	0	3.111513	1.372079	-1.638734
22	6	0	5.342122	1.357440	1.537255
23	1	0	3.541391	0.819314	2.604342
24	6	0	5.896479	1.654823	0.293428
25	1	0	5.528768	1.880431	-1.818430
26	1	0	5.969167	1.340128	2.423353
27	1	0	6.957405	1.871806	0.211292
28	6	0	0.470744	2.252431	0.577968
29	6	0	0.833405	3.299753	-0.277585
30	6	0	-0.696444	2.354622	1.343455
31	6	0	0.026672	4.432009	-0.373441
32	1	0	1.748809	3.243074	-0.860650
33	6	0	-1.499125	3.489797	1.246197
34	1	0	-0.956945	1.548277	2.024050
35	6	0	-1.139517	4.526551	0.386288
36	1	0	0.314241	5.243581	-1.034953
37	1	0	-2.400374	3.565979	1.846724
38	1	0	-1.762925	5.412636	0.312987
39	1	0	0.142376	1.166848	-1.843451

9-DPPO-2H_2O

Center Number	Atomic Number	Atomic Type	Coordinates (Angstroms) X	Y	Z
1	6	0	0.733574	0.666826	0.095131
2	6	0	1.169249	2.112795	0.004932
3	6	0	1.954384	2.539345	-1.071257
4	6	0	0.824568	3.026040	1.005286
5	6	0	2.390934	3.860316	-1.147653
6	1	0	2.227137	1.831521	-1.851774
7	6	0	1.256695	4.348569	0.924392
8	1	0	0.216167	2.707676	1.847700
9	6	0	2.041634	4.768649	-0.148896
10	1	0	2.998736	4.179681	-1.988880
11	1	0	0.981427	5.050895	1.705246
12	1	0	2.377314	5.799510	-0.208166
13	15	0	2.000098	-0.338673	1.021119

14	8	0	2.279485	0.166628	2.418724
15	15	0	-2.952579	-1.121053	-0.073539
16	8	0	-4.288154	-2.045645	-0.469258
17	6	0	-2.212885	-0.889224	-1.745435
18	6	0	-2.157741	-1.995834	-2.604807
19	6	0	-1.609591	0.309761	-2.146694
20	6	0	-1.511294	-1.907715	-3.837279
21	1	0	-2.626808	-2.930974	-2.307460
22	6	0	-0.961796	0.396590	-3.378999
23	1	0	-1.640603	1.175155	-1.489448
24	6	0	-0.906760	-0.711776	-4.224711
25	1	0	-1.480224	-2.772472	-4.493587
26	1	0	-0.501941	1.334820	-3.676799
27	1	0	-0.401778	-0.641756	-5.183439
28	6	0	-3.689067	0.538022	0.222202
29	6	0	-3.487409	1.134011	1.470272
30	6	0	-4.477349	1.197398	-0.729290
31	6	0	-4.058220	2.372051	1.764971
32	1	0	-2.870657	0.633029	2.213845
33	6	0	-5.058519	2.427936	-0.433831
34	1	0	-4.625118	0.760178	-1.715693
35	6	0	-4.846423	3.016954	0.814187
36	1	0	-3.884667	2.829692	2.734079
37	1	0	-5.667377	2.932552	-1.178019
38	1	0	-5.293182	3.980210	1.041493
39	6	0	3.481961	-0.289987	-0.020632
40	6	0	3.553697	-0.965770	-1.243550
41	6	0	4.540595	0.520515	0.397395
42	6	0	4.682862	-0.825520	-2.046774
43	1	0	2.736970	-1.606486	-1.568710
44	6	0	5.669111	0.656920	-0.409338
45	1	0	4.468287	1.039194	1.349354
46	6	0	5.738399	-0.012450	-1.630397
47	1	0	4.741557	-1.353169	-2.993688
48	1	0	6.491775	1.286633	-0.085196
49	1	0	6.618081	0.094402	-2.257937
50	6	0	1.337287	-2.025488	1.010959
51	6	0	1.664326	-2.855223	2.087796
52	6	0	0.498999	-2.497990	-0.005465
53	6	0	1.171143	-4.156876	2.138202
54	1	0	2.288623	-2.469966	2.889325
55	6	0	0.002042	-3.798137	0.053085
56	1	0	0.209638	-1.856151	-0.834634
57	6	0	0.340450	-4.627789	1.122312
58	1	0	1.423489	-4.797619	2.977243
59	1	0	-0.657872	-4.156826	-0.731533
60	1	0	-0.052391	-5.639053	1.167826
61	8	0	-0.538558	0.474072	0.662843

62	1	0	-0.521385	0.699245	1.624750
63	1	0	0.699597	0.249960	-0.919971
64	8	0	-0.274302	0.744201	3.350253
65	1	0	0.693660	0.606304	3.313644
66	1	0	-0.656606	-0.139452	3.502773
67	1	0	-4.850375	-1.658019	-1.157217
68	8	0	-1.412855	-1.767328	2.934086
69	1	0	-1.461892	-1.722170	1.963281
70	1	0	-2.266445	-2.123573	3.208016

9-11 TS

Center Number	Atomic Number	Atomic Type	Coordinates (Angstroms) X	Y	Z
1	6	0	-0.239357	-0.690389	-0.130137
2	6	0	-0.998182	-1.937973	-0.530899
3	6	0	-1.270936	-2.207711	-1.872522
4	6	0	-1.446172	-2.830253	0.448944
5	6	0	-2.007355	-3.337333	-2.233973
6	1	0	-0.892641	-1.540432	-2.644314
7	6	0	-2.179888	-3.957000	0.091738
8	1	0	-1.211848	-2.631284	1.491184
9	6	0	-2.469825	-4.209690	-1.251460
10	1	0	-2.208741	-3.538953	-3.281894
11	1	0	-2.528717	-4.639661	0.860800
12	1	0	-3.043446	-5.088823	-1.528937
13	15	0	-1.419567	0.659671	0.525629
14	8	0	-0.942697	1.286341	1.837227
15	15	0	2.401879	-0.224075	1.151009
16	8	0	4.053004	0.714582	1.728537
17	6	0	2.112520	1.208426	-0.007008
18	6	0	1.743093	2.445347	0.539653
19	6	0	2.261222	1.117437	-1.397713
20	6	0	1.522628	3.554155	-0.276076
21	1	0	1.632778	2.551679	1.615485
22	6	0	2.032931	2.224615	-2.217242
23	1	0	2.561460	0.175616	-1.850237
24	6	0	1.661357	3.446206	-1.658977
25	1	0	1.234086	4.501139	0.171122
26	1	0	2.148972	2.129386	-3.293373
27	1	0	1.480767	4.307316	-2.295816
28	6	0	3.264448	-1.414248	0.014632
29	6	0	2.583345	-2.533557	-0.483220
30	6	0	4.615434	-1.258375	-0.325973
31	6	0	3.223965	-3.451965	-1.315768
32	1	0	1.545558	-2.692149	-0.212086
33	6	0	5.255311	-2.175261	-1.158649

34	1	0	5.176677	-0.420377	0.071388
35	6	0	4.561608	-3.275114	-1.662141
36	1	0	2.670709	-4.309325	-1.688773
37	1	0	6.302013	-2.029282	-1.410238
38	1	0	5.061134	-3.989785	-2.309560
39	6	0	-3.061934	-0.066016	0.794632
40	6	0	-3.889862	-0.437360	-0.270355
41	6	0	-3.459150	-0.317308	2.109950
42	6	0	-5.102288	-1.071116	-0.017706
43	1	0	-3.589408	-0.241492	-1.296784
44	6	0	-4.676734	-0.950390	2.358949
45	1	0	-2.814360	-0.015658	2.930433
46	6	0	-5.493978	-1.331668	1.296960
47	1	0	-5.740344	-1.363886	-0.845632
48	1	0	-4.984249	-1.144679	3.381785
49	1	0	-6.439824	-1.828320	1.491181
50	6	0	-1.612314	1.950546	-0.731847
51	6	0	-1.863621	3.253407	-0.287342
52	6	0	-1.542619	1.682961	-2.101630
53	6	0	-2.045647	4.279119	-1.210352
54	1	0	-1.897057	3.456782	0.779387
55	6	0	-1.726996	2.713022	-3.023367
56	1	0	-1.339741	0.677096	-2.459407
57	6	0	-1.978159	4.009266	-2.577971
58	1	0	-2.235386	5.290074	-0.862978
59	1	0	-1.666410	2.502003	-4.086502
60	1	0	-2.117590	4.811282	-3.296621
61	8	0	0.667656	-0.924410	0.888867
62	1	0	0.922247	-0.314607	3.151108
63	1	0	0.223937	-0.257245	-1.028732
64	8	0	0.637560	0.532957	3.547545
65	1	0	-0.040026	0.897882	2.801207
66	1	0	1.596783	1.093576	3.615728
67	1	0	4.404664	1.385778	1.125927
68	8	0	2.799099	1.669302	3.620821
69	1	0	3.436539	1.285908	2.827406
70	1	0	3.262731	1.625639	4.465431

11-3H_2O

Center Number	Atomic Number	Atomic Type	Coordinates (Angstroms) X	Y	Z
1	6	0	-0.453525	-0.373609	-0.176366
2	6	0	-1.764707	-1.082519	-0.418772
3	6	0	-2.638761	-1.324988	0.641592
4	6	0	-2.099050	-1.520962	-1.704509
5	6	0	-3.847964	-1.983354	0.421830

6	1	0	-2.378531	-0.986688	1.642228
7	6	0	-3.306798	-2.177255	-1.923794
8	1	0	-1.412846	-1.337293	-2.526362
9	6	0	-4.184294	-2.407856	-0.862265
10	1	0	-4.527294	-2.157526	1.250538
11	1	0	-3.563827	-2.510934	-2.924659
12	1	0	-5.127283	-2.917226	-1.036813
13	15	0	0.951034	-1.552694	-0.473652
14	8	0	1.004951	-2.049506	-1.898588
15	15	0	-0.647524	2.326713	-0.746955
16	8	0	2.633356	2.305883	-1.616739
17	6	0	0.037199	2.593503	0.945313
18	6	0	1.190734	3.386382	1.007623
19	6	0	-0.466081	2.042270	2.135993
20	6	0	1.838800	3.609296	2.223647
21	1	0	1.590305	3.818112	0.092912
22	6	0	0.184875	2.256061	3.348546
23	1	0	-1.379574	1.452900	2.120928
24	6	0	1.341729	3.038427	3.392666
25	1	0	2.732950	4.224859	2.252564
26	1	0	-0.214173	1.821298	4.260286
27	1	0	1.845447	3.208157	4.339858
28	6	0	-2.461111	2.213356	-0.427918
29	6	0	-3.238990	1.559418	-1.395078
30	6	0	-3.113602	2.891723	0.608306
31	6	0	-4.625102	1.519967	-1.289152
32	1	0	-2.752609	1.065233	-2.231761
33	6	0	-4.506245	2.867932	0.705848
34	1	0	-2.542691	3.447664	1.346217
35	6	0	-5.263493	2.169981	-0.231296
36	1	0	-5.206211	0.986065	-2.035213
37	1	0	-4.995789	3.395415	1.519177
38	1	0	-6.345772	2.143468	-0.147968
39	6	0	0.641112	-2.895991	0.702399
40	6	0	0.760951	-2.727936	2.086079
41	6	0	0.197908	-4.113885	0.179466
42	6	0	0.435975	-3.776943	2.942399
43	1	0	1.114349	-1.787612	2.501373
44	6	0	-0.124570	-5.161258	1.040733
45	1	0	0.109557	-4.228857	-0.897178
46	6	0	-0.007986	-4.992163	2.419418
47	1	0	0.533007	-3.648002	4.015873
48	1	0	-0.467897	-6.107459	0.634511
49	1	0	-0.259855	-5.808927	3.089001
50	6	0	2.475273	-0.688275	-0.007688
51	6	0	3.574405	-0.867436	-0.854642
52	6	0	2.591907	0.132292	1.122609
53	6	0	4.788018	-0.245709	-0.566239

54	1	0	3.467501	-1.475600	-1.748158
55	6	0	3.812302	0.740644	1.413327
56	1	0	1.738301	0.325762	1.768717
57	6	0	4.909868	0.548840	0.572110
58	1	0	5.625509	-0.367707	-1.244548
59	1	0	3.897321	1.378330	2.287424
60	1	0	5.853804	1.036264	0.795790
61	8	0	-0.223786	0.719391	-1.059505
62	1	0	1.275862	0.858037	-2.809476
63	1	0	-0.393601	-0.058793	0.872925
64	8	0	1.502788	0.200902	-3.488280
65	1	0	1.285066	-0.656888	-3.073351
66	1	0	3.209316	0.504828	-3.682427
67	1	0	2.697439	1.819067	-0.784513
68	8	0	4.118908	0.867611	-3.569410
69	1	0	3.328558	1.929603	-2.187807
70	1	0	4.348603	1.290206	-4.403928

11

Center Number	Atomic Number	Atomic Type	Coordinates (Angstroms) X	Y	Z
1	6	0	-0.689282	-0.357222	-0.301390
2	6	0	-0.873366	-1.836551	-0.067335
3	6	0	-1.613226	-2.607984	-0.965574
4	6	0	-0.306878	-2.438710	1.060874
5	6	0	-1.778530	-3.975626	-0.746420
6	1	0	-2.056314	-2.138728	-1.841517
7	6	0	-0.465206	-3.804856	1.273386
8	1	0	0.264279	-1.831489	1.756576
9	6	0	-1.200273	-4.575865	0.370486
10	1	0	-2.351454	-4.570242	-1.451439
11	1	0	-0.011940	-4.269288	2.143917
12	1	0	-1.320141	-5.642088	0.537820
13	15	0	-1.821328	0.646255	0.778555
14	8	0	-1.752982	0.322618	2.241611
15	15	0	1.810799	-0.164413	-1.139339
16	6	0	2.655364	-1.643390	-0.445026
17	6	0	2.557163	-2.842249	-1.157792
18	6	0	3.296094	-1.633526	0.800802
19	6	0	3.088181	-4.021680	-0.633819
20	1	0	2.047957	-2.856294	-2.118944
21	6	0	3.821117	-2.810383	1.326534
22	1	0	3.374660	-0.704883	1.361271
23	6	0	3.716787	-4.004995	0.609052
24	1	0	3.002721	-4.949777	-1.190717
25	1	0	4.311747	-2.799030	2.295258

26	1	0	4.127027	-4.921820	1.022092
27	6	0	2.935671	1.173333	-0.586179
28	6	0	2.499282	2.221139	0.230330
29	6	0	4.250847	1.179959	-1.069848
30	6	0	3.373311	3.257972	0.562418
31	1	0	1.482328	2.221440	0.611478
32	6	0	5.119669	2.214432	-0.734867
33	1	0	4.601291	0.367105	-1.703339
34	6	0	4.681012	3.258121	0.082615
35	1	0	3.024990	4.064629	1.201034
36	1	0	6.138733	2.206075	-1.109711
37	1	0	5.357984	4.065958	0.343536
38	6	0	-3.460978	0.312346	0.070975
39	6	0	-3.871669	0.808974	-1.170768
40	6	0	-4.301814	-0.536979	0.796131
41	6	0	-5.116743	0.453758	-1.684449
42	1	0	-3.231146	1.481189	-1.736253
43	6	0	-5.547104	-0.890156	0.278847
44	1	0	-3.970774	-0.912513	1.760461
45	6	0	-5.952892	-0.397289	-0.960409
46	1	0	-5.436432	0.844230	-2.645658
47	1	0	-6.198825	-1.549679	0.843403
48	1	0	-6.923442	-0.672240	-1.362303
49	6	0	-1.342895	2.359748	0.409348
50	6	0	-1.305447	3.251435	1.484769
51	6	0	-0.948322	2.789076	-0.863720
52	6	0	-0.890404	4.567955	1.285796
53	1	0	-1.583830	2.899731	2.474224
54	6	0	-0.535234	4.104284	-1.059383
55	1	0	-0.933718	2.100114	-1.704835
56	6	0	-0.506955	4.994142	0.015358
57	1	0	-0.859864	5.256637	2.124521
58	1	0	-0.218621	4.429060	-2.045591
59	1	0	-0.175729	6.016804	-0.137731
60	8	0	0.617195	0.095656	0.016901
61	1	0	-0.940926	-0.123999	-1.348349
